# Characterization of the dual functional effects of heat shock proteins (HSPs) in cancer hallmarks to aid development of HSP inhibitors

**DOI:** 10.1186/s13073-020-00795-6

**Published:** 2020-11-23

**Authors:** Zhao Zhang, Ji Jing, Youqiong Ye, Zhiao Chen, Ying Jing, Shengli Li, Wei Hong, Hang Ruan, Yaoming Liu, Qingsong Hu, Jun Wang, Wenbo Li, Chunru Lin, Lixia Diao, Yubin Zhou, Leng Han

**Affiliations:** 1 Department of Biochemistry and Molecular Biology, McGovern Medical School at The University of Texas Health Science Center at Houston, Houston, TX 77030 USA; 2 Center for Translational Cancer Research, Institute of Biosciences and Technology, Texas A&M University, Houston, TX 77030 USA; 3 Department of Molecular and Cellular Oncology, The University of Texas MD Anderson Cancer Center, Houston, TX 77030 USA; 4 Department of Pediatrics, McGovern Medical School at The University of Texas Health Science Center at Houston, Houston, TX 77030 USA; 5 Department of Bioinformatics and Computational Biology, The University of Texas MD Anderson Cancer Center, Houston, TX 77030 USA

## Abstract

**Background:**

Heat shock proteins (HSPs), a representative family of chaperone genes, play crucial roles in malignant progression and are pursued as attractive anti-cancer therapeutic targets. Despite tremendous efforts to develop anti-cancer drugs based on HSPs, no HSP inhibitors have thus far reached the milestone of FDA approval. There remains an unmet need to further understand the functional roles of HSPs in cancer.

**Methods:**

We constructed the network for HSPs across ~ 10,000 tumor samples from The Cancer Genome Atlas (TCGA) and ~ 10,000 normal samples from Genotype-Tissue Expression (GTEx), and compared the network disruption between tumor and normal samples. We then examined the associations between HSPs and cancer hallmarks and validated these associations from multiple independent high-throughput functional screens, including Project Achilles and DRIVE. Finally, we experimentally characterized the dual function effects of HSPs in tumor proliferation and metastasis.

**Results:**

We comprehensively analyzed the HSP expression landscape across multiple human cancers and revealed a global disruption of the co-expression network for HSPs. Through analyzing HSP expression alteration and its association with tumor proliferation and metastasis, we revealed dual functional effects of HSPs, in that they can simultaneously influence proliferation and metastasis in opposite directions. We experimentally characterized the dual function of two genes, *DNAJC9* and *HSPA14*, in lung cancer cells. We further demonstrated the generalization of this dual direction of associations between HSPs and cancer hallmarks, suggesting the necessity to more carefully evaluate HSPs as therapeutic targets and develop highly specific HSP inhibitors for cancer intervention.

**Conclusions:**

Our study furnishes a holistic view of functional associations of HSPs with cancer hallmarks to aid the development of HSP inhibitors as well as other drugs in cancer therapy.

## Background

Heat shock proteins (HSPs), as one of the largest families of molecular chaperones [1], are traditionally divided into 9 families/sub-families based on their molecular weights: HSP10 (HSPE), HSP20 (HSPB), HSP40 (DNAJA, DNAJB and DNAJC), HSP60 (HSPD), HSP70 (HSPA), HSP90 (HSPC), and large HSPs [2]. HSPs in the same family usually have similar sequences and share functional domains. For example, four members of the HSP90 family, *HSP90AA1*, *HSP90AB1*, *HSP90B1*, and *TRAP1*, have ~ 80% sequence similarity and share three functional domains, an N-terminal ATP-binding domain, a middle linker region, and a C-terminal domain [3]. HSPs often function collaboratively to ensure the correct process of protein folding [4, 5]. For example, HSP40s often act as co-chaperones to transfer premature/misfolded peptides to HSP70s [6], through which the peptide can be correctly folded to make mature proteins [7]. HSPs that demonstrate collaborative relationships are usually co-expressed [8]. For example, *HSPE1* and *HSPD1* are co-expressed to assist protein folding in the mitochondria [9].

Cancer hallmarks are common traits shared by cancers and thus are significant for understanding cancer capability and aiding the development of anti-cancer therapy [10]. HSPs profoundly impact malignant progression across multiple cancer types by manipulating cancer hallmarks [11], including anti-apoptosis [12], proliferation [13], metastasis [5], and angiogenesis [5]. For example, *HSPD1*, an HSP60 member, arrests apoptosis by stabilizing the baculoviral inhibitor of apoptosis repeat-containing protein 5 (*BIRC5*) in breast cancer [14]. *HSPA8*, an HSP70 member, promotes cell proliferation by regulating Ras pathways in colorectal adenocarcinoma [15]. *CRYAB*, an HSP20 member, promotes tumor metastasis by activating the NF-ĸB pathway in gastric cancer [16]. *DNAJA3*, an HSP40 member, modulates angiogenesis by destabilizing *HIF1A* in HeLa cells [17]. Furthermore, HSPs may contribute to two or more cancer hallmarks. For example, *HSP90B1*, an HSP90 member, is associated with proliferation [18], metastasis [19], and angiogenesis [20] across multiple cancers*. HSPA1*, an HSP70 member, is associated with proliferation [21], metastasis [22], and anti-apoptosis [21] in several cancers*.*

HSPs represent promising therapeutic targets due to their significant roles in tumorigenesis. Multiple HSP inhibitors, including 17AAG (HSP90 inhibitor) [23], cmHsp70.1 (HSP70 inhibitor) [24], quercetin (HSP20 inhibitor) [18], and KNK423 (pan-HSP inhibitor) [25], have been developed in recent decades. These drugs have been tested in clinical trials, including a phase II trial of 17AAG in breast cancer [26], phase II trial of 17AAG in melanoma [27], and phase I trial of cmHsp70.1 in lung cancer [28]. However, none has been approved by the US Food and Drug Administration (FDA) for anti-cancer therapy [4]. There are many reasons for these unfortunate failures. For example, these drugs might encounter solubility issues to reach effective dosage in vivo and/or fail to target the specific tissue [29]. A further challenge for HSP drug development is the limited understanding of the expression and functions of HSPs in a context-dependent manner [4]. In this study, we comprehensively analyzed multi-omic data from The Cancer Genome Atlas (TCGA) [30], Cancer Cell Line Encyclopedia (CCLE) [31], Project Achilles [32], and Deep RNAi Interrogation of Viability Effects in Cancer (DRIVE) [33] and performed functional experiments to characterize HSPs across multiple cancer types to aid future rationalized development of improved HSP-targeted anti-cancer therapy.

## Methods

### Data collection

The mRNA expression profiles and clinical features of ~ 10,000 patients across 33 human cancers were downloaded from TCGA data portal (https://portal.gdc.cancer.gov/, Additional file 1: Table S1) [30]. The protein expression profiles of BRCA were downloaded from CPTAC (https://cptac-data-portal.georgetown.edu/cptac/s/S015) [34]. The mRNA expression profiles of ~ 1000 cancer cell lines were downloaded from the CCLE (https://portals.broadinstitute.org/ccle/about) [31]. The expression matrices of 29 normal tissues were downloaded from GTEx (https://www.gtexportal.org/home/, Additional file 1: Table S2) [35]. We used default expression unit from each dataset. For example, TCGA used RNA-Seq by Expectation Maximization (RSEM) [30], GTEx used Transcripts Per Kilobase Million (TPM) [35], and CCLE used Fragments Per Kilobase Million (FPKM) [31]. Due to this reason, we did not compare gene expression across datasets directly. PPI were collected from STRING (https://string-db.org/) [36]. Single gene loss-of-function assays were collected from Project Achilles (https://depmap.org/portal/) [32] and DRIVE (https://depmap.org/portal/) [33].

### Correlation between mRNA and protein in TCGA

Protein expression matrix of TCGA was collected from CPTAC (https://proteomics.cancer.gov/programs/cptac). We only selected samples with both mRNA and protein data in TCGA BRCA for analyses, since only BRCA have enough proteomics data for comparisons. The comparison analyses used Spearman’s correlation and followed by FDR adjustment for *p* value. Instances with *Rs* > 0.3 and FDR < 0.05 were considered as significance.

### Construct co-expression network among HSPs

We collected gene expression data for 20 normal tissues and matched tumor samples from GTEx and TCGA, respectively. We constructed a co-expression network for each cancer type and normal tissue by calculating the correlation between individual HSPs using Spearman’s correlation. Only cancer type/tissue with ≥ 10 samples was analyzed for co-expression. In each cancer type/tissue, we adjust the *p* value with FDR method and considered *Rs* > 0.3 and FDR < 0.05 as significance. Random sampling was performed by R 3.5 (https://cran.r-project.org/). The random sampling sample size is equal to the smaller sample size in tumor or normal tissues.

### Characterize the expression alteration and survival analyses

We used Student’s *t* test to assess the differential expression between TCGA tumor and paired normal samples (Additional file 1: Table S1) and defined |fold change| > 1.5 and FDR < 0.05 as significant, as described in previous studies [13, 37, 38]. Only cancer types with ≥ 5 paired samples were included in these analyses. We used a Cox model and log-rank test to assess whether HSP expression was associated with the OS times in cancer patients and considered FDR < 0.05 to indicate significance. We also used Student’s *t* test for two groups and analysis of variance (ANOVA) for multiple groups to assess the statistical difference of clinically relevant events, including cancer subtype, stage, and grade across independent cancer types, and considered FDR < 0.05 to indicate significance. Only groups with ≥ 5 samples were included in these analyses. All FDR adjustments were calculated in individual cancer type for each clinically relevant event, respectively.

### Estimate the associations between HSPs and tumor proliferation

We used the well-known proliferation marker *ki*67 to reflect tumor proliferation across TCGA samples. We then assessed the association between individual HSPs and proliferation by Spearman’s correlation and considered |*Rs*| > 0.2 and FDR < 0.05 to indicate significance [31]. We also applied this method in cancer cell lines. The single gene loss-of-functions were calculated from Project Achilles in ~ 1000 cancer cell lines [32]. The background proliferation score for each cell line was estimated as $\frac{\sum \limits_i^N{S}^i}{N}$, where *S* is the proliferation score for each gene and *N* is the number of genes applied in the loss-of-function assay. In Project Achilles, the case with increased proliferation is rare, which may be due to the reason that knocking out system is insensitive to tumor suppressors [39–43]. Student’s *t* test was used to assess the different associations with proliferation between individual HSPs and baseline, and cases with difference > 0.5 and FDR < 0.05 were considered to be significant.

### Estimate associations between HSPs and EMT

We divided patient samples into a high group and a low group according to the expression of individual HSP genes across cancer types. For independent cancer type, we divided all sample into two groups via median expression of each HSP genes. For example, to detect the EMT enrichment of HSPA14 in BRCA, we divided all BRCA tumor samples into two groups (HSPA14 high expression vs. HSPA14 low expression) by median expression value of HSPA14. We then calculated the enrichment in comparison of the two groups using GSEA 4.03 (https://www.gsea-msigdb.org/gsea/index.jsp) [32]. The gene set of EMT collected from MSigDB [33]. The background is the whole genome genes. The EMT score for each patient was estimated as $\sum \limits_i^N{M}^i/N-\sum \limits_j^n{E}^j/n$, where *N* is the number of epithelial genes and *n* is the number of mesenchymal genes. The list of epithelial and mesenchymal genes was collected from a previous study [44]. We then calculated Spearman’s correlation between individual HSPs and the EMT score across cancer types and considered |*Rs*| > 0.3 and FDR < 0.05 to be significant.

### Cell culture and gene knockdown

A549 was purchased from the American Type Culture Collection and cultured in DMEM/F12 with 10% fetal bovine serum and 1% penicillin/streptomycin. A549 has been tested and confirmed to be negative for mycoplasma. On-TARGET plus SMART pool DNAJC9 siRNA (L-017868-01-0005) and HSPA14 siRNA (L-021084-01-0005) were purchased from Dharmacon. siRNAs were resuspended in 1 ✕ siRNA buffer (GE Dharmacon) to obtain a 20 μM stock. The cells were transfected with the indicated siRNA at 10 nM final concentrations with DharmaFECT 1 (T-2001-01, GE Dharmacon) according to the manufacturer’s instructions.

### Cell proliferation assay

WST-1 (5015944001, Sigma) was used for the cell proliferation assays according to the manufacturer’s instructions. Briefly, the cells were seeded in 96-well plates and maintained at 37 °C and 5% CO_2_ for 24 h before further processing. Ten microliters per well of cell proliferation reagent WST-1 was added to the cells cultured in 100 μl/well, and the cells were incubated between 0.5~4 h. The amount of formazan dye produced was determined by measuring the absorbance at 450 nm using an imaging reader (Cytation|5, BioTek).

### EMT assay

Antibodies against E-cadherin (ab76055, 1:1000 western blotting [WB]; immunofluorescence [IF], 1:200) and vimentin (ab92547, 1:1000 WB; IF, 1:200) were purchased from Abcam. Secondary antibodies (1:2000) conjugated to horseradish peroxidase were purchased from Santa Cruz Biotechnology. Secondary antibodies for immunofluorescence staining, anti-mouse and anti-rabbit Alexa Fluor 488 and 546, were obtained from Molecular Probes (1:1000).

For the western blotting assays, the cells were washed in phosphate-buffered saline (PBS) 3 times and lysed directly using Cell Signaling Technology lysis buffer (20 mM Tris-HCl (pH 7.5), 150 mM NaCl, 1 mM EDTA, 1 mM EGTA, 1% Triton X-100, 2.5 mM Sodium pyrophosphate, 1 mM β-glycerophosphate) for 30 min at 4 °C. The lysis buffer contained 1 ✕ protease inhibitor cocktail and phosphatase inhibitor cocktail 2 and 3 (Sigma). Lysates were microcentrifuged at 4 °C at maximum speed (10,000*g*) for 10 min. The supernatant was subjected to BCA Protein Assay (Thermo Scientific) to quantify protein levels. The cell lysates were separated on 8~16% gel (M00660, GenScript), transferred to polyvinylidene fluoride membranes, and probed with antibodies.

For immunofluorescence microscopy, the cells were plated on 35-mm glass-bottom dishes (MatTek) and maintained at 37 °C and 5% CO_2_ for 24 h. Cells were washed with 1 ✕ PBS 3 times and fixed in 4% paraformaldehyde for 15 min, permeabilized in 0.5% Triton X-100 for 10 min, blocked with 3.75% BSA in PBS for 1 h at room temperature, and incubated with primary antibody overnight at 4 °C. Secondary antibodies were applied for 1 h at 37 °C, stained with DAPI for 2 min, and washed with PBS three times. Images were acquired on a Nikon confocal system.

## Results

### Global disruption of co-expression network of HSPs in cancer

**Fig. 1:**
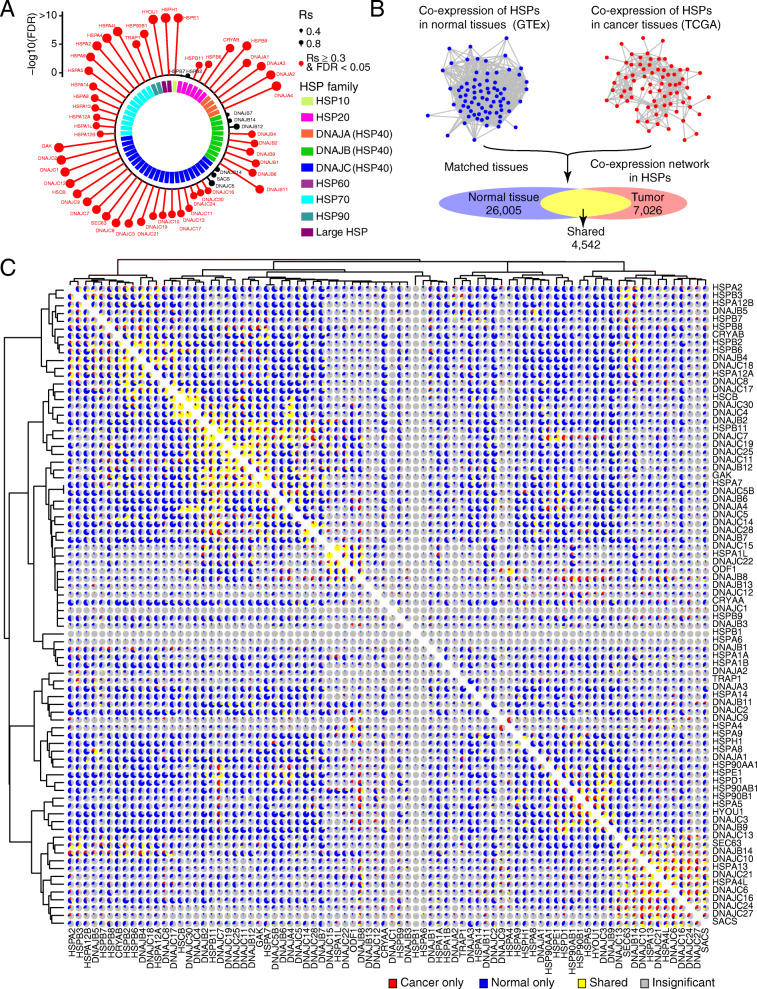
Global disruption of HSP co-expression network in cancer. **a** Expression correlation between mRNA level and protein level of HSP genes in BRCA samples. Red dots denote significant correlation (Rs > 0.3 and |FDR| < 0.05). Colored blocks in inner circle denote HSP family/subfamily. **b** Schematic diagram for HSP co-expression network in normal tissues (blue) and matched cancer types (red). Colored ovals denote number of co-expression pairs detected in normal and tumor tissues (right panel). **c** HSP co-expression network in normal and tumor tissues. Each pie chart denotes percentage of co-expression is cancer tissue only (red), normal tissue only (blue), shared by cancer and normal tissues (yellow), and insignificant ones (gray). The cluster was based on co-expression pattern in cancer

HSP genes included 9 families/sub-families and each family/sub-family has conserved functional domains. To comprehensively characterize HSPs, we collected 82 HSP genes with functional domains and classified into 9 families/sub-families by their molecular weights [5] (Additional file 1: Table S3): HSP10 (one gene), HSP20 (11 genes), HSP40s (48 genes: four, 13 and 31 genes for subfamily DNAJA, DNAJB, and DNAJC, respectively), HSP60 (one gene), HSP70 (15 genes), HSP90 (four genes), and large HSP (two genes). We first investigated the expression of HSPs at both mRNA level and protein level from BRCA patient samples (see the “Methods” section), and we observed that majority of HSPs (49/57, 86.0%) are highly correlated between mRNA and protein level (Spearman’s correlation [Rs] > 0.3 and FDR < 0.05, Fig. 1a), including HSP40 members *DNAJC1* (Rs = 0.73, FDR < 2.2 × 10^−16^), *GAK* (Rs = 0.80, FDR < 2.2 × 10^−16^), HSP70 member *HSPA4* (Rs = 0.76, FDR < 2.2 × 10^−16^), and large HSP member *HYOU1* (Rs = 0.78, FDR < 2.2 × 10^−16^, Additional file 1: Fig. S1A). In the absence of protein expression across large number of cancer samples, our observation suggested that mRNA expression could partially represent the protein expression of HSPs.

Given that HSPs are universally co-expressed to exert their functions [5], we constructed the co-expression network of HSPs in normal tissues and matched tumor tissues. We first characterized the co-expression network by calculating *Rs* among HSPs across 20 normal tissues from The Genotype-Tissue Expression Portal (GTEx) [35] (3321 unique pairs). We detected 26,005 co-expressions from 3064 unique HSP pairs across all tissues, ranging from 28 co-expression in cervix uteri to 2121 co-expression in stomach (Fig. 1b and Additional file 1: Fig. S1B). We collected protein-protein-interaction (PPI) network of HSPs from STRING database and observed that 1684 co-expressed HSPs (1684/3064, 55.0%) showed experimentally validated PPI, suggesting the reliability of our co-expression network (Additional file 1: Fig. S1B). We then constructed the co-expression network of HSPs in 20 matched tumor tissues and detected 7026 co-expressions from 1937 unique HSP pairs across all cancer types, ranging from 57 co-expression in OV to 658 co-expression in TGCT (Fig. 1b and Additional file 1: Fig. S1C). The number of co-expressions was significantly reduced in tumor compared to normal tissues (26,005/7026; 3.70-fold decrease), suggesting lost co-expression of HSPs in cancer. For example, there are 969 co-expressions in normal lung tissue, but only 351 in lung cancer (2.76-fold decrease, Additional file 1: Fig. S1D). A similar scenario was visualized in normal breast tissue versus breast cancer (1417 vs. 245 co-expressions; 5.78-fold decrease, Additional file 1: Fig. S1E). To eliminate the potential effects from sample size, we performed 1000 times random sampling for the same sample size in lung vs. lung cancer and breast vs. breast cancer. We observed that number of co-expressions was still significantly reduced in tumor compared to normal tissues, which further validated our major conclusion that the co-expression network is globally disrupted in cancer (Additional file 1: Fig. S1F). The global disruption of HSP co-expression may affect their functions (Fig. 1c). For example, *DNAJA3* and *HSP90B1*, which correlated with each other in 12 normal tissues, including breast (*Rs* = 0.50, false discovery rate [FDR] = 8.83 × 10^−16^) and lung (*Rs* = 0.49, FDR =1.69 × 10^−13^), had no significant co-expression in tumor tissues (breast invasive carcinoma [BRCA], *Rs* = 0.08, FDR = 0.14, and lung adenocarcinoma [LUAD], *Rs* = − 0.05, FDR = 0.55; Additional file 1: Fig. S1G). Many HSP pairs were co-expressed in both normal and tumor tissues (Fig. 1c). For example, *HSPD1* correlated with *HDPE1* in normal tissues, including breast (*Rs* = 0.73, FDR < 2.2 × 10^−16^) and lung (*Rs* = 0.87, FDR < 2.2 × 10^−16^), as well as in matched cancer types, including BRCA (*Rs* = 0.69, FDR < 2.2 × 10^−16^) and LUAD (*Rs* = 0.83, FDR < 2.2 × 10^−16^, Additional file 1: Fig. S1H). Importantly, we observed 2599 tumor-specific co-expressions from 1371 unique HSP pairs across all cancer types (Fig. 1c). For example, *HSPB1* and *DNAJC30* correlated with each other in eight cancer types, including BRCA (*Rs* = 0.39, FDR < 2.2 × 10^−16^) and LUAD (*Rs* = 0.47, FDR < 2.2 × 10^−16^), but had no significant or reduced co-expression in normal tissues, including breast (*Rs* = − 0.01, FDR = 1) and lung tissue (*Rs* = − 0.23, FDR = 4.6 × 10^−5^, Additional file 1: Fig. S1I). Targeting these tumor-specific co-expressions may provide a novel strategy for the development of HSP inhibitors. Taken together, our results revealed a global disruption of co-expression between individual HSPs, suggesting functional rewiring of the HSP network in cancer.

### Dysregulation of HSP expression in cancer

**Fig. 2:**
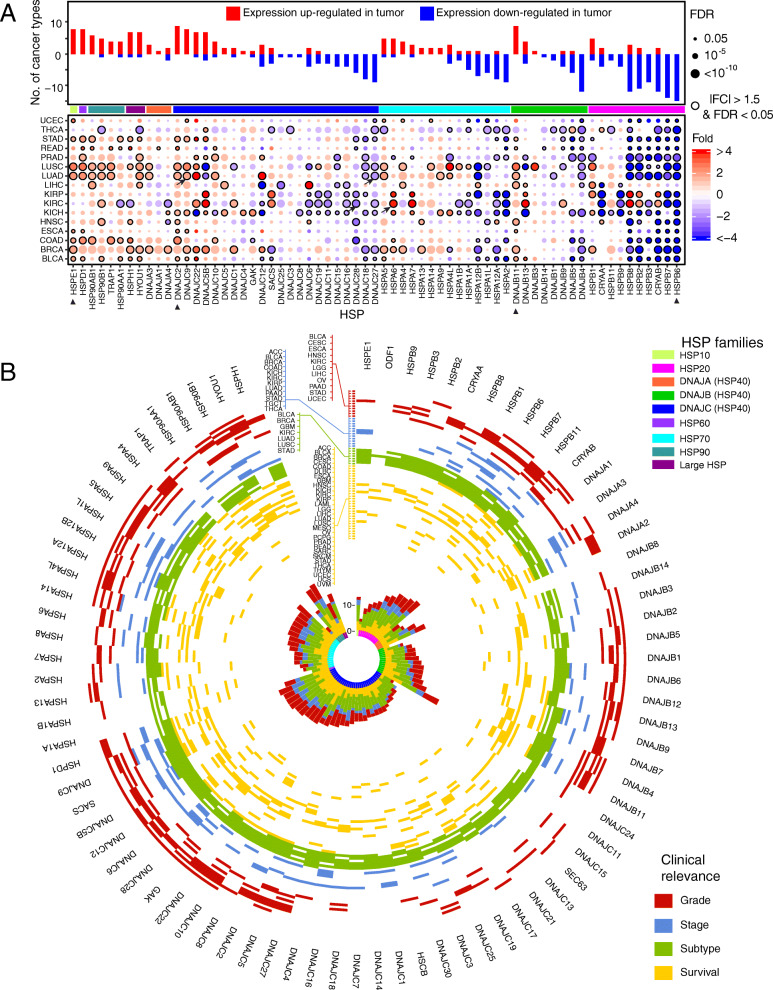
Aberrant expression of HSPs in cancer. **a** Expression alterations of HSPs between tumor and paired normal tissue samples across cancer types. Histogram height denotes the number of cancer types with differentially expressed HSPs. Dots denote upregulation (red) and downregulation (blue). Dots with black edges denote significant difference (|FC| > 1.5 and FDR < 0.05). Multi-colored bar in the middle panel denotes HSP families. **b** Clinically relevant HSPs across human cancers. Colored cells in outer circle denote significant events. Colored bars in middle circle denote numbers of significant cases. Red, blue, green, and yellow denote significant cases in grade, stage, subtype, and survival. Inner circle denotes HSP families

We further investigated aberrant expression of individual HSPs between paired tumor and normal tissue samples across cancer types and defined fold change (FC) > 1.5 and FDR < 0.05 as significantly differential expression. We observed 380 instances of differential expression, including 164 upregulated expressions and 216 downregulated expressions across cancer types (Fig. 2a), suggesting dual roles of HSPs as both tumor-promoting and tumor-suppressing in cancer. Most individual HSPs showed a consistently altered direction across multiple cancer types, suggesting their consistent roles in cancer. For example, *DNAJC9* was upregulated in 8 cancer types, including bladder urothelial carcinoma (BLCA), stomach adenocarcinoma (STAD), and lung squamous cell carcinoma (LUSC), while *DNAJC27* was downregulated in 9 cancer types, including LUAD, kidney renal clear cell carcinoma (KIRC) and BRCA. About 80% (65/82) of HSPs showed aberrant expression, ranging from one cancer type (e.g., *DNAJC25*) to 15 cancer types (e.g., *HSPB6*). More importantly, some expression alterations were consistent with known functions in promoting or repressing tumors. For example, *HSP90AB1*, an oncogene [45], was upregulated in 8 cancer types, while *DNAJB4*, a tumor suppressor [46], was down-regulated in 12 cancer types. We further examined the expression alterations for each HSP family. For example, the HSP90 family, including *HSP90AB1*, *HSP90AA1*, *HSP90B1*, and *TRAP1*, had 21 instances of differential expression, with the majority showing upregulation (19/21 = 90.5%). In contrast, the HSP20 family had 99 instances of differential expression, of which most (85/99 = 85.9%) were downregulated. Some families showed more diverse alterations in gene expression. For example, the HSP70 family had 73 instances of differential expression, 53.4% of which showed upregulation while 46.6% showed downregulation. This may imply that the failure of drugs that inhibit HSP70 is due to the inconsistently aberrant expression patterns of these HSPs in cancer.

Furthermore, the differential expression of individual HSPs was associated with the clinically relevant events [47, 48] across cancers that we observed 1185 significant associations between HSPs and clinical relevance (Fig. 2a and b and Additional file 1: Fig. S2A). For example, upregulated HSPs, including *HSPA6* (Student’s *t* test, FC = 1.82, FDR = 7.0 × 10^−14^) and *DNAJC9* (FC = 3.87, FDR < 2.2 × 10^−16^), were associated with worse overall survival (OS) in KIRC (Kaplan–Meier test, *p* = 1.4 × 10^−4^) and LUAD (*p* = 4.8 × 10^−3^) (Additional file 1: Figs. S2B and S2C). These genes are highly expressed in late stage of KIRC (FDR = 0.001) and LUAD (FDR = 4.8 × 10^−4^), differentially expressed across subtypes of KIRC (FDR = 8.9 × 10^−14^) and LUAD (FDR = 9.3 × 10^−9^), and highly expressed in high grade of KIRC (FDR = 1.4 × 10^−6^, and FDR = 3.6 × 10^−9^) (Additional file 1: Figs. S2D - S2F). In contrast, downregulated HSPs, including *DNAJC28* (FC = − 2.43, FDR < 2.2 × 10^−16^) and *DNAJC27* (FC = − 1.83, FDR = 1.6 × 10^−16^), were associated with worse OS in KIRC (*p* = 8.2 × 10^−3^) and LUAD (*p* = 0.012) (Fig. 2a and b). These genes are highly expressed in early stage of KIRC (FDR = 4.0 × 10^−6^) and LUAD (FDR = 9.3 × 10^−4^), differentially expressed across subtypes of KIRC (FDR = 8.3 × 10^−12^) and LUAD (FDR = 4.4 × 10^−5^), and highly expressed in low grade of KIRC (FDR = 0.0066, and FDR = 2.2 × 10^−7^) (Additional file 1: Figs. S2D - S2F). Taken together, our comprehensive analysis of HSPs across different cancer types demonstrated global alterations and the potential prognostic value of HSPs in cancer.

### Dual functional effects of HSPs in cell proliferation

**Fig. 3:**
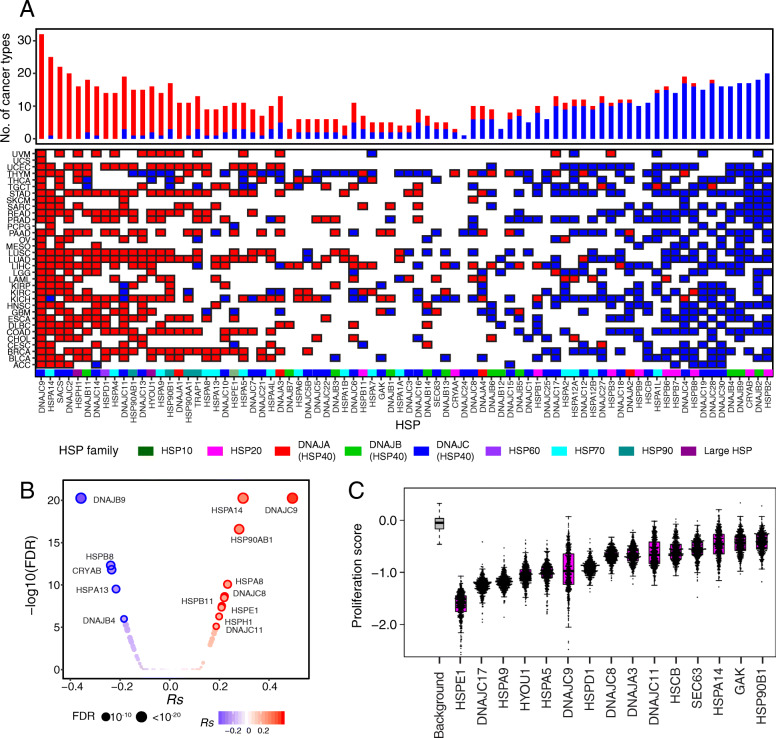
Dual functional effects of HSPs in cell proliferation. **a** Correlation between HSPs and proliferation marker (*ki67*) across cancer types. Histogram height denotes number of cancer types with significant correlation between *ki67* and HSPs. Red and blue colored squares denote positive and negative correlation. Colored bar on bottom denotes HSP families. **b** Correlation between HSPs and *ki67* across ~ 1000 cancer cell lines. **c** Significant decrease in proliferation score in cancer cell lines by knocking out individual HSPs from Project Achilles. Gray box denotes the average proliferation score of all genes tested

Cell proliferation is one of the major hallmarks of tumors [49]. To characterize the functional roles of HSPs in cell proliferation, we calculated the *Rs* between individual HSPs and the well-known proliferation marker *ki67* [50] across cancer types. We identified a total of 920 significant associations, 456 positive associations and 464 negative associations from 97.6% (80/82) of the HSPs, suggesting dual functional effects of HSPs in tumor proliferation (Fig. 3a). Multiple individual HSPs had consistent association with proliferation across cancers, suggesting their consistent roles in promoting or suppressing cell proliferation. For example, *DNAJC9* positively correlated with cell proliferation in 32 cancer types, and *HSPA14* positively correlated with cell proliferation in 24 cancer types. By contrast, *HSPB2* negatively correlated with cell proliferation in 20 cancer types, and *DNAJB2* negatively correlated with cell proliferation in 18 cancer types. To further confirm the functional roles of HSPs in cell proliferation, we analyzed the expression profiles of ~ 1000 cancer cell lines from CCLE [31] and observed similar divergent patterns. For example, in HSP40 families, *DNAJC9* positively correlated with cell proliferation across all cancer cell lines (Fig. 3b) and 14 cancer lineages (Additional file 1: Fig. S3A), while *DNAJB9* negatively correlated with cell proliferation across all cancer cell lines, as well as 13 cancer lineages. Interestingly, HSPs from the same family/subfamily may either promote or suppress cell proliferation. For example, in the HSP70 family, *HSPA4* positively correlated with cell proliferation in 14 cancer types, while *HSPA1L* negatively correlated with cell proliferation in 14 cancer types. In the DNAJB (HSP40) subfamily, *DNAJB11* positively correlated with cell proliferation in 16 cancer types, while *DNAJB9* negatively correlated with cell proliferation in 17 cancer types. Taken together, our results suggest dual functional effects of HSPs in tumor cell proliferation.

To further confirm the functional effects of individual HSPs in cell proliferation, we analyzed the cell proliferation data from Project Achilles, a project experimentally that characterized the effects of genes on cell proliferation by gene knockout [32]. We collected the proliferation score of each cell line for the individual HSP knockout condition and compared it to the background proliferation score (see the “Methods” section). We observed that the knockout of 15 HSPs led to significantly lower proliferation scores (difference ≥ 0.5 and FDR < 0.05; Fig. 3c). For example, the knockout of *HSPE1* (difference = 1.85, FDR < 2.2 × 10^−16^), *HSPA9* (difference = 1.08, FDR < 2.2 × 10^−16^), and *DNAJC9* (difference = 0.99, FDR < 2.2 × 10^−16^)*,* significantly reduced cell proliferation. Similar pattern was observed within cancer cell line linages. For example, knockout of *HSPE1*, *HSPA9*, and *DNAJC9* significantly reduced cell proliferation across 21 cancer cell lineages (Additional file 1: Fig. S3B). We also confirmed the proliferation score from DRIVE, a project experimentally that characterized the effects of cell proliferation by gene knockdown [33]. By analyzing the proliferation score for the knockdown of individual HSPs, we observed that the knockdown of *HSPE1*, *HSPA9*, and *DNAJC9* decreased cell proliferation (difference ≥ 0.5 and FDR < 0.05, Additional file 1: Fig. S3C). Knockdown/out experiments are known to be sensitive to loss-of-functions [51], so these results confirmed the strong effect of aberrant HSP expression, which positively correlated with *ki67* in promoting cell proliferation. Taken together, our multi-dimensional data analysis suggested dual functional effects of HSPs in cell proliferation.

### Dual functional effects of HSPs in cancer metastasis

**Fig. 4:**
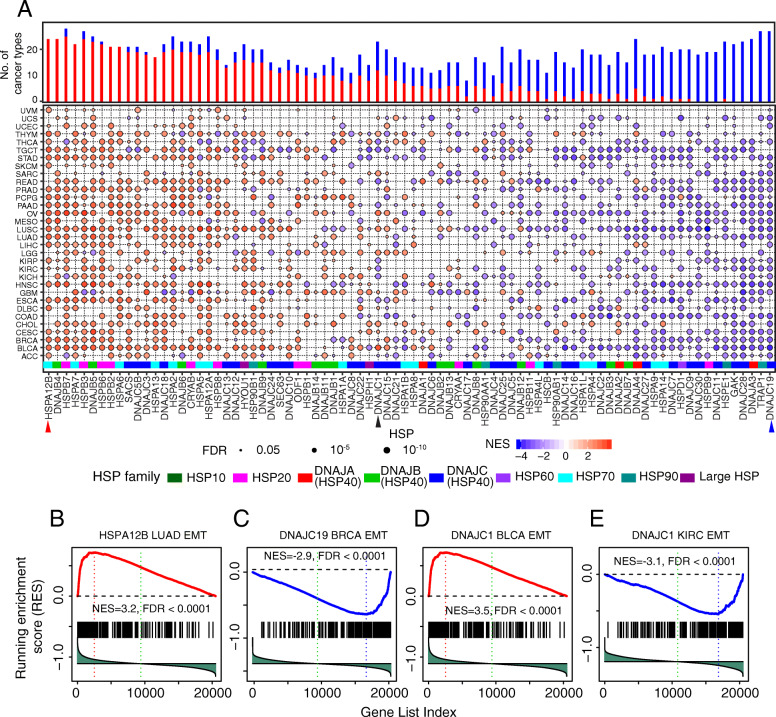
Dual functional effects of HSPs in metastasis. **a** Enrichment of HSPs in EMT. Red and blue circles denote positive and negative enrichment. Histogram height denotes number of cancer types with significant enrichment from HSPs. Colored bar on bottom denotes HSP families. **b**–**d** HSPs with positive enrichment (**b**, **d**) and negative enrichment (**c**, **e**) in EMT module

Metastasis, another significant hallmark of cancer, is the major cause of death among cancer patients [52], and epithelial–mesenchymal transition (EMT) plays a critical role in metastasis [53]. To investigate the functional roles of individual HSPs in metastasis, we assessed their enrichment associated with EMT [54] by comparing highly-expressed and lowly-expressed HSP groups through gene set enrichment analysis (GSEA) and defined FDR < 0.05 as significant (see the “Methods” section) [55]. We identified 1513 significant HSP enrichments (FDR < 0.05), of which 749 were positive enrichments (promoting EMT) and 764 were negative enrichments (suppressing EMT) from all HSPs (100%, 82/82), suggesting dual functional effects of HSPs in cancer metastasis (Fig. 4a). Individual HSPs showed consistent enrichment in EMT, suggesting their consistent roles in promoting or inhibiting metastasis. For example, *HSPA12B* was positively enriched in 23 cancer types, including LUAD (FDR < 0.0001, Fig. 4b). In contrast, *DNAJC19* was negatively enriched in 26 cancer types, including BRCA (FDR < 0.0001, Fig. 4c). Several HSPs were either positively or negatively enriched in EMT in different cancer types. For example, *DNAJC1* was positively enriched in 12 cancers, including BLCA (FDR < 0.0001, Fig. 4d), while it was also negatively enriched in 11 cancers, including KIRC (FDR < 0.0001 Fig. 4e). Of interest, HSPs from the same family/subfamily showed either promotion or suppression of metastasis (Fig. 4a). For example, in the HSP20 family, *HSPB7* was positively enriched in 22 cancer types, while *HSPB9* was negatively enriched in 25 cancer types. In the HSP70 family, *HSPA7* was positively enriched in 19 cancer types, while *HSPA14* was negatively enriched in 19 cancer types. We also performed GSEA analysis in TCGA normal samples and observed that the enrichment score in normal tissues is significantly lower than tumor samples (*p* = 0.0049, Additional file 1: Fig. S4A).

We further confirmed these enrichments through correlation analysis, in which we calculated the EMT score for each TCGA sample, following a previous study [44]. We then estimated Spearman’s correlation between the EMT score and the expression level of individual HSPs across cancer types. We observed patterns similar to those found in the EMT enrichment analysis (Additional file 1: Fig. S4B). For example, *HSPA12B* positively correlated with the EMT score in LUAD (*Rs* = 0.41, FDR < 2.2 × 10^−16^, Additional file 1: Fig. S4C), while *DNAJC19* negatively correlated with the EMT score in BRCA (*Rs* = 0.36, FDR < 2.2 × 10^−16^, Additional file 1: Fig. S4D). *DNAJC1* positively correlated with the EMT score in BLCA (*Rs* = 0.34, FDR = 2.5 × 10^−12^, Additional file 1: Fig. S4E), while it negatively correlated with the EMT score in KIRC (*Rs* = 0.46, FDR < 2.2 × 10^−16^, Additional file 1: Fig. S4F). In the HSP70 family, *HSPB12B* positively correlated with 25 cancer types, while *HSPA9* negatively correlated with 11 cancer types. Taken together, our results demonstrated dual functional effects of HSPs in tumor metastasis.

### Dual functions of individual HSPs in cancer proliferation and metastasis

**Fig. 5:**
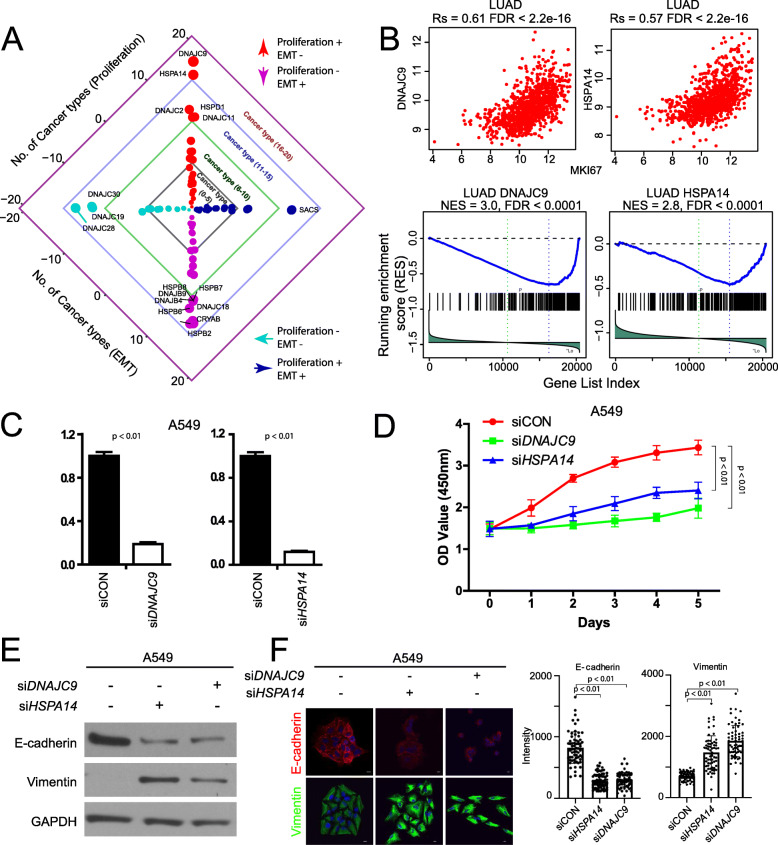
Dual functional effects of individual HSPs in proliferation and metastasis. **a** HSPs with dual functional effects in proliferation and metastasis. Dots denote HSPs with dual functions in promoting both proliferation and metastasis (dark blue), inhibiting both proliferation and metastasis (cyan), promoting proliferation but inhibiting metastasis (red), inhibiting proliferation but promoting metastasis (magenta), respectively. Only instances have significant associations with both proliferation and EMT in a given cancer type were shown. Four arrows denote four directions, including proliferation (+) EMT (+), proliferation (+) EMT (−), proliferation (−) EMT (+), and proliferation (−) EMT (−), respectively. **b** Correlation and enrichment of *DNAJC9* and *HSPA14* with proliferation and EMT. **c** Reverse-transcription polymerase chain reaction was performed to confirm siRNA knockdown of HSPA14 or siDNAJC9. **d** Cell proliferation. Growth rates of A549 cells using WST-1 reagent. Absorbance was measured at *λ* = 450. **e** Western analysis of E-cadherin and vimentin expression. GAPDH was used as loading control. **f** Representative images of A549 cells transfected with siRNA for HSPA14 or DNAJC9 for 48 h and immunostained with E-cadherin (red) or vimentin (green). Scale bars, 10 μm. The plots at the right show the quantification of the intensity of E-cadherin (siCON, *n* = 65 (cells); siHSPA14, *n* = 80; siDNAJC9, *n* = 64) or vimentin (siCON, *n* = 64; siHSPA14, *n* = 64; siDNAJC9, *n* = 64). Error bars denote the standard deviation

The aforementioned analyses revealed dual functional effects among HSPs and HSP families in malignancy, but individual HSPs usually showed consistent associations across cancer types. Therefore, individual HSPs should be valuable targets in cancer treatment. Given that most HSPs correlated with cell proliferation and all HSPs correlated with EMT in human cancers, individual HSPs should present functional roles in either cell proliferation or metastasis. Further analyses showed that 34 HSPs could contemporaneously promote cell proliferation and metastasis in the same cancer type (Fig. 5a and Additional file 1: Fig. S5A), suggesting their potential utility as therapeutic targets in cancer treatment. For example, *SACS*, a DNAJC (HSP40) member, promoted both cell proliferation and EMT in 12 cancer types, including BLCA, head and neck squamous cell carcinoma (HNSC), and LUAD. In contrast, 39 HSPs could contemporaneously suppress cell proliferation and metastasis. For example, *DNAJC28* inhibited both cell proliferation and EMT in 14 cancer types, including BLCA, BRCA, and KIRC. More complicated, we observed 378 instances of opposite directions of promotion vs. suppression from 72 HSPs affecting cell proliferation and EMT (Fig. 5a and Additional file 1: Fig. S5A). For example, *DNAJC9* promoted cell proliferation while inhibiting EMT in 18 cancer types, including BLCA, LUAD, and STAD. *CRYAB*, an HSP20 member, inhibited cell proliferation while inhibiting EMT in 14 cancers, including BRCA, LUAD, and prostate adenocarcinoma (PRAD). We found significantly more instances of opposite HSP functions in cell proliferation and EMT compared to instances of consistent HSP functions (378 vs. 189, *χ*^2^ test, *p* = 1.78 × 10^−8^, Additional file 1: Fig. S5B), which might be a hurdle for the development of HSP inhibitors. Furthermore, the situation is also complicated within each HSP family/subfamily. For example, in the HSP90 family, *HSP90B1* promoted proliferation and EMT, while *HSP90AA1*, *HSP90AB1*, and *TRAP1* promoted proliferation but inhibited EMT in BRCA. In the HSP70 family, *HSPA13* promoted proliferation and EMT, while *HSPA14* promoted proliferation but inhibited EMT in LUAD. This may explain the failure of clinical trials of HSP90 [23] and HSP70 [24] inhibitors.

Among these HSPs, *DNAJC9* and *HSPA14* are two top genes with striking dual functions in that they promoted cell proliferation but inhibited EMT in 18 and 17 cancer types, respectively (i.e., in LUAD, Fig. 5b). We applied siRNAs to knockdown *DNAJC9* or *HSPA14* in A549 cells and successfully repressed these two genes (Fig. 5c, *p* < 0.01), respectively. With efficient reduction of expression, cell proliferation was reduced significantly upon knockdown of *DNAJC9* or *HSPA14* (*p* < 0.01, Fig. 5d). EMT is typically characterized as the loss of epithelial cell adhesion and gain of mesenchymal phenotype [56]. Upon knockdown of *DNAJC9* or *HSPA14*, we observed decreased E-cadherin expression and increased vimentin expression (Fig. 5e), which were confirmed by confocal microscopy (Fig. 5f), suggesting the enhancement of EMT. Taken together, our results demonstrated dual functional roles of individual HSPs in cell proliferation and metastasis. Those dual functional effects indicate that new therapeutic treatments should be carefully designed when targeting individual or multiple HSPs in cancer.

### Functional effects of HSPs associated with cancer hallmarks

**Fig. 6:**
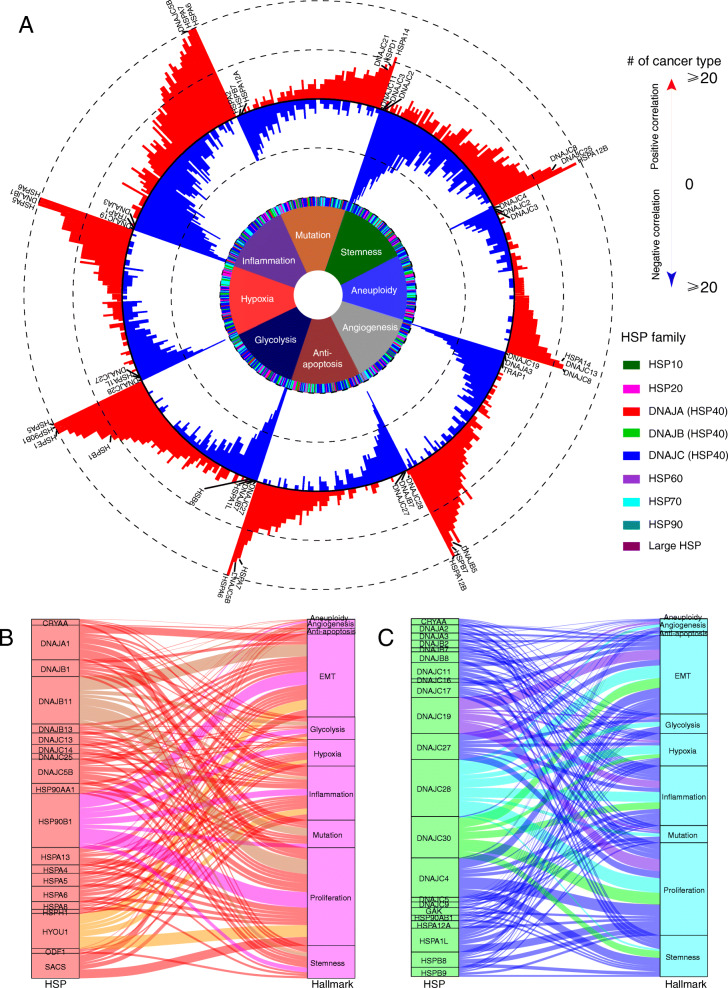
Dual functional effects of individual HSPs among cancer hallmarks. **a** Association between HSPs and eight cancer hallmarks. Red and blue bars in the outer rings denote the number of cancer types with positive and negative associations. The colored rectangles in the middle denote HSP families. The colored sections of the inner ring denote the cancer hallmarks. **b** HSPs positively associated with ≥ 5 cancer hallmarks across cancer types. **c** HSPs negatively associated with ≥ 5 cancer hallmarks across cancer types. The thickness of the curve denotes the number of cancer types with associations between HSPs and cancer hallmarks in **b** and **c**

Beyond cell proliferation and metastasis, we further investigated the functional effects of HSPs associated with the cancer hallmarks of genomic instability and mutation, cancer cell stemness, angiogenesis, anti-apoptosis, glycolysis, hypoxia, and inflammation (see the “Methods” section). The majority of HSPs were associated with these cancer hallmarks (Additional file 1: Fig. S6A) and demonstrated strong functional effects among all these hallmarks (Fig. 6a and Additional file 1: Fig. S6B). HSPs showed dual functional effects across all the hallmarks. For example, 258 positive associations and 234 negative associations were found between HSPs and mutation burden, 375 positive associations and 339 negative associations between HSPs and angiogenesis, and 623 positive associations and 641 negative associations between HSPs and inflammation (Fig. 6a). Furthermore, HSP families also showed dual functional effects across these cancer hallmarks. For example, in the HSP70 family, *HSPA5* and *HSPA6* were positively associated with the hypoxia score in 23 and 20 cancers, respectively, while *HSPA1L* was negatively associated with hypoxia in 19 cancers. In the HSP40 family, *DNAJC25* and *DNAJC8* were positively associated with stemness in 16 and 14 cancers, respectively, while *DNAJC11* and *DNAJC3* were negatively associated with stemness in 25 and 25 cancers, respectively. In the HSP20 family, *HSPB6* was negatively associated with glycolysis in 14 cancers, while *HSPB1* was positively associated with glycolysis in 11 cancers. These results suggest that interactively identifying associations between HSPs and cancer hallmarks is necessary to detect individual HSPs as targets in anti-cancer treatment.

To identify potential candidates, we combined all ten hallmarks included in this study and highlighted individual HSPs that had a consistent direction of effect in ≥ 5 hallmarks. Fifteen genes showed positive associations with hallmarks across 17 cancer types (Fig. 6b). For example, *HSP90B1* was positively associated with 9 cancer hallmarks, such as proliferation [57], EMT [58], and inflammation [59], which is consistent with the findings of other studies. This result suggested that *HSP90B1* is a potential target in anti-cancer treatment and that it will be necessary to design a more specific inhibitor to target *HSP90B1*. Other potential targets include individual HSPs such as *DNAJB11*, an HSP40 member that is positively associated with 8 cancer hallmarks, and *HYOU1*, a large HSP member that is associated with 5 cancer hallmarks. In contrast, we identified 22 individual HSPs that were negatively associated with ≥ 5 hallmarks across 22 cancer types (Fig. 6c). Of interest, 16 (16/22, 72.7%) of them are HSP40 family members, including *DNAJC28*, which was associated with 10 hallmarks across 8 cancers; *DNAJC19*, which was associated with 8 hallmarks across 5 cancers; and *DNAJC30*, which was associated with 6 hallmarks across 5 cancers. That result suggested that these genes are broad suppressors of multiple cancer hallmarks. We ranked HSPs with same direction of associations across multiple cancer hallmarks (Additional file 1: Fig. S6C). For example, HSP90B1 is positively associated with six hallmarks in BRCA, and HYOU1 is positively associated with six hallmarks in LUSC, suggesting they may be candidate for targeted therapy in BRCA and LUSC.

## Discussion

Given the critical role of HSPs in tumorigenesis, multiple HSP inhibitors, including pan-HSP inhibitors (e.g., KNK423 [25]) and HSP family-specific inhibitors (e.g., HSP90i 17AAG [23] and HSP20i quercetin [18]), have been developed during the past decades. Unfortunately, none of them has been approved by the FDA as anti-cancer treatment, which may be due to either poor drug responses or severe side effects [4, 5, 13]. To aid the development of HSP inhibitors, we performed comprehensive analyses in large-scale datasets to understand the features of HSPs. Previous studies showed coordination among HSPs (e.g., HSP40s and HSP70s [6]) in their function as chaperones. Through a comprehensive analysis of a large number of samples across tens of normal tissues and cancer types, we demonstrated a global disruption of the co-expression network of HSPs in tumor samples, suggesting disruption of the chaperone functions of HSPs in tumorigenesis. Furthermore, individual HSPs play significant roles in tumorigenesis. For example, *DNAJA1*, an HSP40 gene, stabilizes mutated p53 isoforms, including p53^R156P^ and p53^R157H^ in multiple cancers [60]. Here, we showed global alterations of HSPs across multiple cancer types, which 79.3% of HSPs showed alterations in at least one cancer type. For example, the HSP90 family was largely upregulated, while the HSP20 family was mainly downregulated. Our results partially explain the failure of pan-HSP inhibitors in anti-cancer therapy.

Drugs recently designed on the basis of an HSP family (e.g., HSP90 inhibitor 17AAG [23] and HSP70 inhibitor cmHsp70.1 [28]) have also failed in clinical trials. Here, we showed the heterogeneity of HSPs within a family. For example, in the HSP40 family, *DNAJC9* promoted cell proliferation, while *DNAJB2* suppressed cell proliferation [51, 61]. Similar to this situation, in the HSP70 family, a druggable HSP family [29], *HSPA12B* promoted metastasis, while *HSPA9* suppressed metastasis. Current HSP70 inhibitors, including 15-DSG [62], MAL3-101 [63], and VER155008 [64], have been designed to target the domain or structure shared across HSP70 members [65]. These HSP70-specific inhibitors may not distinguish *HSPA9* from *HSPA12B*, which may lead to complicated consequences in anti-cancer therapy. Our results further explained the failure of HSP inhibitors designed based on a certain HSP family.

We revealed a much more complicated phenomenon, in that individual HSPs can be contemporaneously involved with different cancer hallmarks. For example, 23 HSPs promoted cell proliferation but suppressed metastasis, while 19 HSPs suppressed cell proliferation but promoted metastasis. Among these, we experimentally validated the dual functions of *DNAJC9* and *HSPA14* in a lung cancer cell line. We further demonstrated the generalization of this pattern across multiple cancer hallmarks, including angiogenesis, hypoxia, and inflammation. Our results demonstrated the complexity of developing HSP inhibitors, even based on individual HSPs. It is necessary to design more specific HSP inhibitors based on selected HSPs. In addition, HSP and hallmark associations may have opposite effects in different subtypes. We investigated PAM50 subtypes of breast cancer, a classical subtype definition, to reveal the associations between HSP and hallmarks across different subtypes (Additional file 1: Fig. S7). We observed the opposite effects in different subtypes. For example, HSPB1 is positively associated with inflammation in basal but negatively associated with inflammation in luminal B. The definition of subtypes is significant and complicated across multiple cancer types [66–69], suggesting the necessity to consider subtype for tumor heterogeneity.

## Conclusions

This study expands our knowledge of the involvement of HSPs in tumorigenesis and may guide the development of HSP inhibitors in the future. More importantly, our research provides a novel paradigm based on integrative analysis for other drug development, including HDAC inhibitors [70] and RAS inhibitors [71].

## Supplementary Information

**Additional file 1:** **Fig. S1.** HSP co-expression network in normal tissue and matched cancer types. **Fig. S2**. Clinically relevant HSPs across human cancers. **Fig. S3**. Associations between HSPs and cell proliferation (*ki67*) in cancer cells. **Fig. S4**. Correlation between HSPs and EMT score. **Fig. S5**. Dual functional effects of HSPs on proliferation and EMT. **Fig. S6**. HSPs associated with cancer hallmark across cancer types. **Fig. S7**. Associations between HSPs and hallmarks across breast cancer subtypes. **Table S1**. Samples across TCGA cancer types. **Table S2**. Samples across GTEx tissues. **Table S3**. HSP genes investigated in this study.

## Data Availability

All the datasets used in our analysis are publicly available; all web links are described in the “Methods” section. All codes are available upon reasonable request. All processed data were available on Github (https://github.com/zzzczeus/HSP) [72]. The mRNA expression profiles and clinical features of 33 human cancers were downloaded from TCGA data portal (https://portal.gdc.cancer.gov/) [30]. The protein expression profiles of BRCA were downloaded from CPTAC (https://cptac-data-portal.georgetown.edu/cptac/s/S015) [34]. The mRNA expression profiles of ~ 1000 cancer cell lines were downloaded from the CCLE (https://portals.broadinstitute.org/ccle/about) [31]. The expression matrices of 29 normal tissues were downloaded from GTEx (https://www.gtexportal.org/home/) [35]. PPI were collected from STRING (https://string-db.org/) [36]. Single gene loss-of-function assays were collect from Project Achilles (https://depmap.org/portal/) [32] and DRIVE (https://depmap.org/portal/) [33].
