# Supplementary material for: Characterization of the dual functional effects of heat shock proteins (HSPs) in cancer hallmarks to aid development of HSP inhibitors
Source: Genome Med. 2020 Nov 23;12:101. doi: 10.1186/s13073-020-00795-6 (PMC7682077; doi:10.1186/s13073-020-00795-6)
Supplement: Supplementary file 1 — Additional file 1: Fig. S1. HSP co-expression network in normal tissue and matched cancer types. Fig. S2. Clinically relevant HSPs across human cancers. Fig. S3. Associations between HSPs and cell proliferation (ki67) in cancer cells. Fig. S4. Correlation between HSPs and EMT score. Fig. S5. Dual functional effects of HSPs on proliferation and EMT. Fig. S6. HSPs associated with cancer hallmark across cancer types. Fig. S7. Associations between HSPs and hallmarks across breast cancer subtypes. Table S1. Samples across TCGA cancer types. Table S2. Samples across GTEx tissues. Table S3. HSP genes investigated in this study. [file 13073_2020_795_MOESM1_ESM.pdf]

Characterization of the dual functional effects of heat shock proteins (HSPs) in cancer hallmarks to aid development of HSP inhibitors

Zhao Zhang[1,6], Ji Jing[2,6], Youqiong Ye[1], Zhiao Chen[1], Ying Jing[1], Shengli Li[1], Wei Hong[1], Hang Ruan[1], Yaoming Liu[1], Qingsong Hu[3], Jun Wang[4], Wenbo Li[1], Chunru Lin[3], Lixia Diao[5,7], Yubin Zhou[2,7], Leng Han[1,7]

[1]Department of Biochemistry and Molecular Biology, McGovern Medical School at The University of Texas Health Science Center at Houston, Houston, TX 77030, USA

[2]Center for Translational Cancer Research, Institute of Biosciences and Technology, Texas A&M University, Houston, TX 77030, USA

[3]Department of Molecular and Cellular Oncology, The University of Texas MD Anderson Cancer Center, Houston, TX 77030, USA

[4]Department of Pediatrics, McGovern Medical School at The University of Texas Health Science Center at Houston, Houston, TX 77030, USA

[5]Department of Bioinformatics and Computational Biology, The University of Texas MD Anderson Cancer Center, Houston, TX 77030, USA

[6]These authors contributed equally.

[7]Correspondence should be addressed to L.H. (leng.han@uth.tmc.edu), Y.Z. (yubinzhou@tamu.edu) or L.D. (ldiao@mdanderson.org)

Figure S1

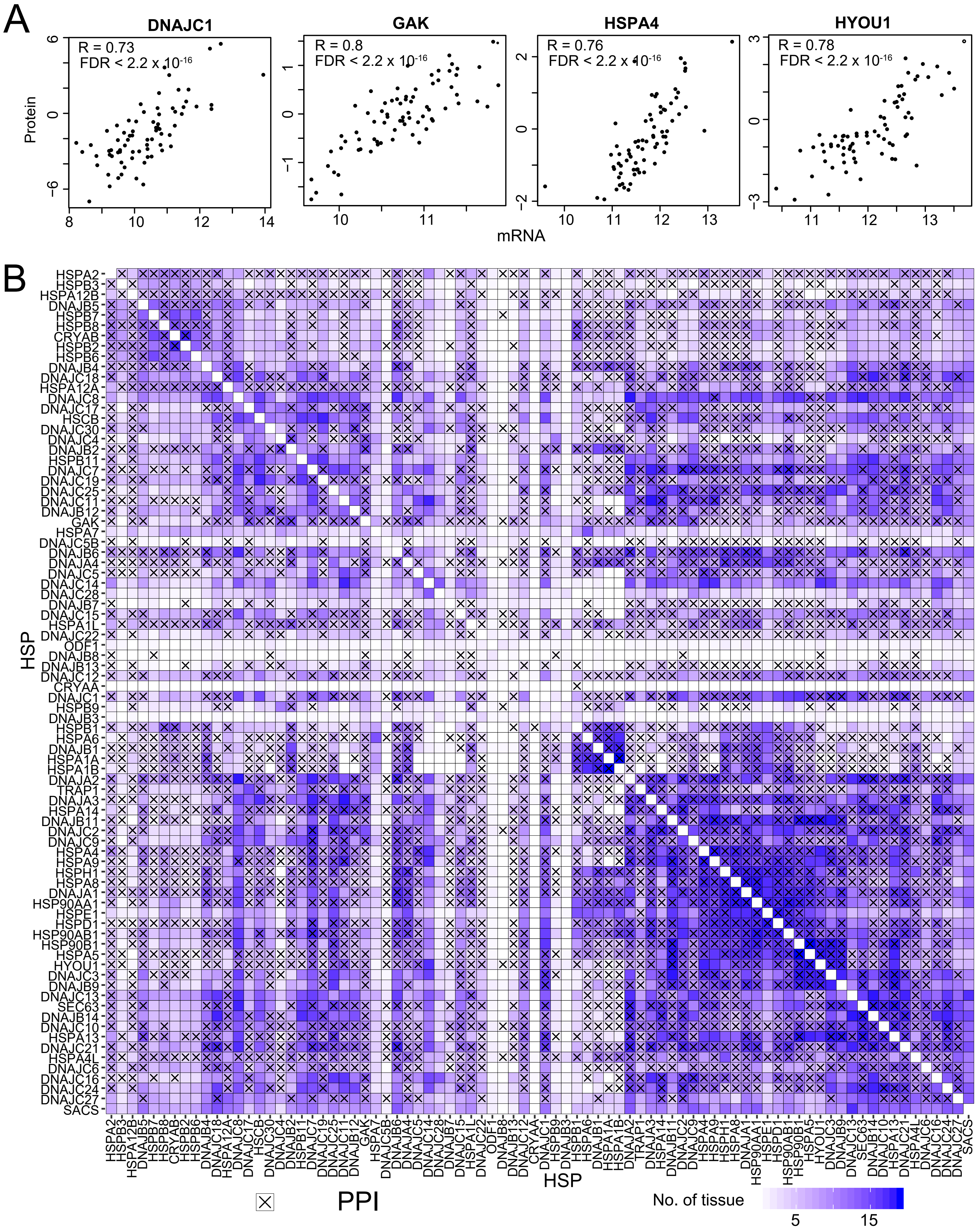
A
DNAJC1
R = 0.73
FDR < 2.2 x 10-16
GAK
R = 0.8
FDR < 2.2 x 10-16
HSPA4
R = 0.76
FDR < 2.2 x 10-16
HYOU1
R = 0.78
FDR < 2.2 x 10-16
Protein
mRNA
B
HSPA2
HSPB3
HSPA12B
DNAJB5
HSPB7
HSPB8
CRYAB
HSPB2
HSPB6
DNAJB4
DNAJC18
HSPA12A
DNAJC8
DNAJC17
HSCB
DNAJC30
DNAJC4
DNAJB2
HSPB11
DNAJC7
DNAJC19
DNAJC25
DNAJC11
DNAJB12
GAK
HSPA7
DNAJC5B
DNAJB6
DNAJA4
DNAJC5
DNAJC14
DNAJC28
DNAJB7
DNAJC15
HSPA1L
DNAJC22
ODF1
DNAJB8
DNAJB13
DNAJC12
CRYAA
DNAJC1
HSPB9
DNAJB3
HSPB1
HSPA6
DNAJB1
HSPA1A
HSPA1B
DNAJA2
TRAP1
DNAJA3
HSPA14
DNAJB11
DNAJC2
DNAJC9
HSPA4
HSPA9
HSPH1
HSPA8
DNAJA1
HSP90AA1
HSPE1
HSPD1
HSP90AB1
HSP90B1
HSPA5
HYOU1
DNAJC3
DNAJB9
DNAJC13
SEC63
DNAJB14
DNAJC10
HSPA13
DNAJC21
HSPA4L
DNAJC6
DNAJC16
DNAJC24
DNAJC27
SACS
HSP
HSP
PPI
No. of tissue
5
15

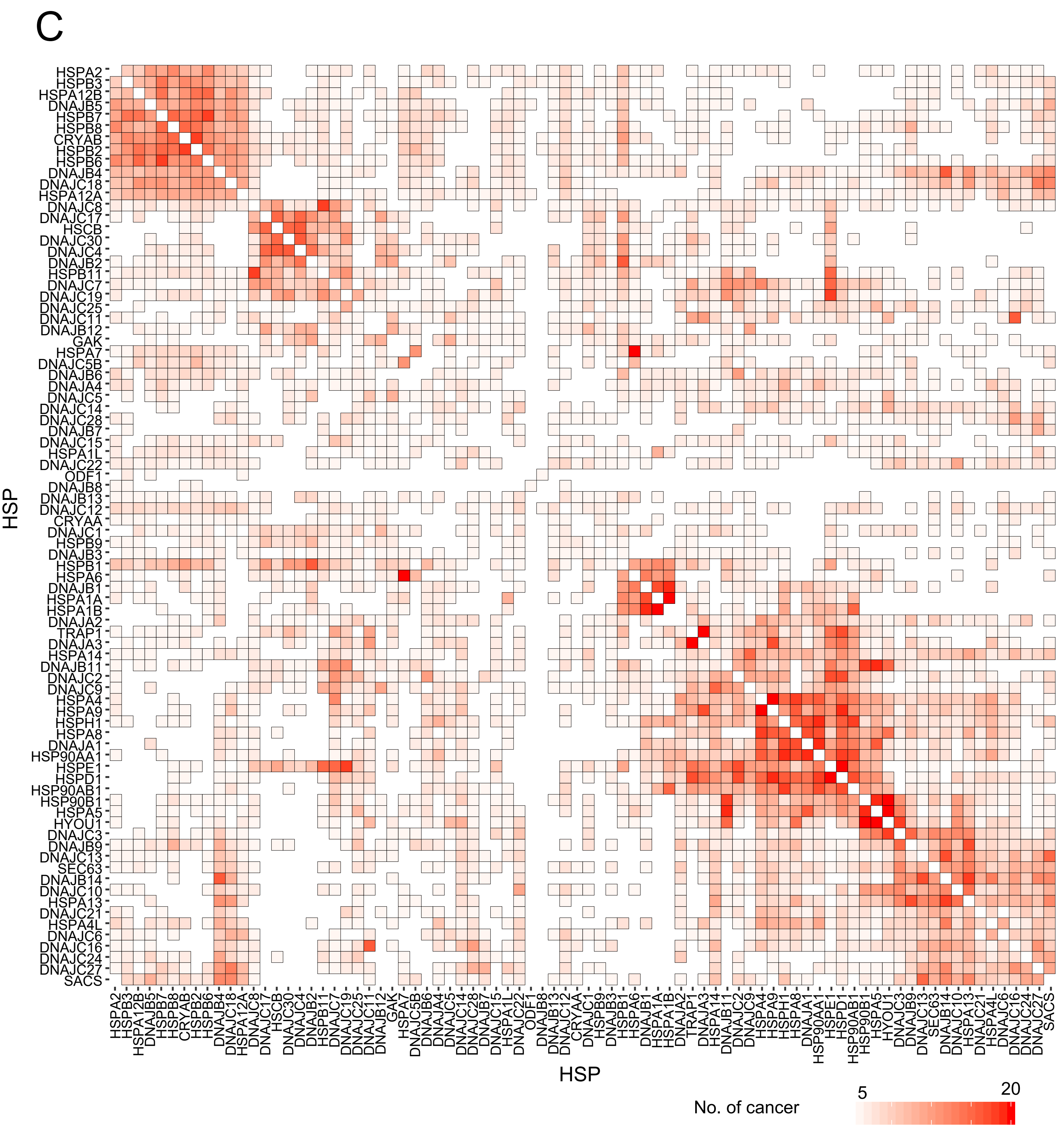
C
HSPA2
HSPB3
HSPA12B
DNAJB5
HSPB7
HSPB8
CRYAB
HSPB2
HSPB6
DNAJB4
DNAJC18
HSPA12A
DNAJC8
DNAJC17
HSCB
DNAJC30
DNAJC4
DNAJB2
HSPB11
DNAJC7
DNAJC19
DNAJC25
DNAJC11
DNAJB12
GAK
HSPA7
DNAJC5B
DNAJB6
DNAJA4
DNAJC5
DNAJC14
DNAJC28
DNAJB7
DNAJC15
HSPA1L
DNAJC22
ODF1
DNAJB8
DNAJB13
DNAJC12
CRYAA
DNAJC1
HSPB9
DNAJB3
HSPB1
HSPA6
DNAJB1
HSPA1A
HSPA1B
DNAJA2
TRAP1
DNAJA3
HSPA14
DNAJB11
DNAJC2
DNAJC9
HSPA4
HSPA9
HSPH1
HSPA8
DNAJA1
HSP90AA1
HSPE1
HSPD1
HSP90AB1
HSP90B1
HSPA5
HYOU1
DNAJC3
DNAJB9
DNAJC13
SEC63
DNAJB14
DNAJC10
HSPA13
DNAJC21
HSPA4L
DNAJC6
DNAJC16
DNAJC24
DNAJC27
SACS
HSP
HSP
No. of cancer
5
20

D

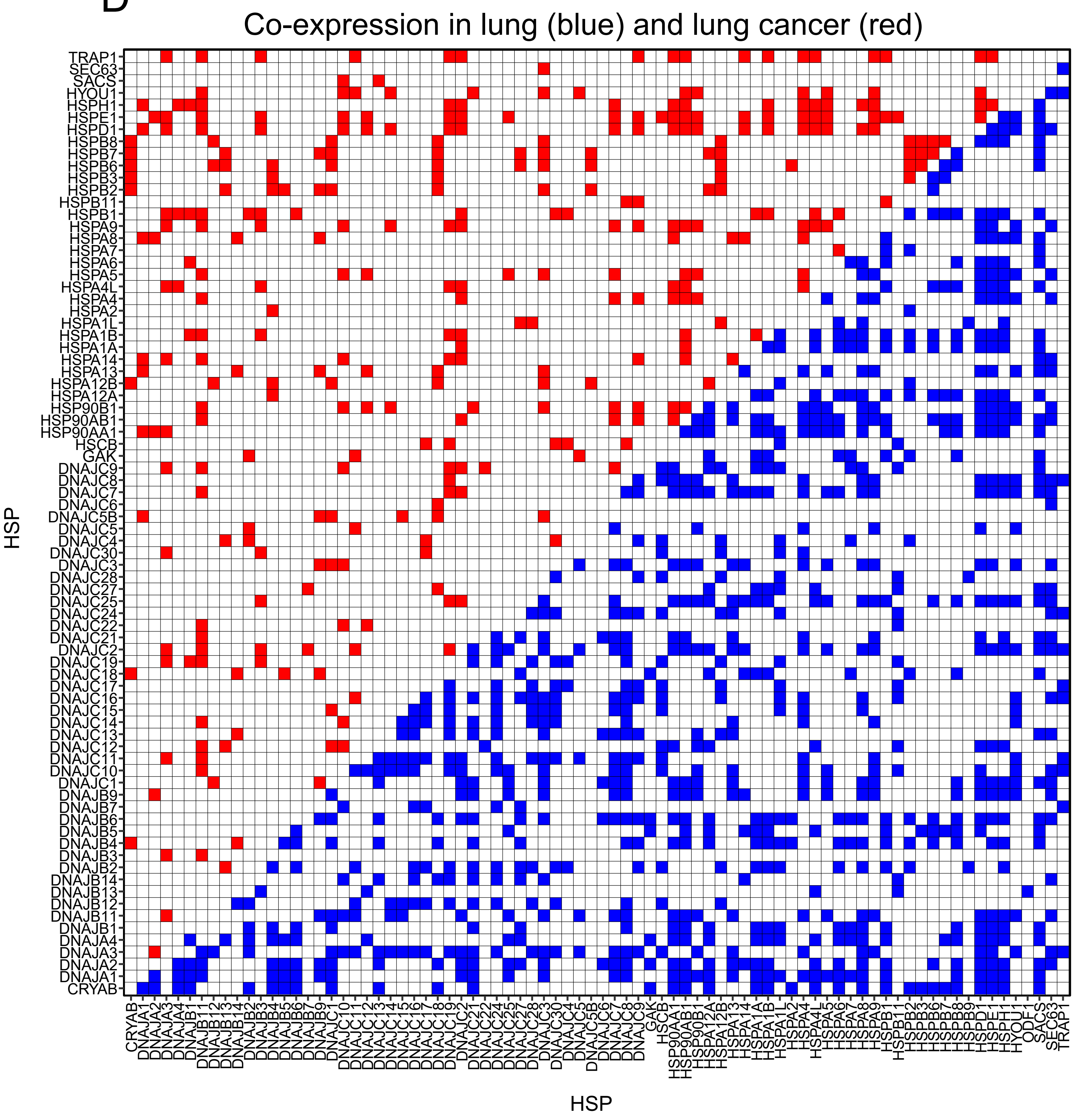

E

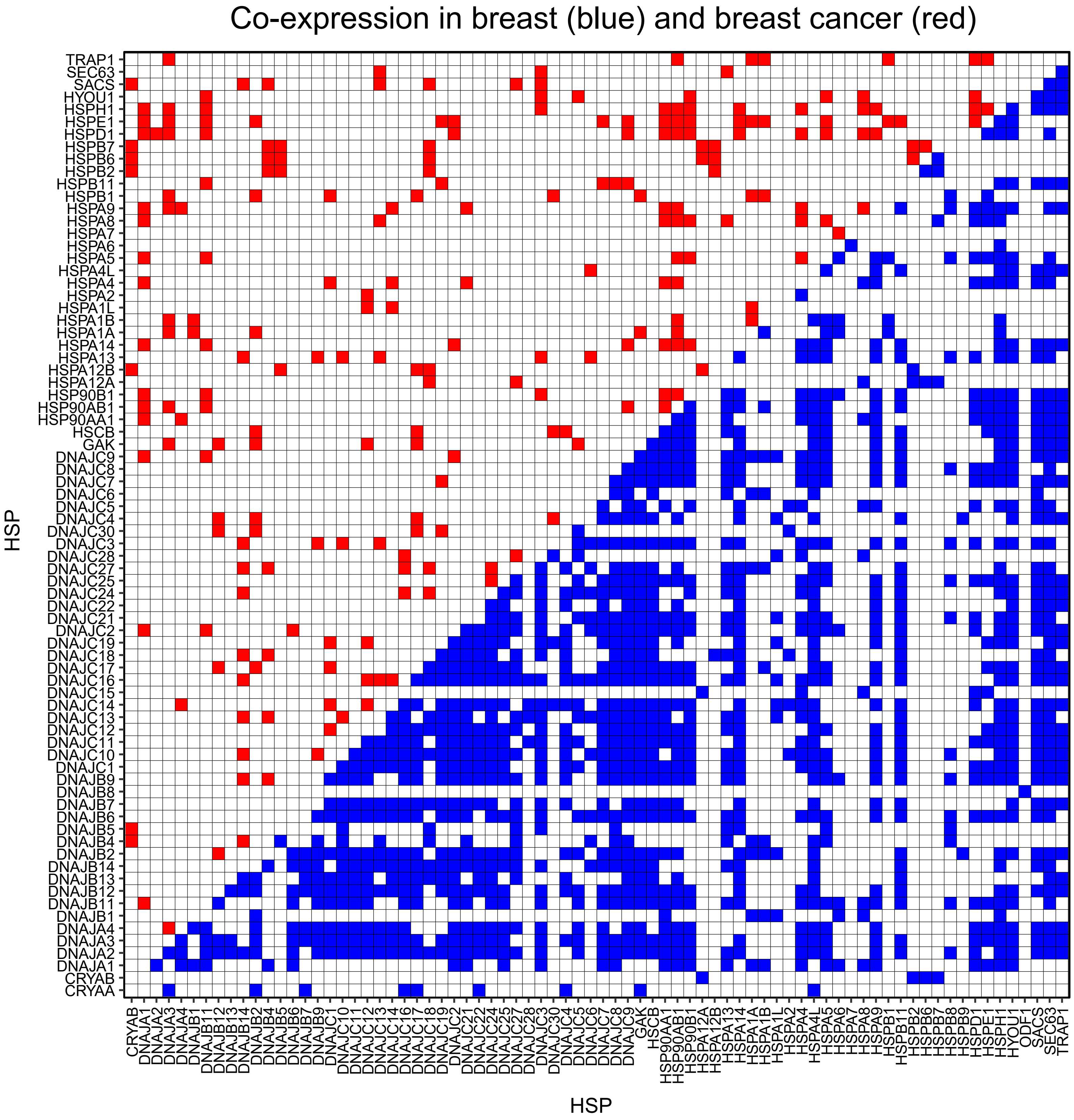
Co-expression in breast (blue) and breast cancer (red)
HSP
HSP
TRAP1
SEC63
SACS
HYOU1
HSPH1
HSPE1
HSPD1
HSPB7
HSPB6
HSPB2
HSPB11
HSPB1
HSPA9
HSPA8
HSPA7
HSPA6
HSPA5
HSPA4L
HSPA4
HSPA2
HSPA1L
HSPA1B
HSPA1A
HSPA14
HSPA13
HSPA12B
HSPA12A
HSP90B1
HSP90AB1
HSP90AA1
HSCB
GAK
DNAJC9
DNAJC8
DNAJC7
DNAJC6
DNAJC5
DNAJC4
DNAJC30
DNAJC3
DNAJC28
DNAJC27
DNAJC25
DNAJC24
DNAJC22
DNAJC21
DNAJC2
DNAJC19
DNAJC18
DNAJC17
DNAJC16
DNAJC15
DNAJC14
DNAJC13
DNAJC12
DNAJC11
DNAJC10
DNAJC1
DNAJB9
DNAJB8
DNAJB7
DNAJB6
DNAJB5
DNAJB4
DNAJB2
DNAJB14
DNAJB13
DNAJB12
DNAJB11
DNAJB1
DNAJA4
DNAJA3
DNAJA2
DNAJA1
CRYAB
CRYAA
ODF1

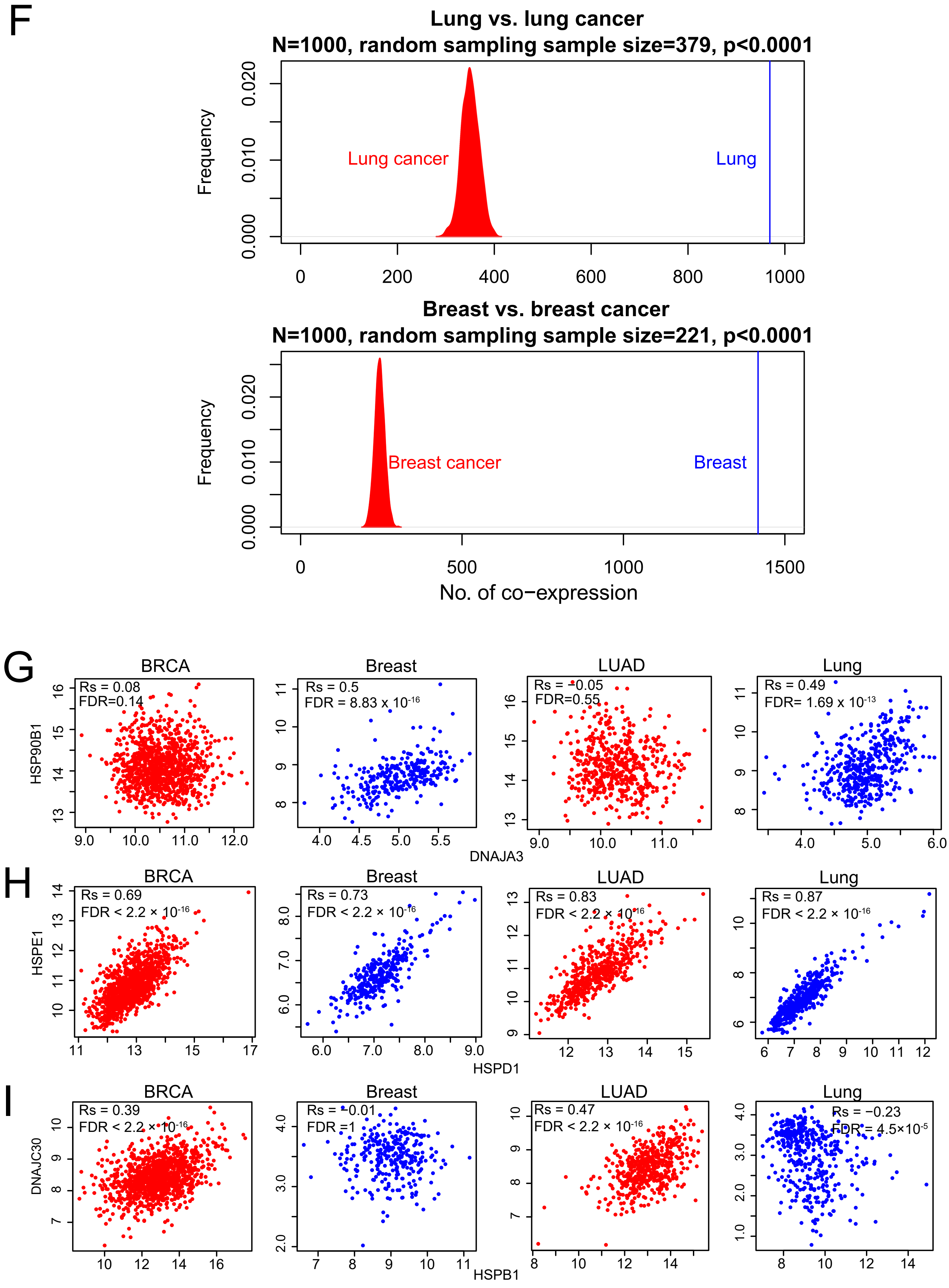
F
Lung vs. lung cancer
N=1000, random sampling sample size=379, p<0.0001
Frequency
Lung cancer
Lung
Breast vs. breast cancer
N=1000, random sampling sample size=221, p<0.0001
Breast cancer
Breast
No. of co−expression
G
BRCA
Rs = 0.08
FDR=0.14
Breast
Rs = 0.5
FDR = 8.83 x 10-16
LUAD
Rs = -0.05
FDR=0.55
Lung
Rs = 0.49
FDR= 1.69 x 10-13
HSP90B1
DNAJA3
H
BRCA
Rs = 0.69
FDR < 2.2 × 10-16
Breast
Rs = 0.73
FDR < 2.2 × 10-16
LUAD
Rs = 0.83
FDR < 2.2 × 10-16
Lung
Rs = 0.87
FDR < 2.2 × 10-16
HSPE1
HSPD1
I
BRCA
Rs = 0.39
FDR < 2.2 × 10-16
Breast
Rs = -0.01
FDR =1
LUAD
Rs = 0.47
FDR < 2.2 × 10-16
Lung
Rs = −0.23
FDR = 4.5×10-5
DNAJC30
HSPB1

Figure S1. HSP co-expression network in normal tissue and matched cancer types. A) HSP genes with significant correlation between mRNA and protein levels. **B)** HSP co-expression network (colored by blue) and experimental PPI network (marked by cross) in normal tissues. **C)** HSP co-expression network in cancer types. **D)** HSP co-expression network in normal lung tissue and lung cancer. **E)** HSP co-expression network in normal breast tissue and breast cancer. **F)** Random sampling test in breast vs. breast cancer (random sampling sample size = 221) and lung vs. lung cancers (random sampling sample size = 379) further support the global disruption of co-expression in cancer. **G)** *HSP90B1* co-expressed with *DNAJA3* in normal tissue but not in tumor. **H)** *HSPD1* co-expressed with *HSPE1* in normal tissue and tumor. **I)** *DNAJC30* co-expressed with *HSPB1* in tumor but not in normal tissue.

Figure S2

A

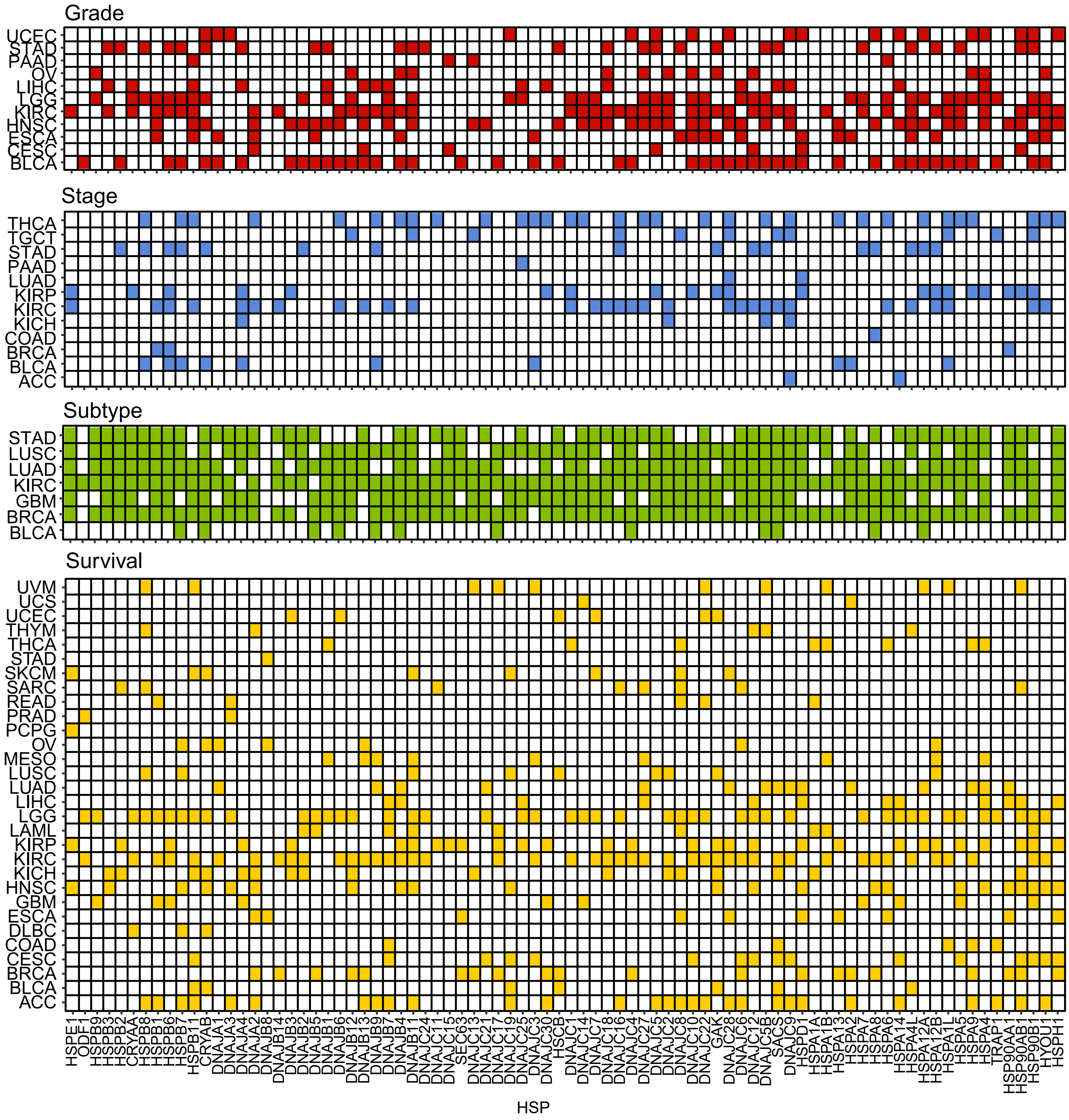
Grade
UCEC
STAD
PAAD
OV
LIHC
LGG
KIRC
HNSC
ESCA
CESC
BLCA
Stage
THCA
TGCT
STAD
PAAD
LUAD
KIRP
KIRC
KICH
COAD
BRCA
BLCA
ACC
Subtype
STAD
LUSC
LUAD
KIRC
GBM
BRCA
BLCA
Survival
UVM
UCS
UCEC
THYM
THCA
STAD
SKCM
SARC
READ
PRAD
PCPG
OV
MESO
LUSC
LUAD
LIHC
LGG
LAML
KIRP
KIRC
KICH
HNSC
GBM
ESCA
DLBC
COAD
CESC
BRCA
BLCA
ACC
HSPE1
ODF1
HSPB9
HSPB3
HSPB2
CRYAA
HSPB8
HSPB1
HSPB6
HSPB7
HSPB11
CRYAB
DNAJA1
DNAJA3
DNAJA4
DNAJA2
DNAJB8
DNAJB14
DNAJB3
DNAJB2
DNAJB5
DNAJB1
DNAJB6
DNAJB12
DNAJB13
DNAJB9
DNAJB7
DNAJB4
DNAJB11
DNAJC24
DNAJC11
DNAJC15
SEC63
DNAJC13
DNAJC21
DNAJC17
DNAJC19
DNAJC25
DNAJC3
DNAJC30
HSCB
DNAJC1
DNAJC14
DNAJC7
DNAJC18
DNAJC16
DNAJC4
DNAJC27
DNAJC5
DNAJC2
DNAJC8
DNAJC10
DNAJC22
GAK
DNAJC28
DNAJC6
DNAJC12
DNAJC5B
SACS
DNAJC9
HSPD1
HSPA1A
HSPA1B
HSPA13
HSPA2
HSPA7
HSPA8
HSPA6
HSPA14
HSPA4L
HSPA12A
HSPA12B
HSPA1L
HSPA5
HSPA9
HSPA4
TRAP1
HSP90AA1
HSP90AB1
HSP90B1
HYOU1
HSPH1
HSP

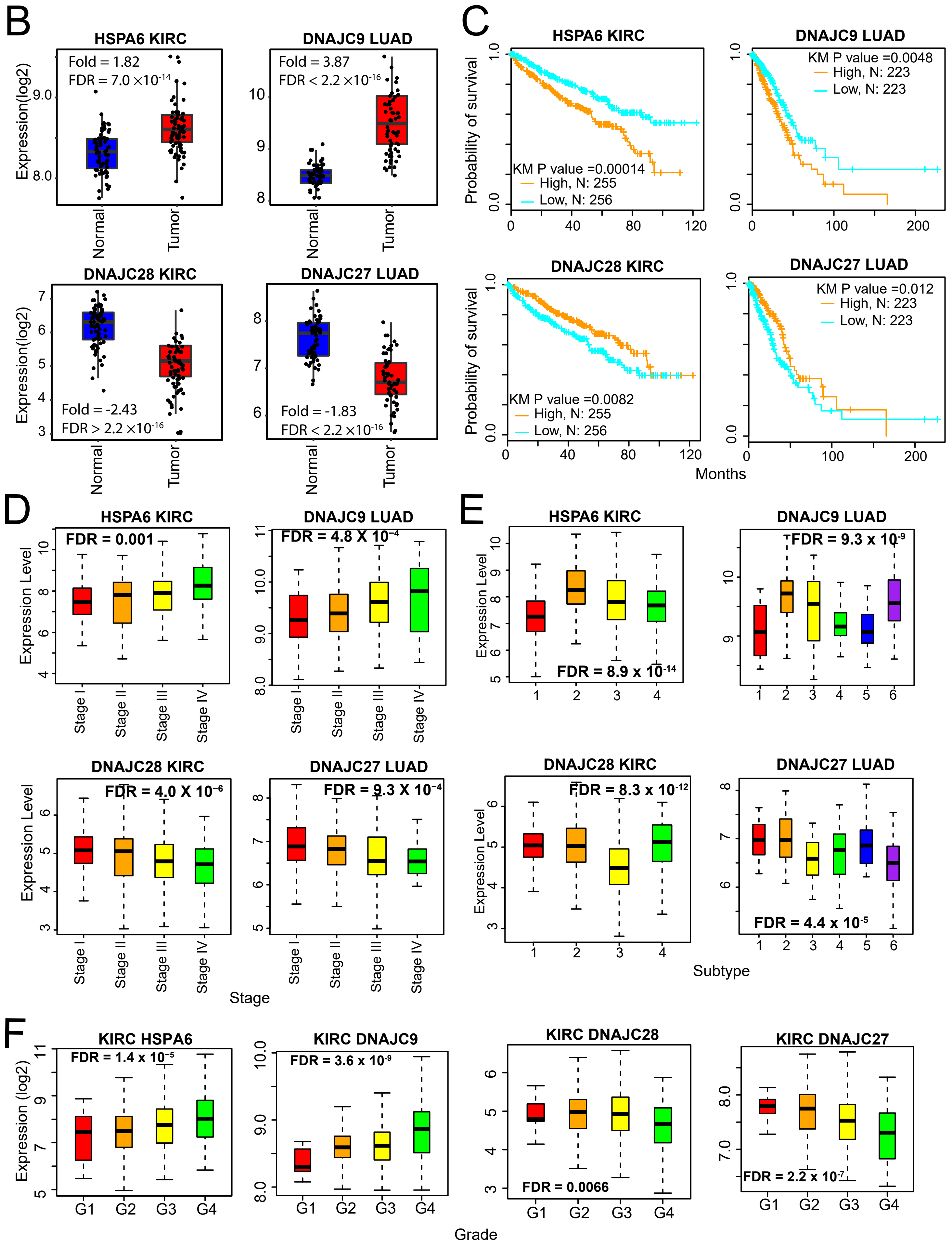
B
HSPA6 KIRC
Fold = 1.82
FDR = 7.0 ×10^-14
DNAJC9 LUAD
Fold = 3.87
FDR < 2.2 ×10^-16
DNAJC28 KIRC
Fold = -2.43
FDR > 2.2 ×10^-16
DNAJC27 LUAD
Fold = -1.83
FDR < 2.2 ×10^-16
Expression(log2)
Normal
Tumor
C
HSPA6 KIRC
KM P value =0.00014
High, N: 255
Low, N: 256
DNAJC9 LUAD
KM P value =0.0048
High, N: 223
Low, N: 223
DNAJC28 KIRC
KM P value =0.0082
High, N: 255
Low, N: 256
DNAJC27 LUAD
KM P value =0.012
High, N: 223
Low, N: 223
Probability of survival
Months
D
HSPA6 KIRC
FDR = 0.001
DNAJC9 LUAD
FDR = 4.8 X 10^-4
DNAJC28 KIRC
FDR = 4.0 X 10^-6
DNAJC27 LUAD
FDR = 9.3 X 10^-4
Expression Level
Stage I
Stage II
Stage III
Stage IV
Stage
E
HSPA6 KIRC
FDR = 8.9 x 10^-14
DNAJC9 LUAD
FDR = 9.3 x 10^-9
DNAJC28 KIRC
FDR = 8.3 x 10^-12
DNAJC27 LUAD
FDR = 4.4 x 10^-5
Expression Level
Subtype
F
KIRC HSPA6
FDR = 1.4 x 10^-5
KIRC DNAJC9
FDR = 3.6 x 10^-9
KIRC DNAJC28
FDR = 0.0066
KIRC DNAJC27
FDR = 2.2 x 10^-7
Expression (log2)
G1
G2
G3
G4
Grade

Figure S2. Clinically relevant HSPs across human cancers. A) Clinically relevant HSPs across human cancers. Red, blue, green, and yellow denote significant cases in grade, stage, subtype, and survival. **B)** Expression alterations of *HSPA4*, *DNAJC28* in KIRC and *DNAJC9*, *DNAJC27* in LUAD. **C)** Expression alterations of HSPs are associated with patient survival. **D)** Expression alternations of HSPs are associated with cancer stages. **E)** Expression alterations of HSPs are associated with cancer subtypes. **F)** Expression alternations of HSPs are associated with cancer grades.

Figure S3

A

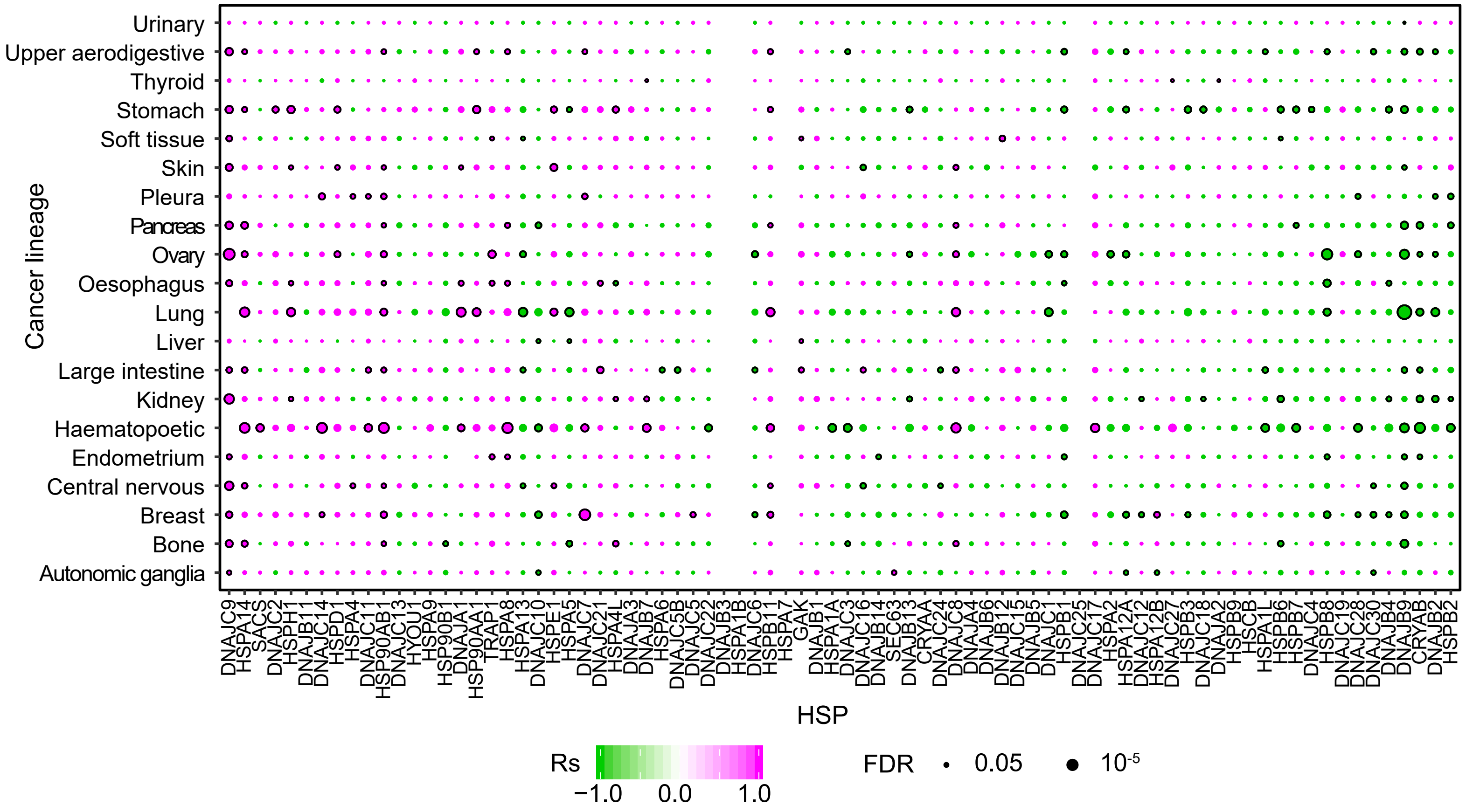
Urinary
Upper aerodigestive
Thyroid
Stomach
Soft tissue
Skin
Pleura
Pancreas
Ovary
Oesophagus
Lung
Liver
Large intestine
Kidney
Haematopoetic
Endometrium
Central nervous
Breast
Bone
Autonomic ganglia
Cancer lineage
DNAJC9 HSPA14 SACS DNAJC2 HSPH1 DNAJB11 DNAJC14 HSPD1 HSPA4 DNAJC11 HSP90AB1 DNAJC13 HYOU1 HSPA9 HSP90B1 DNAJA1 HSP90AA1 TRAP1 HSPA8 HSPA13 DNAJC10 HSPE1 HSPA5 DNAJC7 DNAJC21 HSPA4L DNAJA3 DNAJB7 HSPA6 DNAJC5B DNAJC22 DNAJB3 HSPA1B DNAJC6 HSPB11 HSPA7 GAK DNAJB1 HSPA1A DNAJC3 DNAJC16 DNAJB14 SEC63 DNAJB13 CRYAA DNAJC24 DNAJC8 DNAJA4 DNAJB6 DNAJB12 DNAJC15 DNAJB5 DNAJC1 HSPB1 DNAJC25 DNAJC17 HSPA2 HSPA12A DNAJC12 HSPA12B DNAJC27 HSPB3 DNAJC18 DNAJA2 HSPB9 HSCB HSPA1L HSPB6 HSPB7 DNAJC4 HSPB8 DNAJC19 DNAJC28 DNAJC30 DNAJB4 DNAJB9 CRYAB DNAJB2 HSPB2
HSP
Rs
−1.0 0.0 1.0
FDR
0.05
10-5

B

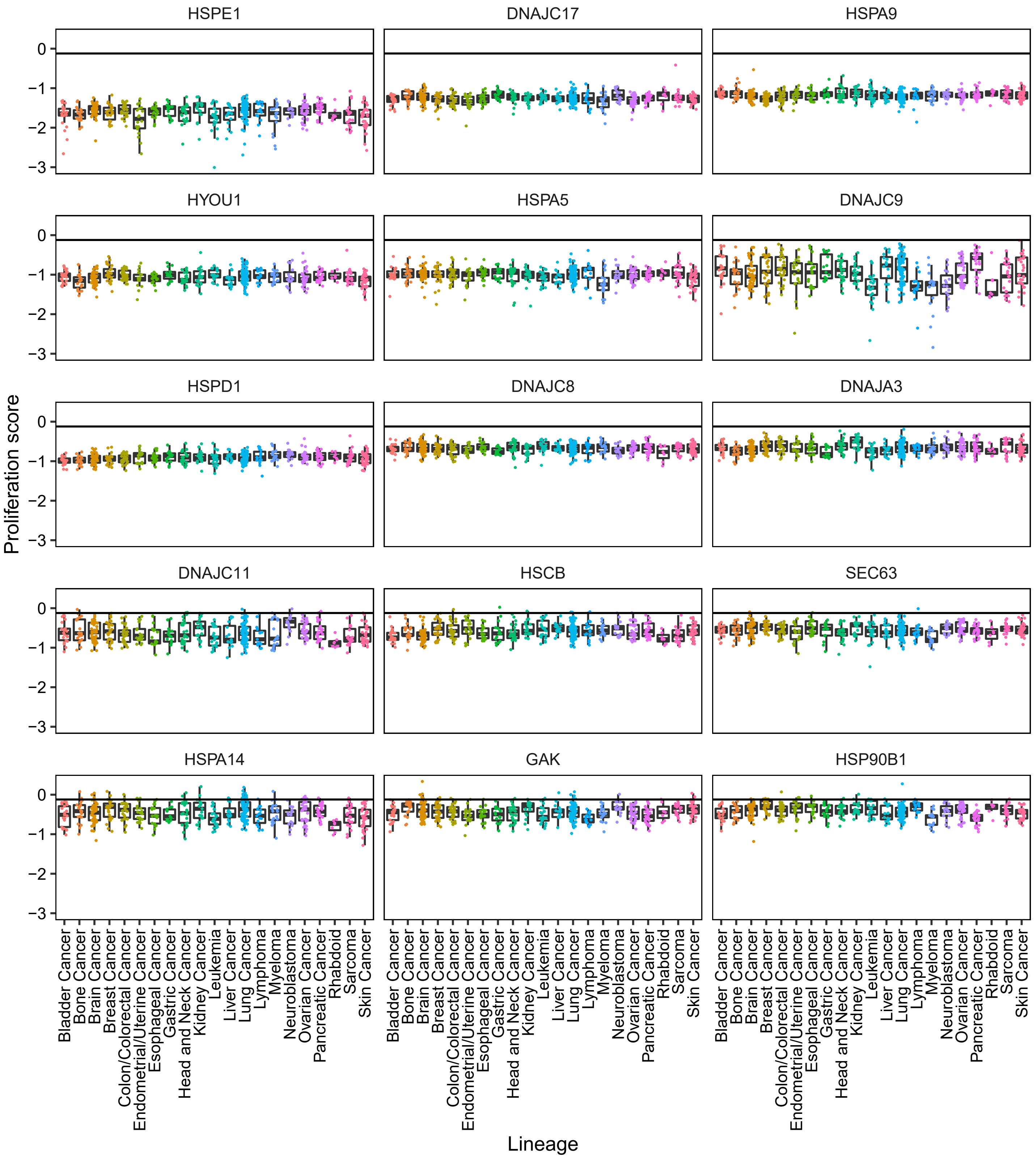

HSPE1
DNAJC17
HSPA9
HYOU1
HSPA5
DNAJC9
HSPD1
DNAJC8
DNAJA3
DNAJC11
HSCB
SEC63
HSPA14
GAK
HSP90B1
Proliferation score
0
−1
−2
−3
Bladder Cancer
Bone Cancer
Brain Cancer
Breast Cancer
Colon/Colorectal Cancer
Endometrial/Uterine Cancer
Esophageal Cancer
Gastric Cancer
Head and Neck Cancer
Kidney Cancer
Leukemia
Liver Cancer
Lung Cancer
Lymphoma
Myeloma
Neuroblastoma
Ovarian Cancer
Pancreatic Cancer
Rhabdoid
Sarcoma
Skin Cancer
Lineage

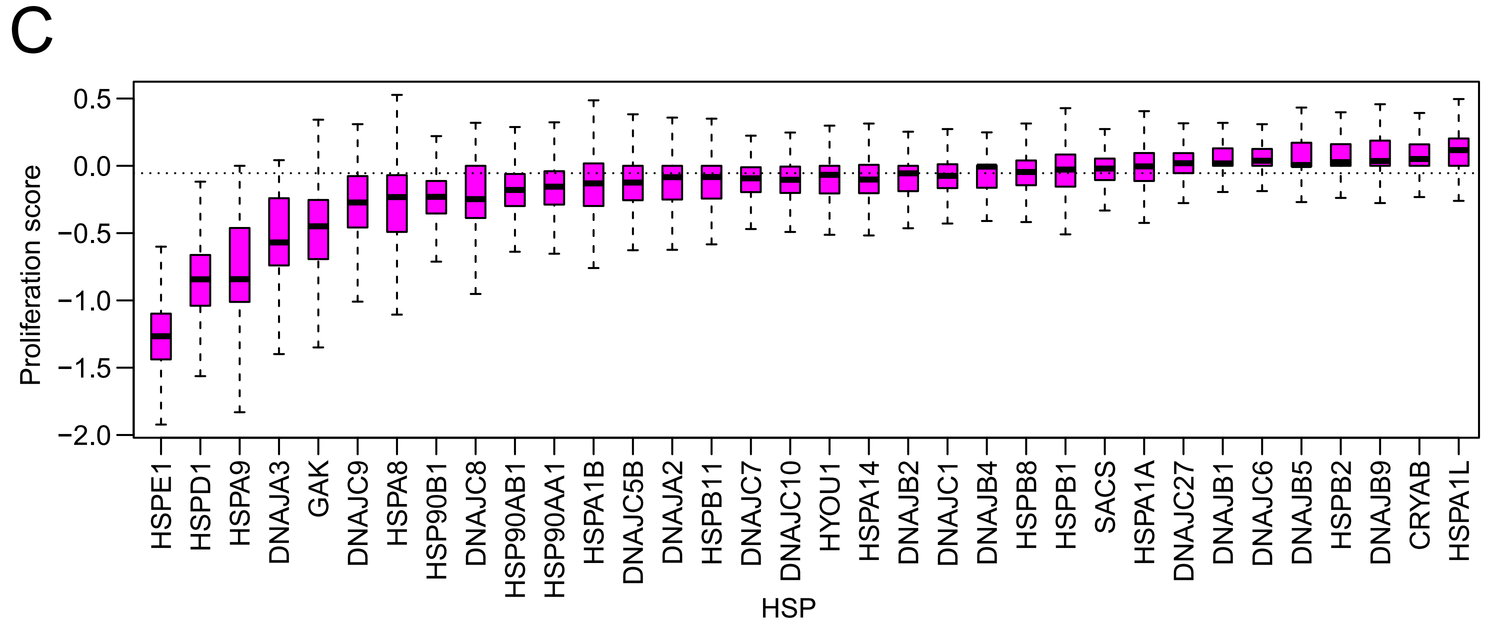

Figure S3. Associations between HSPs and cell proliferation (*ki67*) in cancer cells. A) Associations between HSPs and cell proliferation across cancer lineages. **B)** Proliferation score of HSPs across different cancer lineages. Black line denotes background. **C)** Proliferation score of cancer cell lines upon knockdown of individual HSPs from DRIVE.

Figure S4

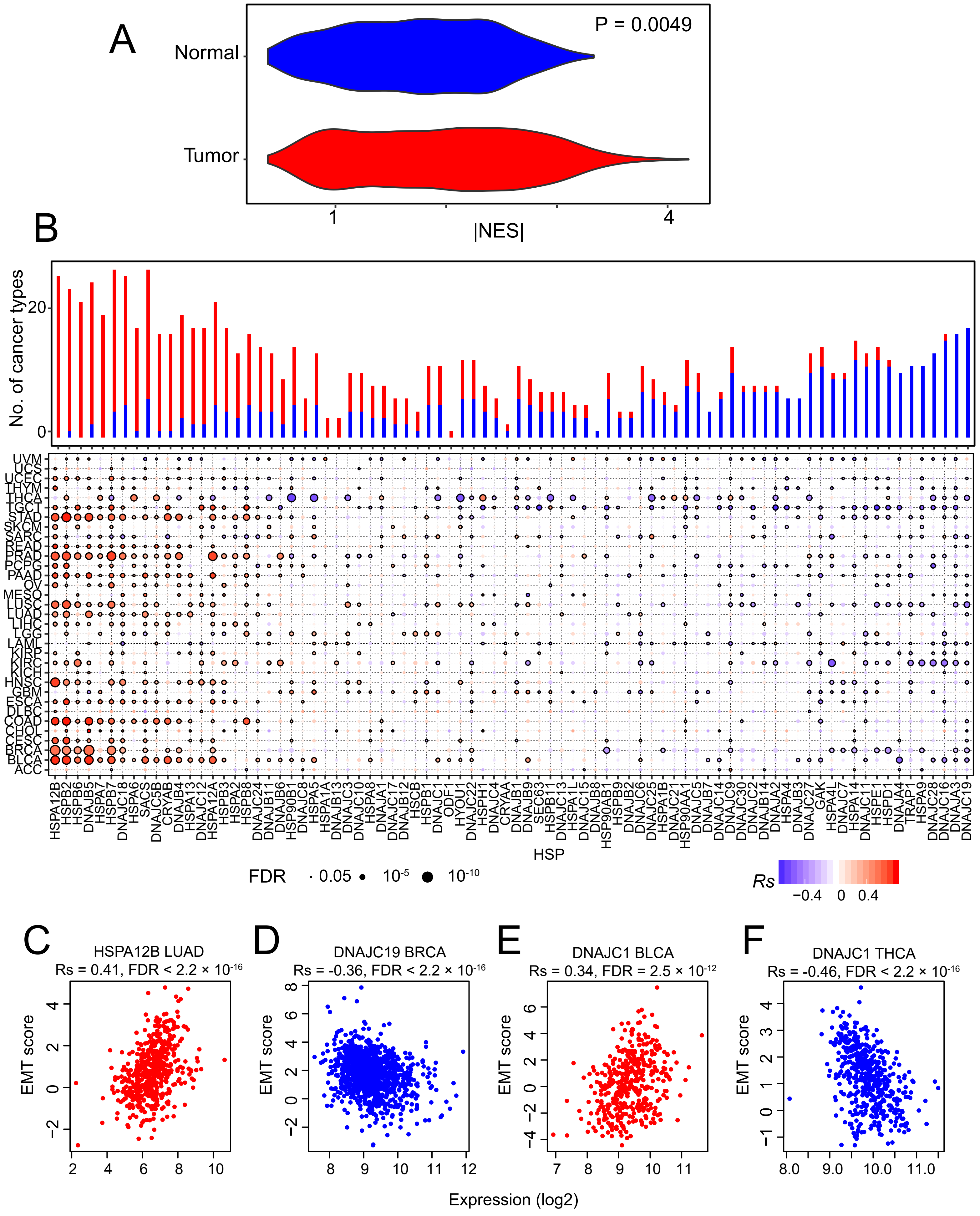
A
P = 0.0049
Normal
Tumor
1
4
|NES|
B
No. of cancer types
20
0
UVM
UCS
UCEC
THYM
THCA
TGCT
STAD
SKCM
SARC
READ
PRAD
PCPG
PAAD
OV
MESO
LUSC
LUAD
LIHC
LGG
LAML
KIRP
KIRC
KICH
HNSC
GBM
ESCA
DLBC
COAD
CHOL
CESC
BRCA
BLCA
ACC
HSP
FDR · 0.05 • 10-5 ● 10-10
Rs −0.4 0 0.4
C
HSPA12B LUAD
Rs = 0.41, FDR < 2.2 × 10-16
D
DNAJC19 BRCA
Rs = -0.36, FDR < 2.2 × 10-16
E
DNAJC1 BLCA
Rs = 0.34, FDR = 2.5 × 10-12
F
DNAJC1 THCA
Rs = -0.46, FDR < 2.2 × 10-16
EMT score
Expression (log2)

Figure S4. Correlation between HSPs and EMT score. A) Comparison of enrichment score between tumor and normal samples. **B)** Correlation of HSPs and EMT score across cancer types. Red and blue denote positive and negative correlation. Histogram height denotes number of cancer types that have significant correlation between HSPs and EMT score. **C-F)** Instances of positive correlation for HSPs (C, E) and of negative correlation for HSPs (D, F) with EMT score.

Figure S5

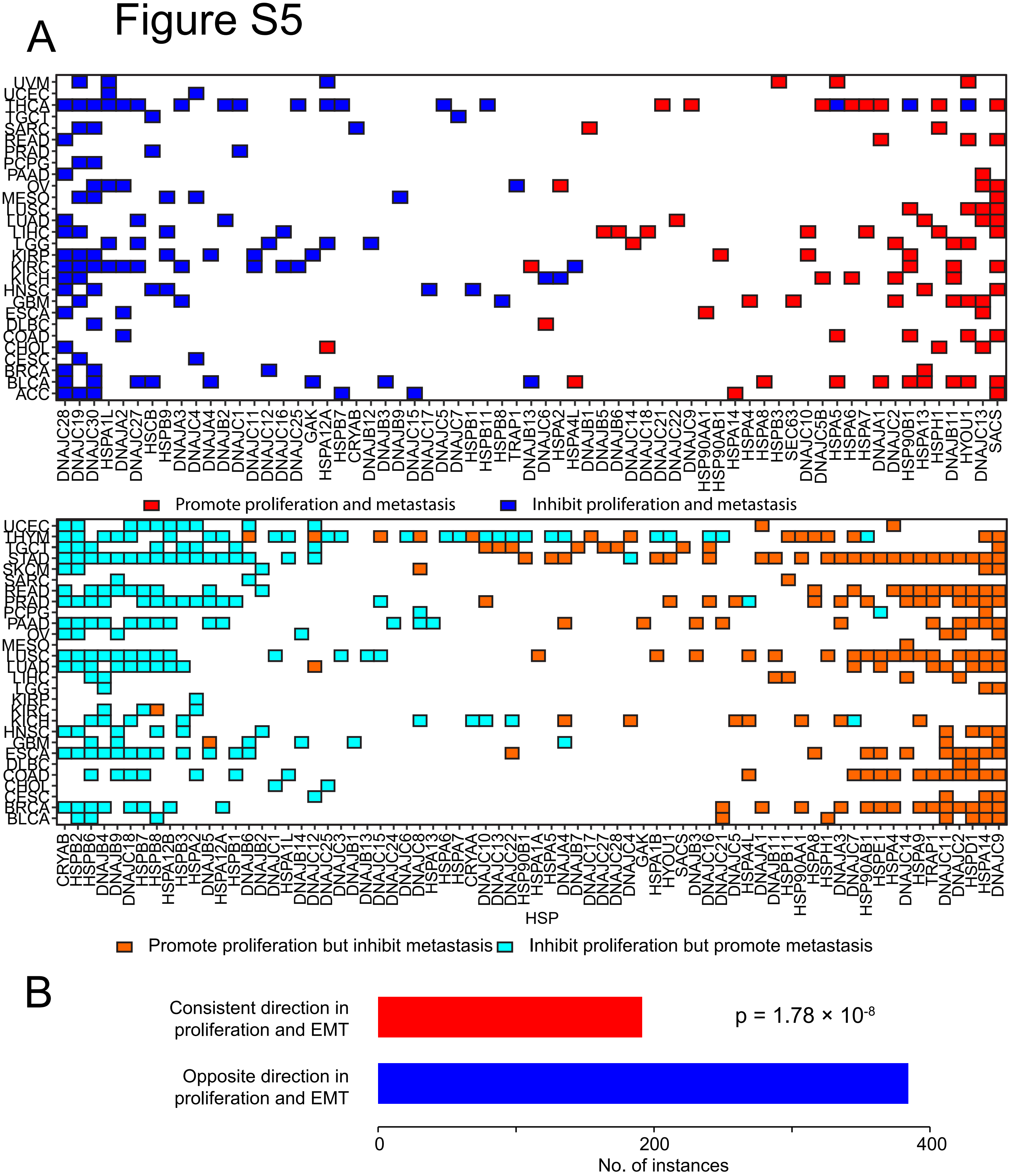

Figure S5. Dual functional effects of HSPs on proliferation and EMT. A) HSPs with consistent directions of effects (upper panel) and opposite directions (bottom panel) for cell proliferation and metastasis. **B)** Number of instances that HSPs have consistent directions of effects in comparison with HSPs that have opposite directions for proliferation and metastasis.

Figure S6

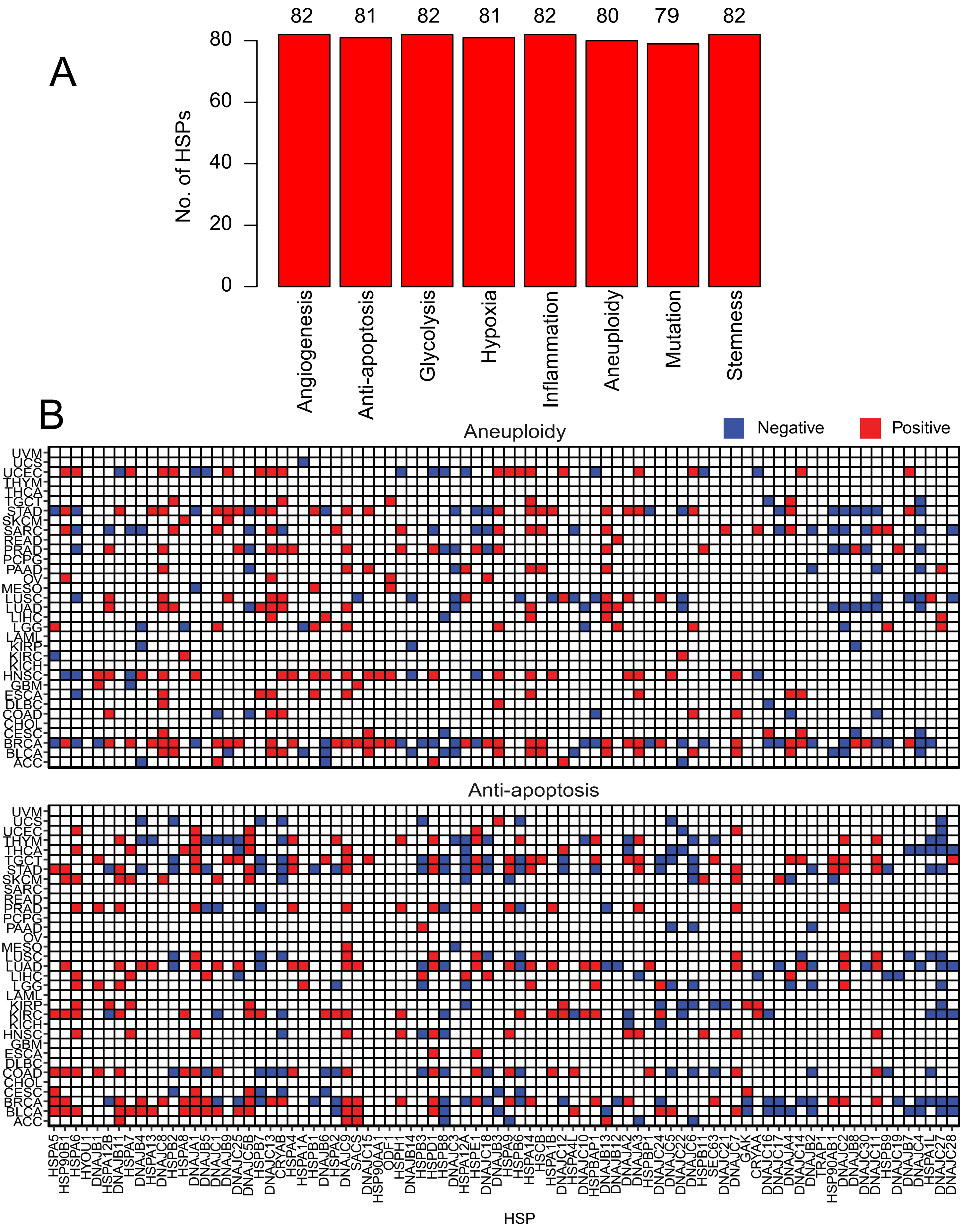

A
82
81
82
81
82
80
79
82
No. of HSPs
0
20
40
60
80
Angiogenesis
Anti-apoptosis
Glycolysis
Hypoxia
Inflammation
Aneuploidy
Mutation
Stemness
B
Aneuploidy
Negative
Positive
Anti-apoptosis
UVM
UCS
UCEC
THYM
THCA
TGCT
STAD
SKCM
SARC
READ
PRAD
PCPG
PAAD
OV
MESO
LUSC
LUAD
LIHC
LGG
LAML
KIRP
KIRC
KICH
HNSC
GBM
ESCA
DLBC
COAD
CHOL
CESC
BRCA
BLCA
ACC
HSPA5
HSP90B1
HSPA6
HYOU1
DNAJB1
HSPA12B
DNAJB11
HSPA7
DNAJB4
HSPA13
DNAJC8
HSPB2
HSPA8
DNAJA1
DNAJB5
DNAJC1
DNAJB9
DNAJC25
DNAJC5B
HSPB7
DNAJC13
CRYAB
HSPA4
HSPA1A
HSPB1
DNAJB6
HSPA2
DNAJC9
SACS
DNAJC15
HSP90AA1
ODF1
HSPH1
DNAJB14
HSPB3
HSPD1
HSPB8
DNAJC3
HSPA12A
HSPE1
DNAJC18
DNAJB3
HSPA9
HSPB6
HSPA14
HSCB
HSPA1B
DNAJC12
HSPA4L
DNAJC10
HSPBAP1
DNAJB13
DNAJB12
DNAJA2
DNAJA3
HSPBP1
DNAJC24
DNAJC5
DNAJC22
DNAJC6
HSPB11
SEC63
DNAJC21
DNAJC7
GAK
CRYAA
DNAJC16
DNAJC17
DNAJA4
DNAJC14
DNAJB2
TRAP1
HSP90AB1
DNAJC2
DNAJB8
DNAJC30
DNAJC11
HSPB9
DNAJC19
DNAJB7
DNAJC4
HSPA1L
DNAJC27
DNAJC28
HSP

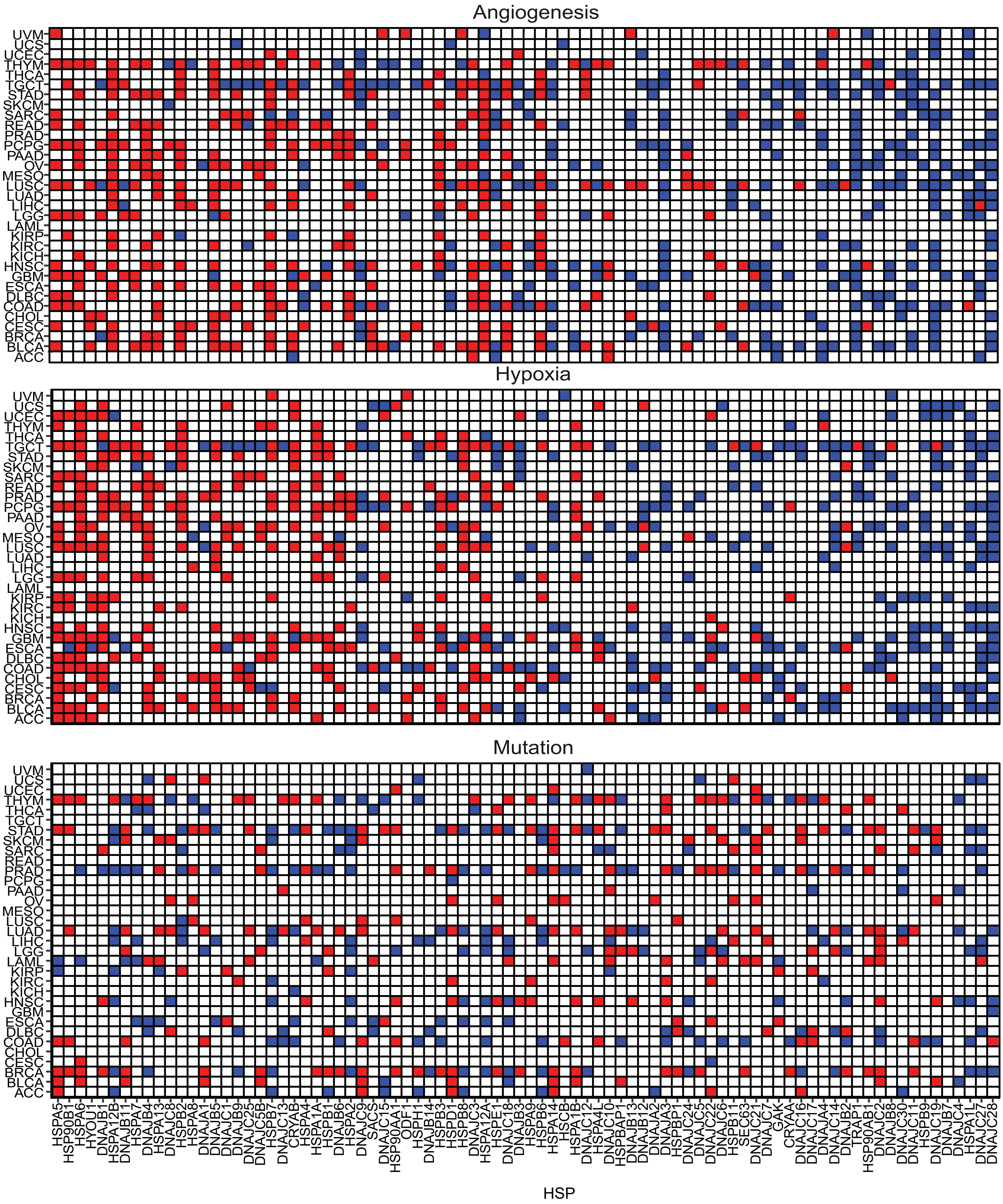
Angiogenesis
Hypoxia
Mutation
UVM
UCS
UCEC
THYM
THCA
TGCT
STAD
SKCM
SARC
READ
PRAD
PCPG
PAAD
OV
MESO
LUSC
LUAD
LIHC
LGG
LAML
KIRP
KIRC
KICH
HNSC
GBM
ESCA
DLBC
COAD
CHOL
CESC
BRCA
BLCA
ACC
HSPA5
HSP90B1
HSPA6
HYOU1
DNAJB1
HSPA12B
DNAJB11
HSPA7
DNAJB4
HSPA13
DNAJC8
HSPB2
HSPA8
DNAJA1
DNAJB5
DNAJC1
DNAJB9
DNAJC25
DNAJC5B
HSPB7
DNAJC13
CRYAB
HSPA4
HSPA1A
HSPB1
DNAJB6
HSPA2
DNAJC9
SACS
DNAJC15
HSP90AA1
ODF1
HSPH1
DNAJB14
HSPB3
HSPD1
HSPB8
DNAJC3
HSPA12A
HSPE1
DNAJC18
DNAJB3
HSPA9
HSPB6
HSPA14
HSCB
HSPA1B
DNAJC12
HSPA4L
DNAJC10
HSPBAP1
DNAJB13
DNAJB12
DNAJA2
DNAJA3
HSPBP1
DNAJC24
DNAJC5
DNAJC22
DNAJC6
HSPB11
SEC63
DNAJC21
DNAJC7
GAK
CRYAA
DNAJC16
DNAJC17
DNAJA4
DNAJC14
DNAJB2
TRAP1
HSP90AB1
DNAJC2
DNAJB8
DNAJC30
DNAJC11
HSPB9
DNAJC19
DNAJB7
DNAJC4
HSPA1L
DNAJC27
DNAJC28
HSP

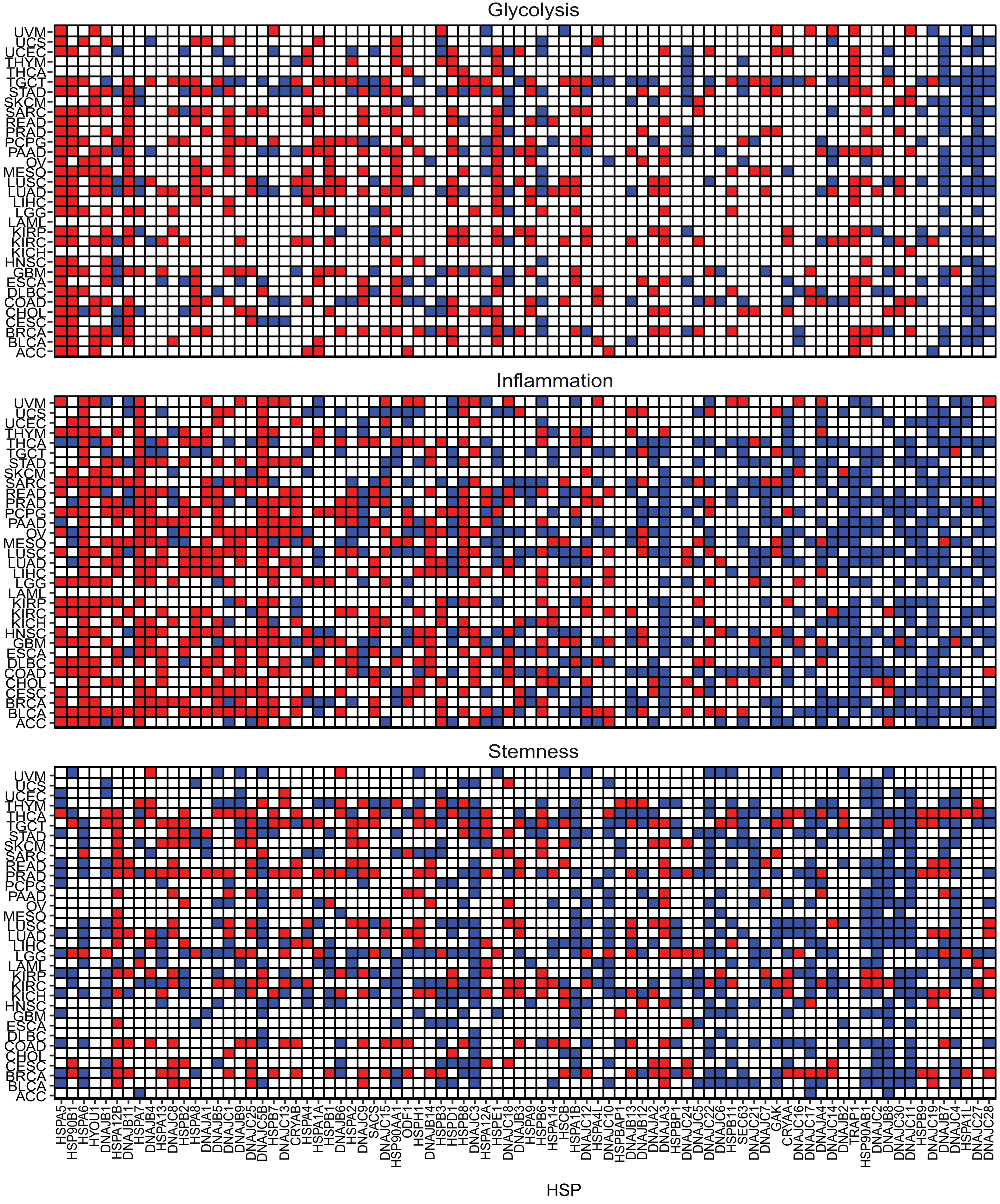
Glycolysis
Inflammation
Stemness
UVM
UCS
UCEC
THYM
THCA
TGCT
STAD
SKCM
SARC
READ
PRAD
PCPG
PAAD
OV
MESO
LUSC
LUAD
LIHC
LGG
LAML
KIRP
KIRC
KICH
HNSC
GBM
ESCA
DLBC
COAD
CHOL
CESC
BRCA
BLCA
ACC
HSPA5
HSP90B1
HSPA6
HYOU1
DNAJB1
HSPA12B
DNAJB11
HSPA7
DNAJB4
HSPA13
DNAJC8
HSPB2
HSPA8
DNAJA1
DNAJB5
DNAJC1
DNAJB9
DNAJC25
DNAJC5B
HSPB7
DNAJC13
CRYAB
HSPA4
HSPA1A
HSPB1
DNAJB6
HSPA2
DNAJC9
SACS
DNAJC15
HSP90AA1
ODF1
HSPH1
DNAJB14
HSPB3
HSPD1
HSPB8
DNAJC3
HSPA12A
HSPE1
DNAJC18
DNAJB3
HSPA9
HSPB6
HSPA14
HSCB
HSPA1B
DNAJC12
HSPA4L
DNAJC10
HSPBAP1
DNAJB13
DNAJB12
DNAJA2
DNAJA3
HSPBP1
DNAJC24
DNAJC5
DNAJC22
DNAJC6
HSPB11
SEC63
DNAJC21
DNAJC7
GAK
CRYAA
DNAJC16
DNAJC17
DNAJC14
DNAJB2
TRAP1
HSP90AB1
DNAJC2
DNAJB8
DNAJC30
DNAJC11
HSPB9
DNAJC19
DNAJB7
DNAJC4
HSPA1L
DNAJC27
DNAJC28
HSP

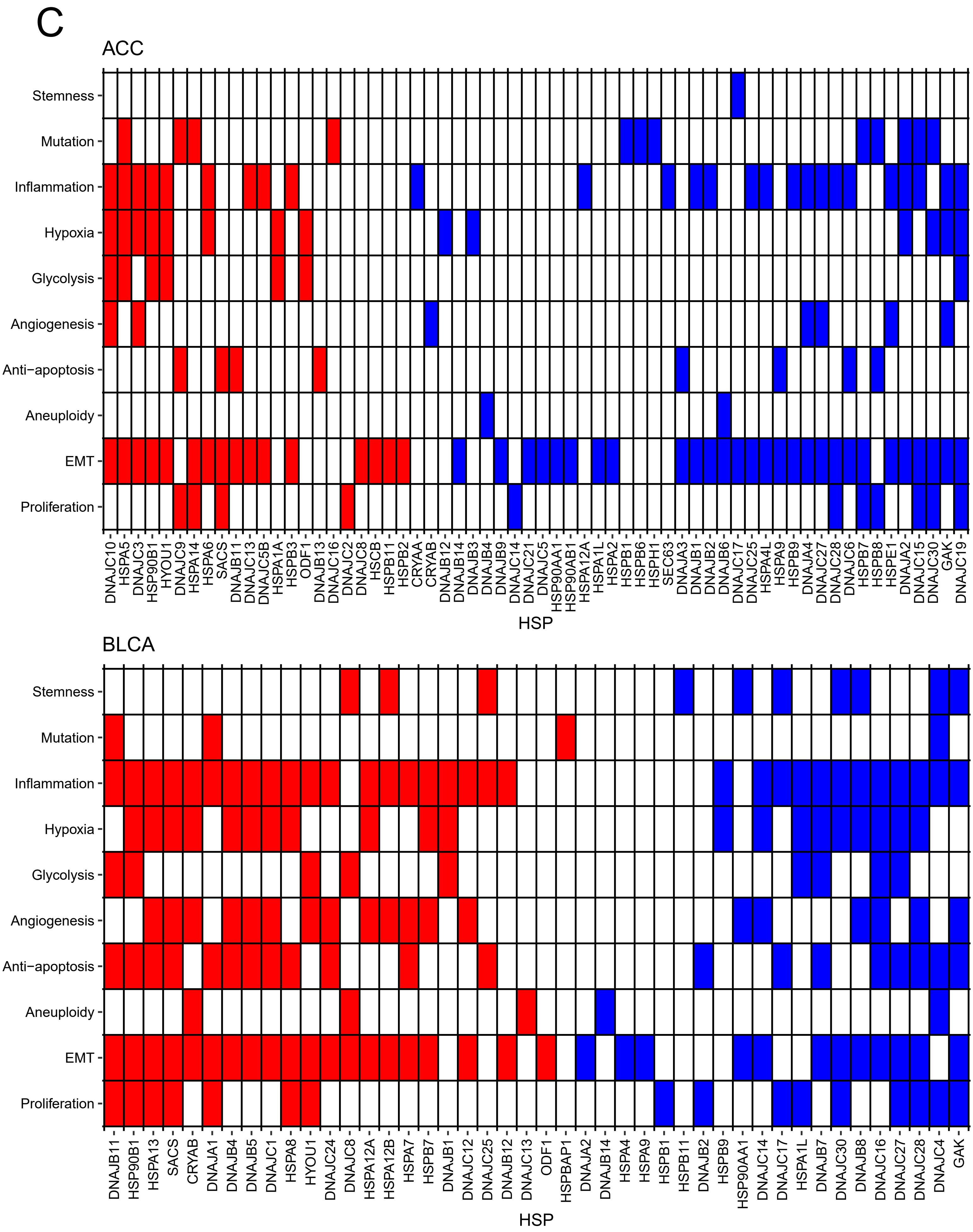
C
ACC
Stemness
Mutation
Inflammation
Hypoxia
Glycolysis
Angiogenesis
Anti−apoptosis
Aneuploidy
EMT
Proliferation
DNAJC10 HSPA5 DNAJC3 HSP90B1 HYOU1 DNAJC9 HSPA14 HSPA6 SACS DNAJB11 DNAJC13 DNAJC5B HSPA1A HSPB3 ODF1 DNAJB13 DNAJC16 DNAJC2 DNAJC8 HSCB HSPB11 HSPB2 CRYAA CRYAB DNAJB12 DNAJB14 DNAJB3 DNAJB4 DNAJB9 DNAJC14 DNAJC21 DNAJC5 HSP90AA1 HSP90AB1 HSPA12A HSPA1L HSPA2 HSPB1 HSPB6 HSPH1 SEC63 DNAJA3 DNAJB1 DNAJB2 DNAJB6 DNAJC17 DNAJC25 HSPA4L HSPA9 HSPB9 DNAJA4 DNAJC27 DNAJC28 DNAJC6 HSPB7 HSPB8 HSPE1 DNAJA2 DNAJC15 DNAJC30 GAK DNAJC19
HSP
BLCA
Stemness
Mutation
Inflammation
Hypoxia
Glycolysis
Angiogenesis
Anti−apoptosis
Aneuploidy
EMT
Proliferation
DNAJB11 HSP90B1 HSPA13 SACS CRYAB DNAJA1 DNAJB4 DNAJB5 DNAJC1 HSPA8 HYOU1 DNAJC24 DNAJC8 HSPA12A HSPA12B HSPA7 HSPB7 DNAJB1 DNAJC12 DNAJC25 DNAJB12 DNAJC13 ODF1 HSPBAP1 DNAJA2 DNAJB14 HSPA4 HSPA9 HSPB1 HSPB11 DNAJB2 HSPB9 HSP90AA1 DNAJC14 DNAJC17 HSPA1L DNAJB7 DNAJC30 DNAJB8 DNAJC16 DNAJC27 DNAJC28 DNAJC4 GAK
HSP

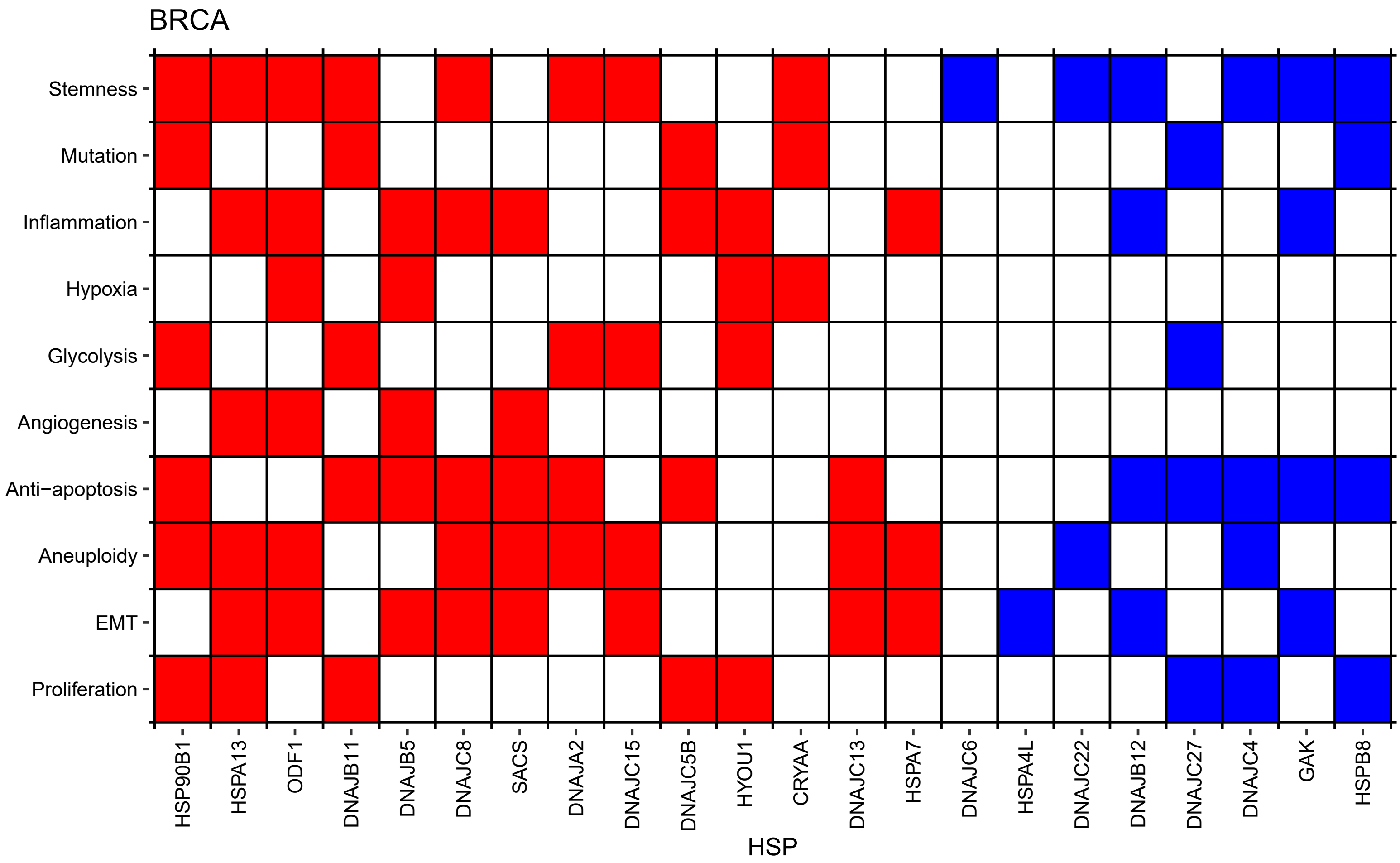
BRCA
Stemness
Mutation
Inflammation
Hypoxia
Glycolysis
Angiogenesis
Anti−apoptosis
Aneuploidy
EMT
Proliferation
HSP90B1
HSPA13
ODF1
DNAJB11
DNAJB5
DNAJC8
SACS
DNAJA2
DNAJC15
DNAJC5B
HYOU1
CRYAA
DNAJC13
HSPA7
DNAJC6
HSPA4L
DNAJC22
DNAJB12
DNAJC27
DNAJC4
GAK
HSPB8
HSP

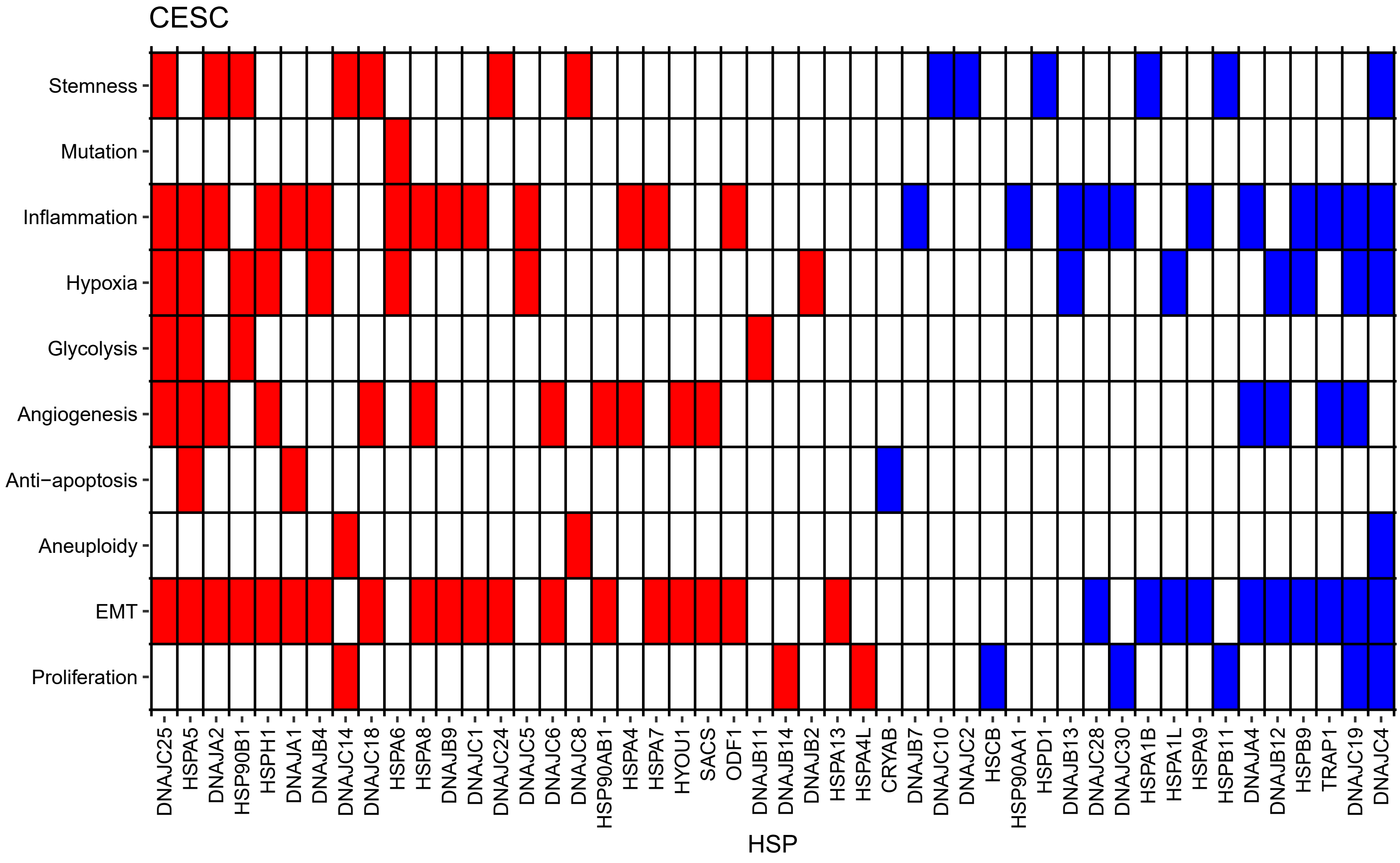
CESC
Stemness
Mutation
Inflammation
Hypoxia
Glycolysis
Angiogenesis
Anti−apoptosis
Aneuploidy
EMT
Proliferation
DNAJC25
HSPA5
DNAJA2
HSP90B1
HSPH1
DNAJA1
DNAJB4
DNAJC14
DNAJC18
HSPA6
HSPA8
DNAJB9
DNAJC1
DNAJC24
DNAJC5
DNAJC6
DNAJC8
HSP90AB1
HSPA4
HSPA7
HYOU1
SACS
ODF1
DNAJB11
DNAJB14
DNAJB2
HSPA13
HSPA4L
CRYAB
DNAJB7
DNAJC10
DNAJC2
HSCB
HSP90AA1
HSPD1
DNAJB13
DNAJC28
DNAJC30
HSPA1B
HSPA1L
HSPA9
HSPB11
DNAJA4
DNAJB12
HSPB9
TRAP1
DNAJC19
DNAJC4
HSP

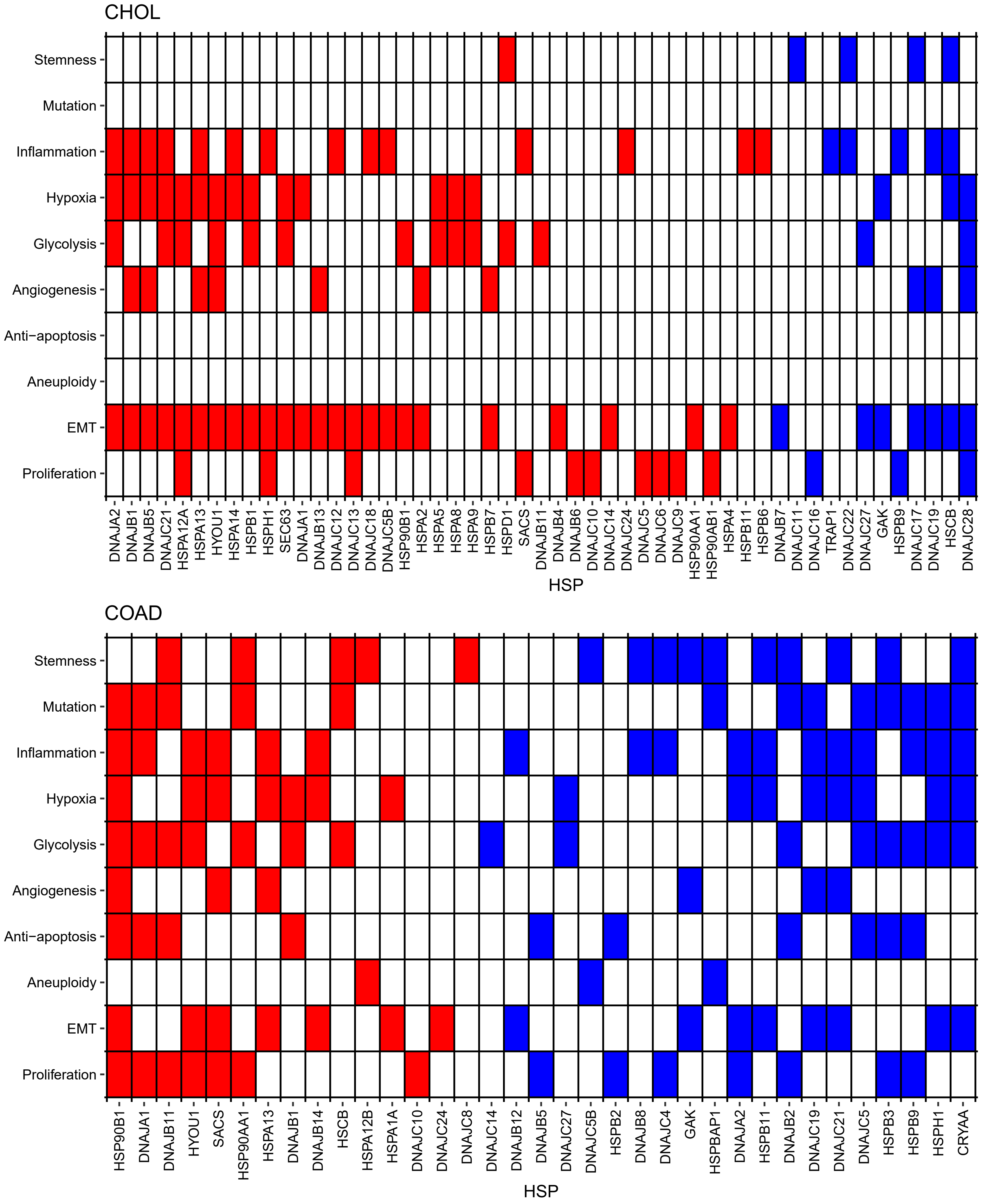
CHOL
Stemness
Mutation
Inflammation
Hypoxia
Glycolysis
Angiogenesis
Anti−apoptosis
Aneuploidy
EMT
Proliferation
DNAJA2 DNAJB1 DNAJB5 DNAJC21 HSPA12A HSPA13 HYOU1 HSPA14 HSPB1 HSPH1 SEC63 DNAJA1 DNAJB13 DNAJC12 DNAJC13 DNAJC18 DNAJC5B HSP90B1 HSPA2 HSPA5 HSPA8 HSPA9 HSPB7 HSPD1 SACS DNAJB11 DNAJB4 DNAJB6 DNAJC10 DNAJC14 DNAJC24 DNAJC5 DNAJC6 DNAJC9 HSP90AA1 HSP90AB1 HSPA4 HSPB11 HSPB6 DNAJB7 DNAJC11 DNAJC16 TRAP1 DNAJC22 DNAJC27 GAK HSPB9 DNAJC17 DNAJC19 HSCB DNAJC28
HSP
COAD
Stemness
Mutation
Inflammation
Hypoxia
Glycolysis
Angiogenesis
Anti−apoptosis
Aneuploidy
EMT
Proliferation
HSP90B1 DNAJA1 DNAJB11 HYOU1 SACS HSP90AA1 HSPA13 DNAJB1 DNAJB14 HSCB HSPA12B HSPA1A DNAJC10 DNAJC24 DNAJC8 DNAJC14 DNAJB12 DNAJB5 DNAJC27 DNAJC5B HSPB2 DNAJB8 DNAJC4 GAK HSPBAP1 DNAJA2 HSPB11 DNAJB2 DNAJC19 DNAJC21 DNAJC5 HSPB3 HSPB9 HSPH1 CRYAA
HSP

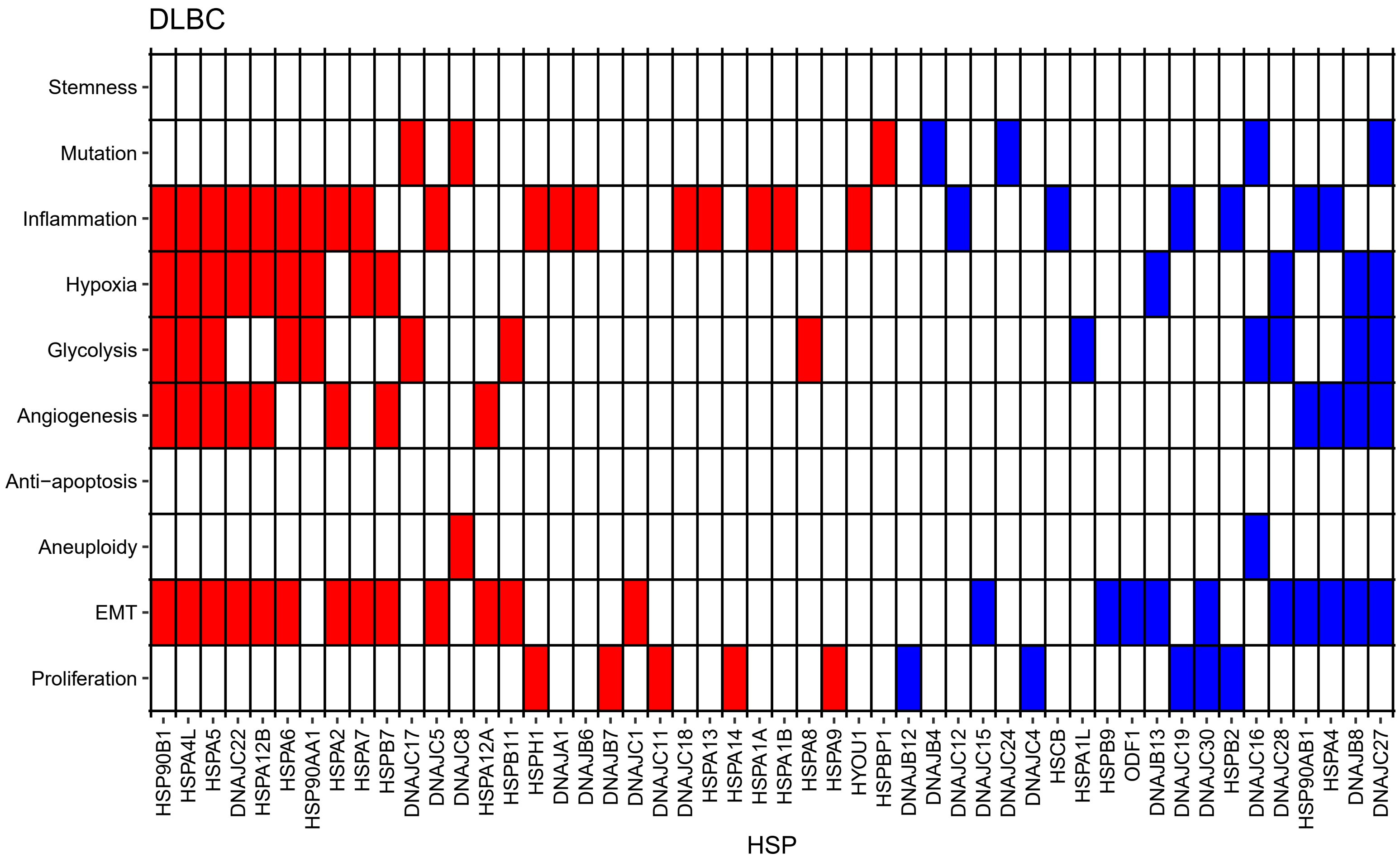
DLBC
Stemness
Mutation
Inflammation
Hypoxia
Glycolysis
Angiogenesis
Anti−apoptosis
Aneuploidy
EMT
Proliferation
HSP90B1
HSPA4L
HSPA5
DNAJC22
HSPA12B
HSPA6
HSP90AA1
HSPA2
HSPA7
HSPB7
DNAJC17
DNAJC5
DNAJC8
HSPA12A
HSPB11
HSPH1
DNAJA1
DNAJB6
DNAJB7
DNAJC1
DNAJC11
DNAJC18
HSPA13
HSPA14
HSPA1A
HSPA1B
HSPA8
HSPA9
HYOU1
HSPBP1
DNAJB12
DNAJB4
DNAJC12
DNAJC15
DNAJC24
DNAJC4
HSCB
HSPA1L
HSPB9
ODF1
DNAJB13
DNAJC19
DNAJC30
HSPB2
DNAJC16
DNAJC28
HSP90AB1
HSPA4
DNAJB8
DNAJC27
HSP

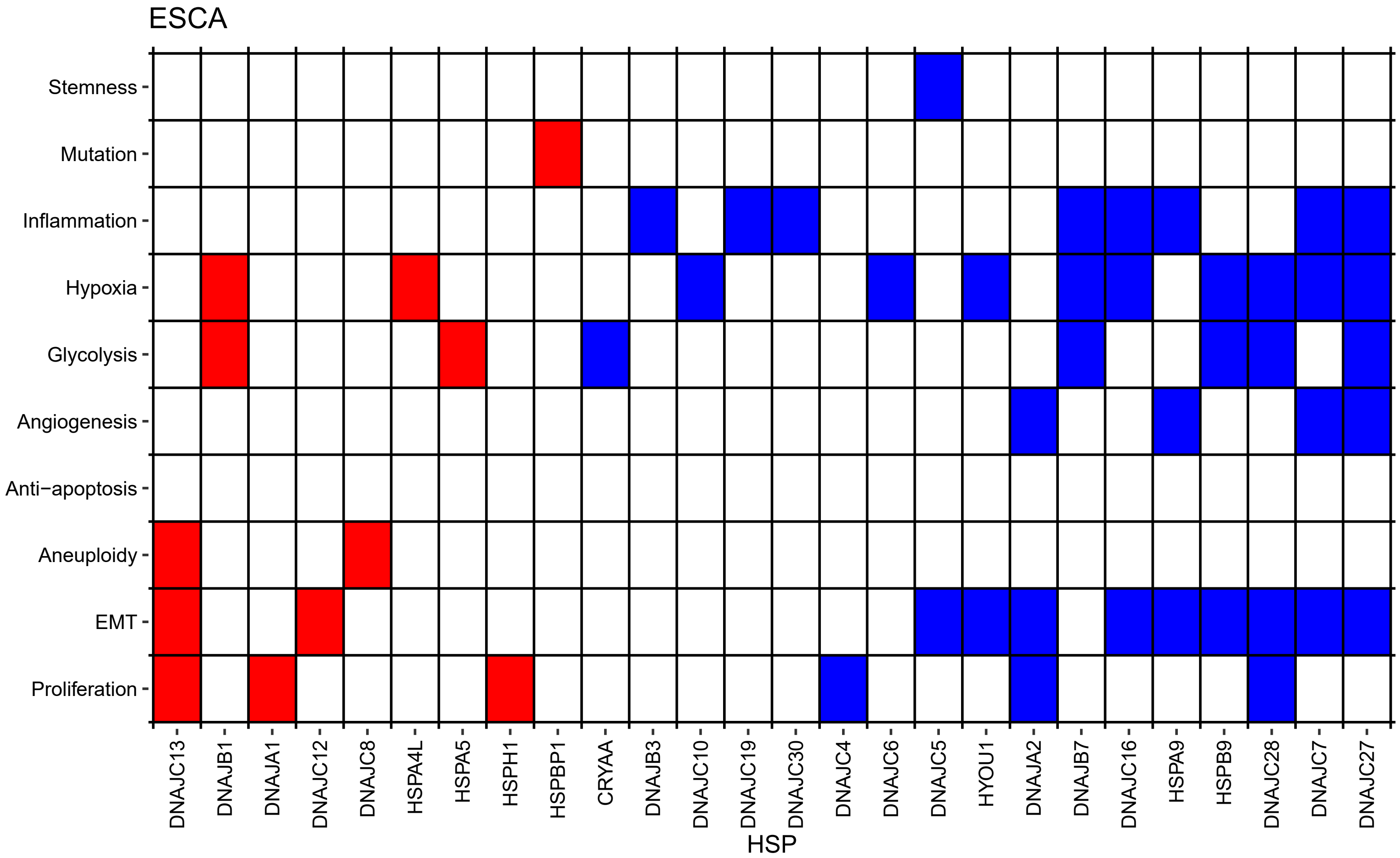
ESCA
Stemness
Mutation
Inflammation
Hypoxia
Glycolysis
Angiogenesis
Anti−apoptosis
Aneuploidy
EMT
Proliferation
DNAJC13
DNAJB1
DNAJA1
DNAJC12
DNAJC8
HSPA4L
HSPA5
HSPH1
HSPBP1
CRYAA
DNAJB3
DNAJC10
DNAJC19
DNAJC30
DNAJC4
DNAJC6
DNAJC5
HYOU1
DNAJA2
DNAJB7
DNAJC16
HSPA9
HSPB9
DNAJC28
DNAJC7
DNAJC27
HSP

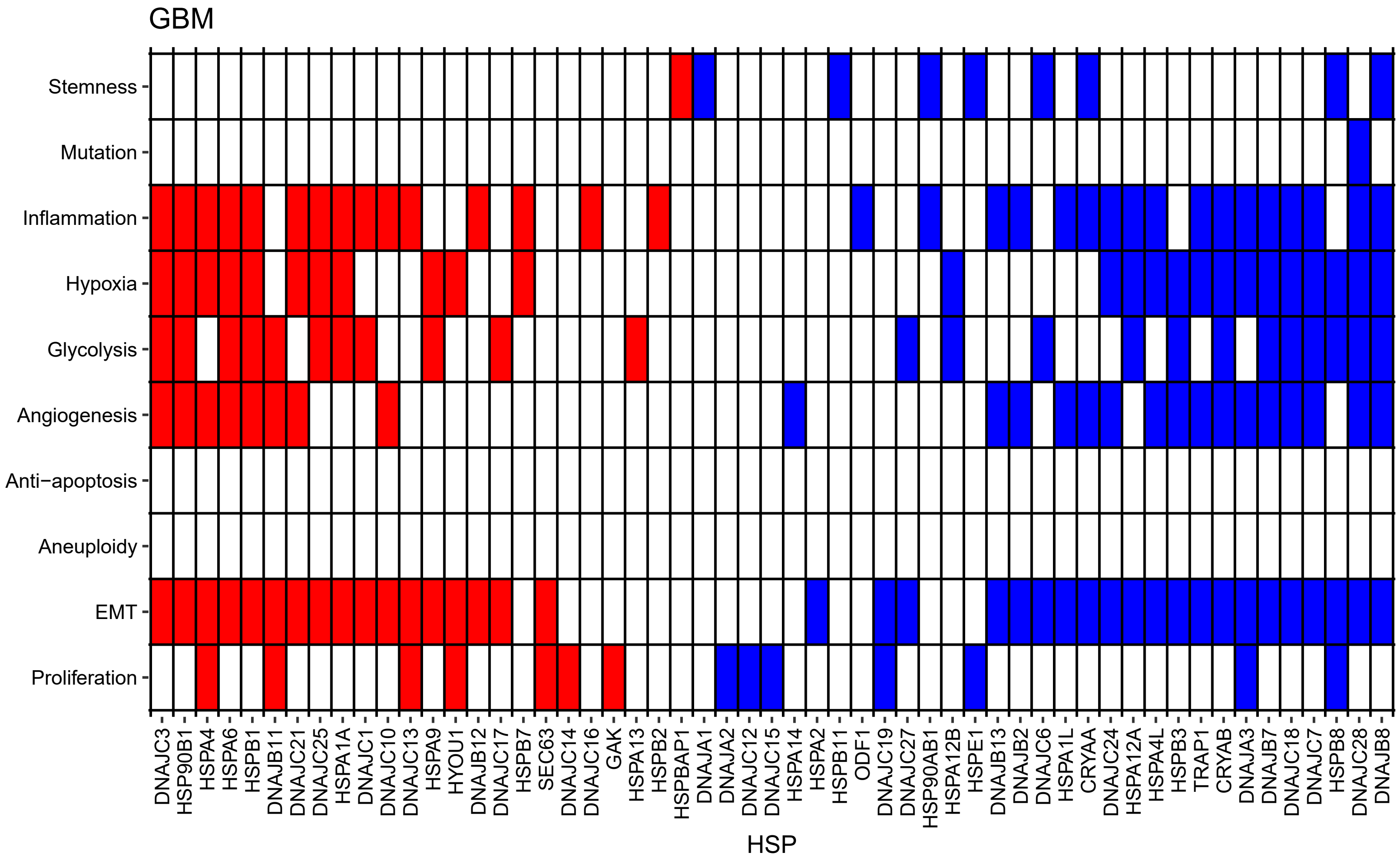
GBM
Stemness
Mutation
Inflammation
Hypoxia
Glycolysis
Angiogenesis
Anti−apoptosis
Aneuploidy
EMT
Proliferation
DNAJC3 HSP90B1 HSPA4 HSPA6 HSPB1 DNAJB11 DNAJC21 DNAJC25 HSPA1A DNAJC1 DNAJC10 DNAJC13 HSPA9 HYOU1 DNAJB12 DNAJC17 HSPB7 SEC63 DNAJC14 DNAJC16 GAK HSPA13 HSPB2 HSPBAP1 DNAJA1 DNAJA2 DNAJC12 DNAJC15 HSPA14 HSPA2 HSPB11 ODF1 DNAJC19 DNAJC27 HSP90AB1 HSPA12B HSPE1 DNAJB13 DNAJB2 DNAJC6 HSPA1L CRYAA DNAJC24 HSPA12A HSPA4L HSPB3 TRAP1 CRYAB DNAJA3 DNAJB7 DNAJC18 DNAJC7 HSPB8 DNAJC28 DNAJB8
HSP

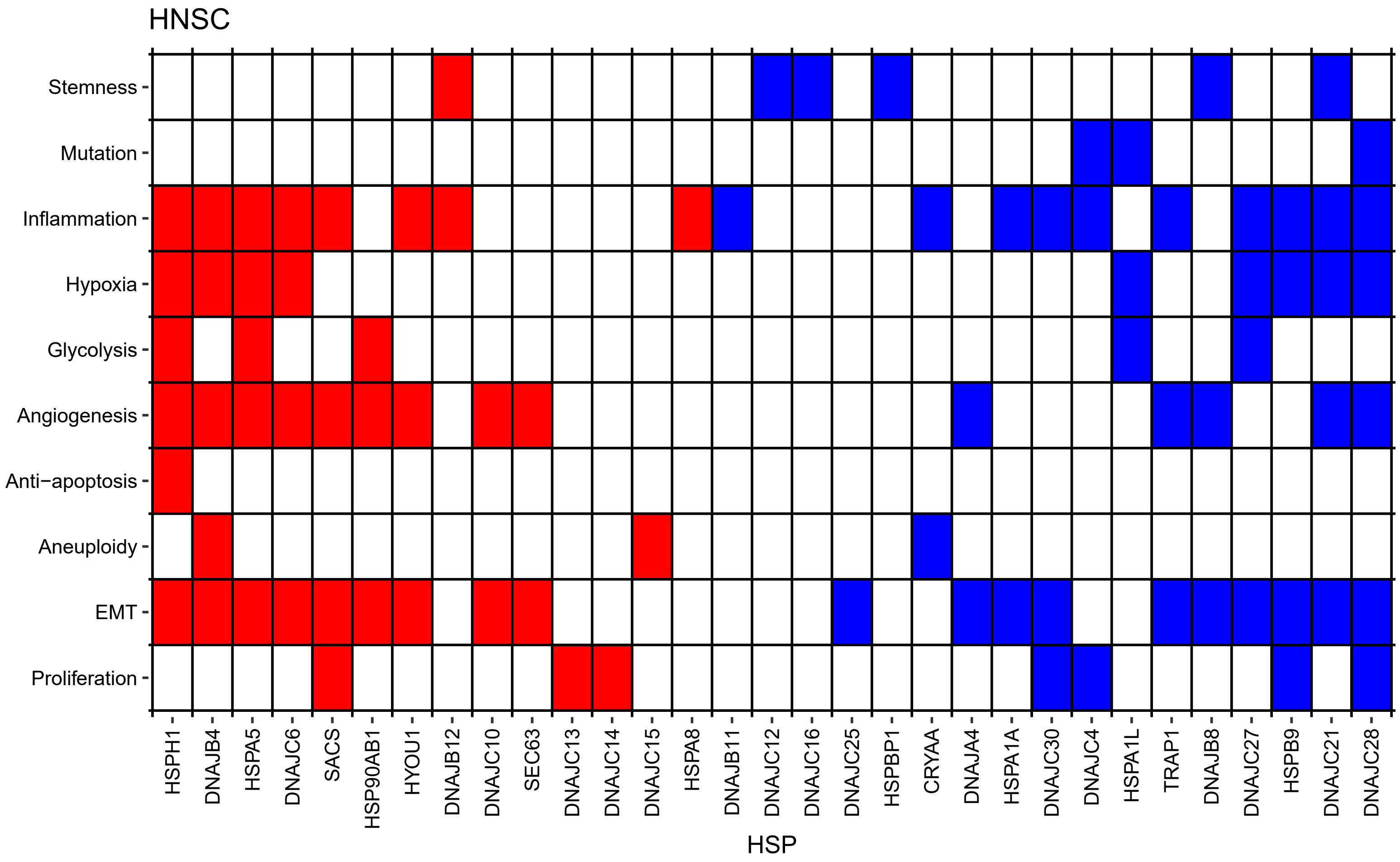
HNSC
Stemness
Mutation
Inflammation
Hypoxia
Glycolysis
Angiogenesis
Anti−apoptosis
Aneuploidy
EMT
Proliferation
HSPH1 DNAJB4 HSPA5 DNAJC6 SACS HSP90AB1 HYOU1 DNAJB12 DNAJC10 SEC63 DNAJC13 DNAJC14 DNAJC15 HSPA8 DNAJB11 DNAJC12 DNAJC16 DNAJC25 HSPBP1 CRYAA DNAJA4 HSPA1A DNAJC30 DNAJC4 HSPA1L TRAP1 DNAJB8 DNAJC27 HSPB9 DNAJC21 DNAJC28
HSP

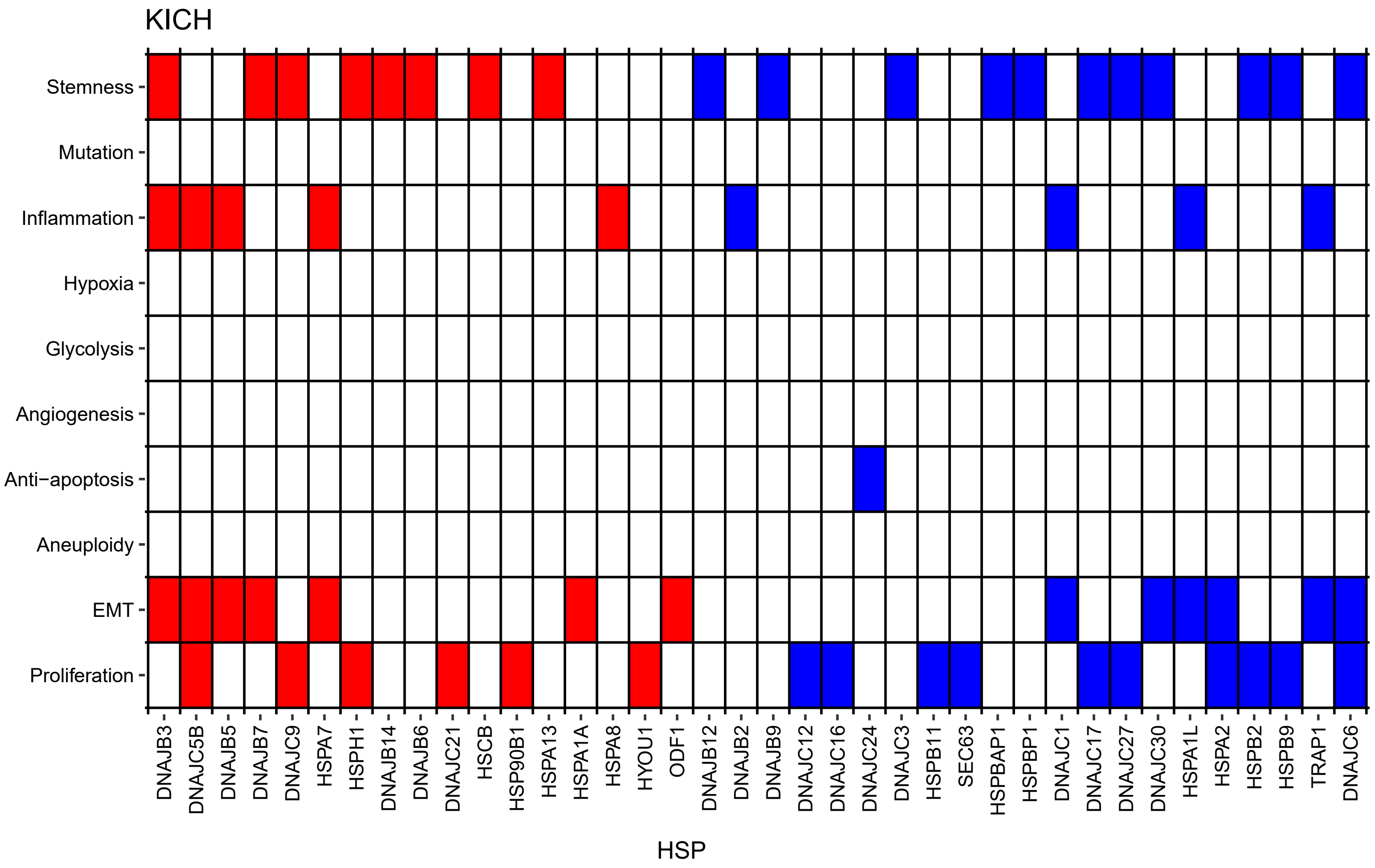

KICH
Stemness
Mutation
Inflammation
Hypoxia
Glycolysis
Angiogenesis
Anti−apoptosis
Aneuploidy
EMT
Proliferation
DNAJB3
DNAJC5B
DNAJB5
DNAJB7
DNAJC9
HSPA7
HSPH1
DNAJB14
DNAJB6
DNAJC21
HSCB
HSP90B1
HSPA13
HSPA1A
HSPA8
HYOU1
ODF1
DNAJB12
DNAJB2
DNAJB9
DNAJC12
DNAJC16
DNAJC24
DNAJC3
HSPB11
SEC63
HSPBAP1
HSPBP1
DNAJC1
DNAJC17
DNAJC27
DNAJC30
HSPA1L
HSPA2
HSPB2
HSPB9
TRAP1
DNAJC6
HSP

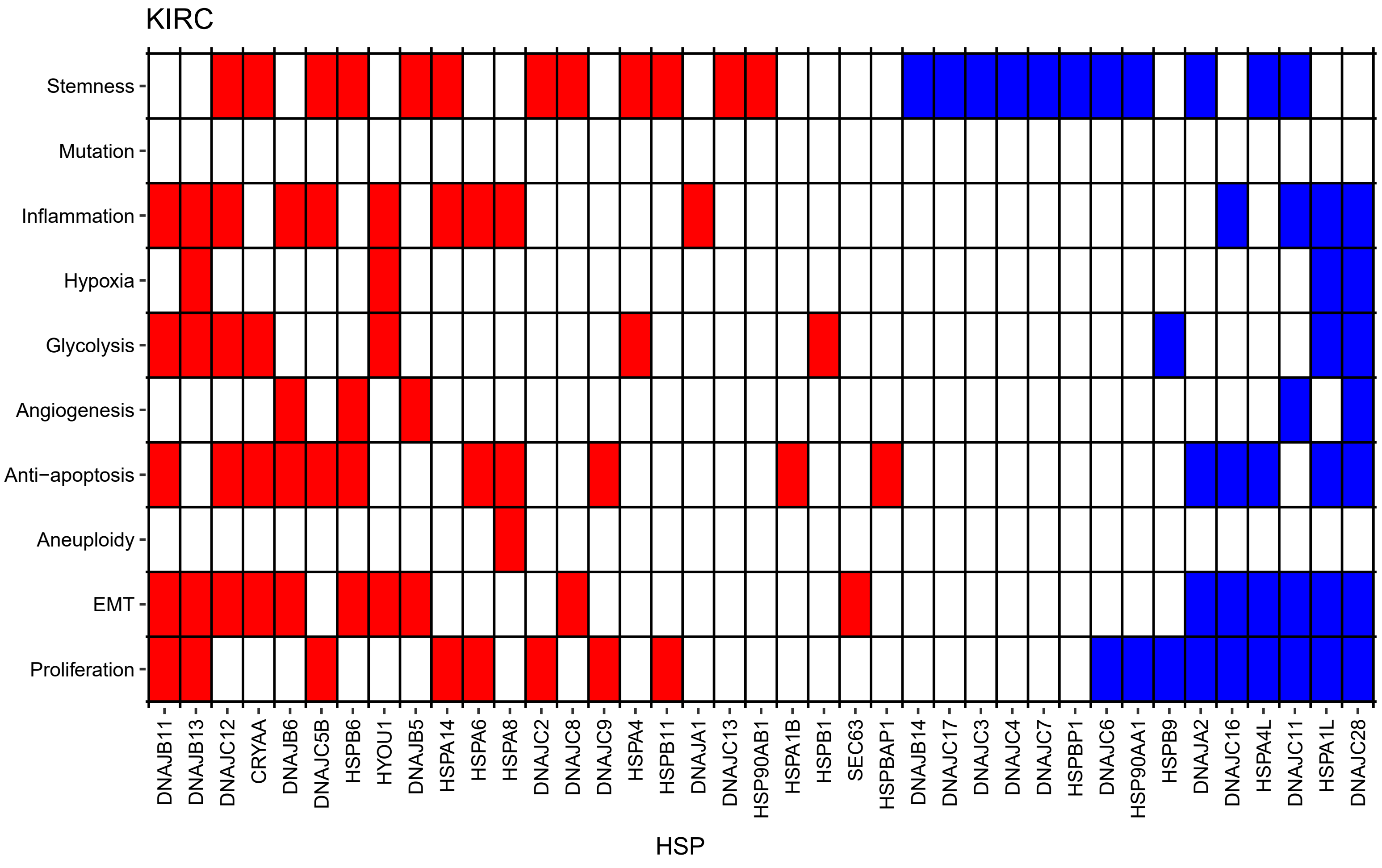

KIRC
Stemness
Mutation
Inflammation
Hypoxia
Glycolysis
Angiogenesis
Anti−apoptosis
Aneuploidy
EMT
Proliferation
DNAJB11
DNAJB13
DNAJC12
CRYAA
DNAJB6
DNAJC5B
HSPB6
HYOU1
DNAJB5
HSPA14
HSPA6
HSPA8
DNAJC2
DNAJC8
DNAJC9
HSPA4
HSPB11
DNAJA1
DNAJC13
HSP90AB1
HSPA1B
HSPB1
SEC63
HSPBAP1
DNAJB14
DNAJC17
DNAJC3
DNAJC4
DNAJC7
HSPBP1
DNAJC6
HSP90AA1
HSPB9
DNAJA2
DNAJC16
HSPA4L
DNAJC11
HSPA1L
DNAJC28
HSP

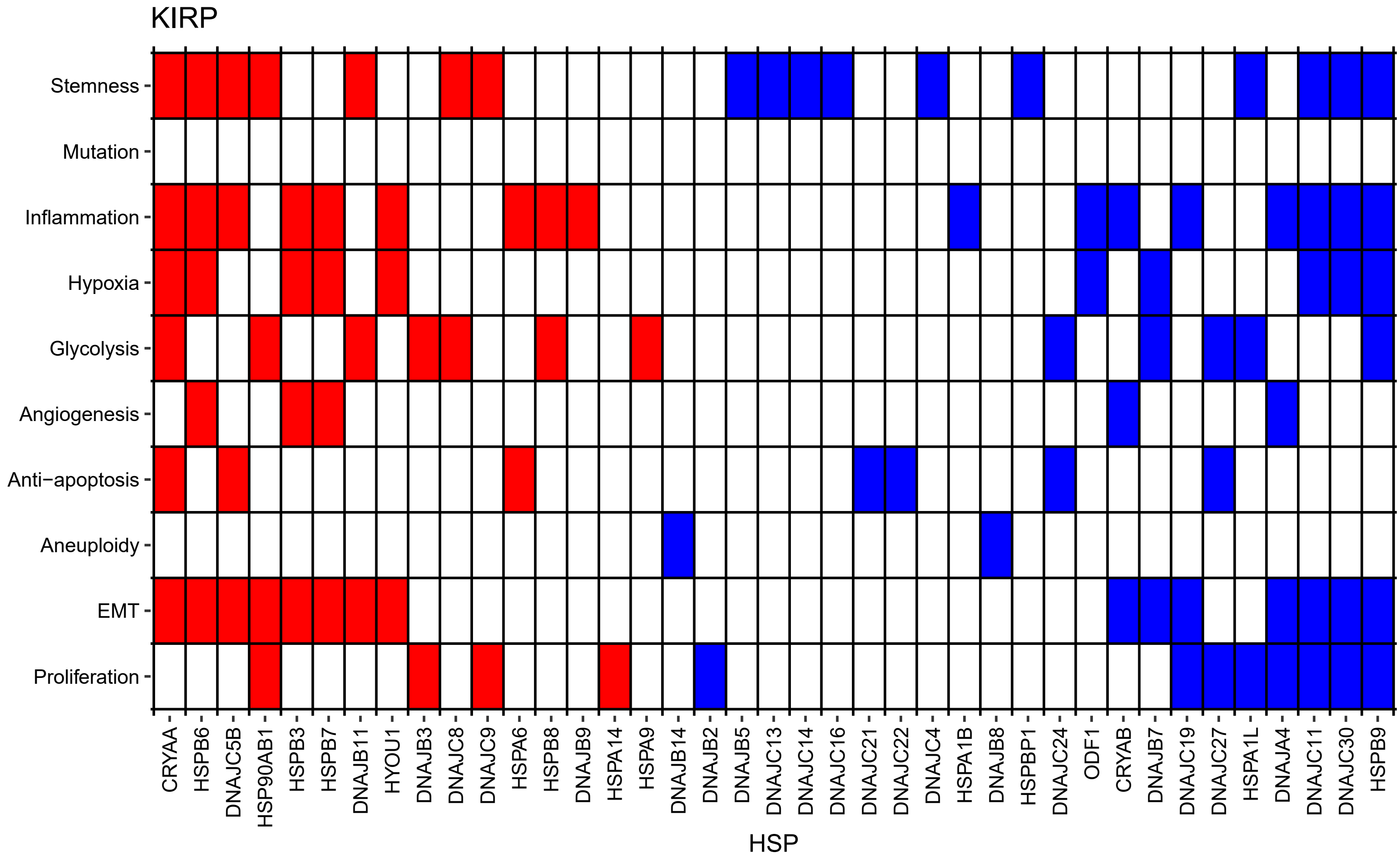
KIRP
Stemness
Mutation
Inflammation
Hypoxia
Glycolysis
Angiogenesis
Anti−apoptosis
Aneuploidy
EMT
Proliferation
CRYAA
HSPB6
DNAJC5B
HSP90AB1
HSPB3
HSPB7
DNAJB11
HYOU1
DNAJB3
DNAJC8
DNAJC9
HSPA6
HSPB8
DNAJB9
HSPA14
HSPA9
DNAJB14
DNAJB2
DNAJB5
DNAJC13
DNAJC14
DNAJC16
DNAJC21
DNAJC22
DNAJC4
HSPA1B
DNAJB8
HSPBP1
DNAJC24
ODF1
CRYAB
DNAJB7
DNAJC19
DNAJC27
HSPA1L
DNAJA4
DNAJC11
DNAJC30
HSPB9
HSP

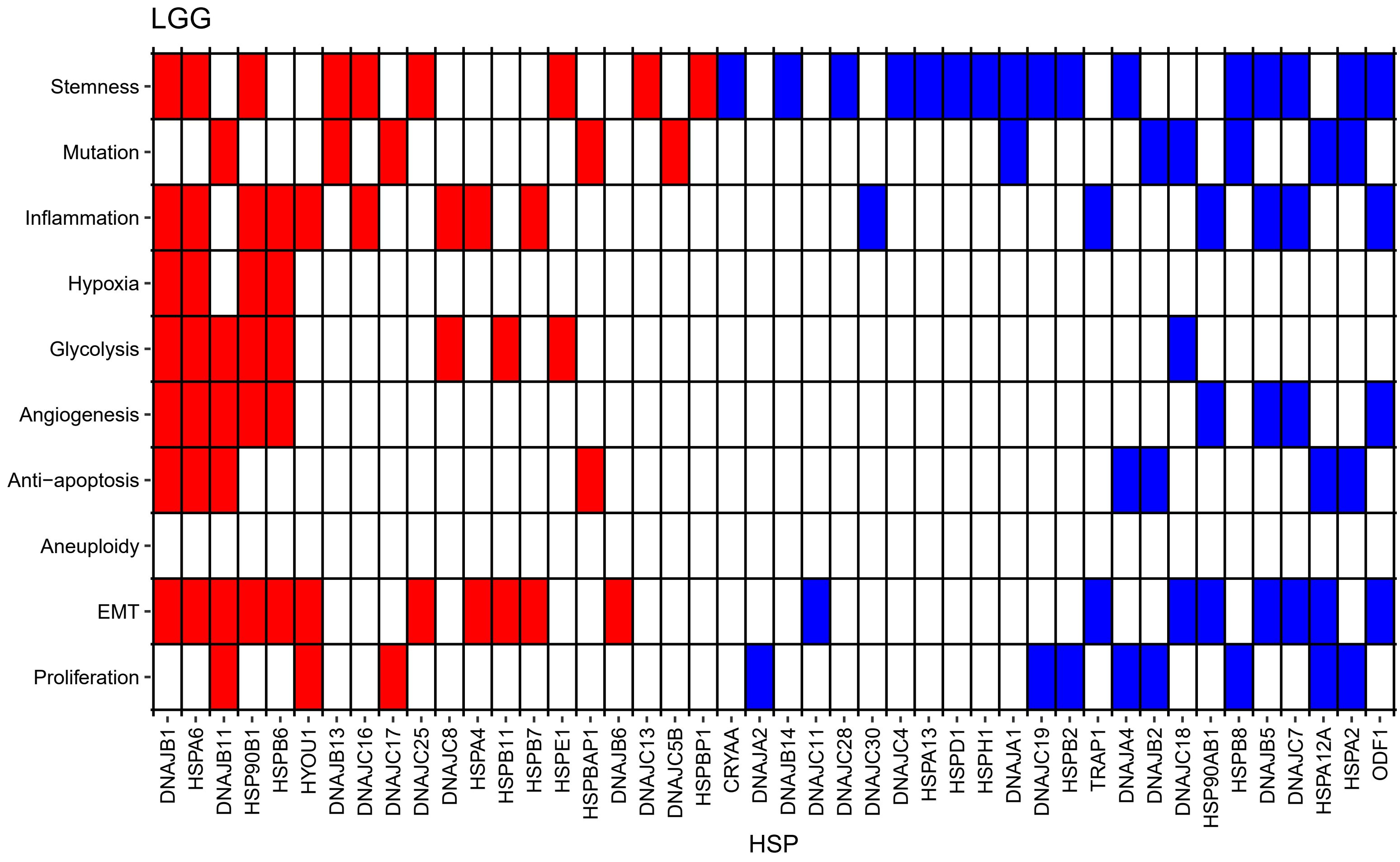
LGG
Stemness
Mutation
Inflammation
Hypoxia
Glycolysis
Angiogenesis
Anti−apoptosis
Aneuploidy
EMT
Proliferation
DNAJB1
HSPA6
DNAJB11
HSP90B1
HSPB6
HYOU1
DNAJB13
DNAJC16
DNAJC17
DNAJC25
DNAJC8
HSPA4
HSPB11
HSPB7
HSPE1
HSPBAP1
DNAJB6
DNAJC13
DNAJC5B
HSPBP1
CRYAA
DNAJA2
DNAJB14
DNAJC11
DNAJC28
DNAJC30
DNAJC4
HSPA13
HSPD1
HSPH1
DNAJA1
DNAJC19
HSPB2
TRAP1
DNAJA4
DNAJB2
DNAJC18
HSP90AB1
HSPB8
DNAJB5
DNAJC7
HSPA12A
HSPA2
ODF1
HSP

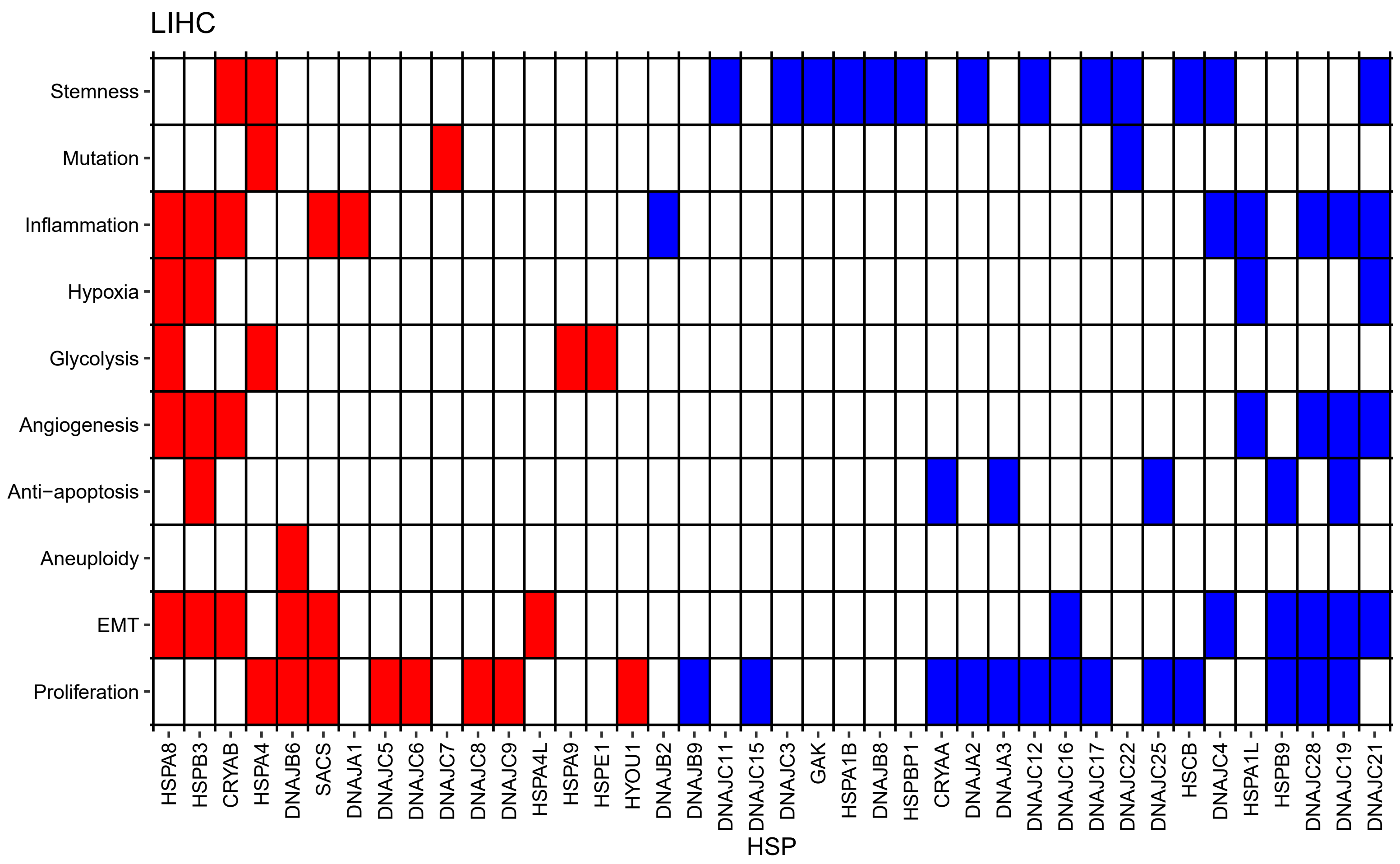

LIHC
Stemness
Mutation
Inflammation
Hypoxia
Glycolysis
Angiogenesis
Anti−apoptosis
Aneuploidy
EMT
Proliferation
HSPA8
HSPB3
CRYAB
HSPA4
DNAJB6
SACS
DNAJA1
DNAJC5
DNAJC6
DNAJC7
DNAJC8
DNAJC9
HSPA4L
HSPA9
HSPE1
HYOU1
DNAJB2
DNAJB9
DNAJC11
DNAJC15
DNAJC3
GAK
HSPA1B
DNAJB8
HSPBP1
CRYAA
DNAJA2
DNAJA3
DNAJC12
DNAJC16
DNAJC17
DNAJC22
DNAJC25
HSCB
DNAJC4
HSPA1L
HSPB9
DNAJC28
DNAJC19
DNAJC21
HSP

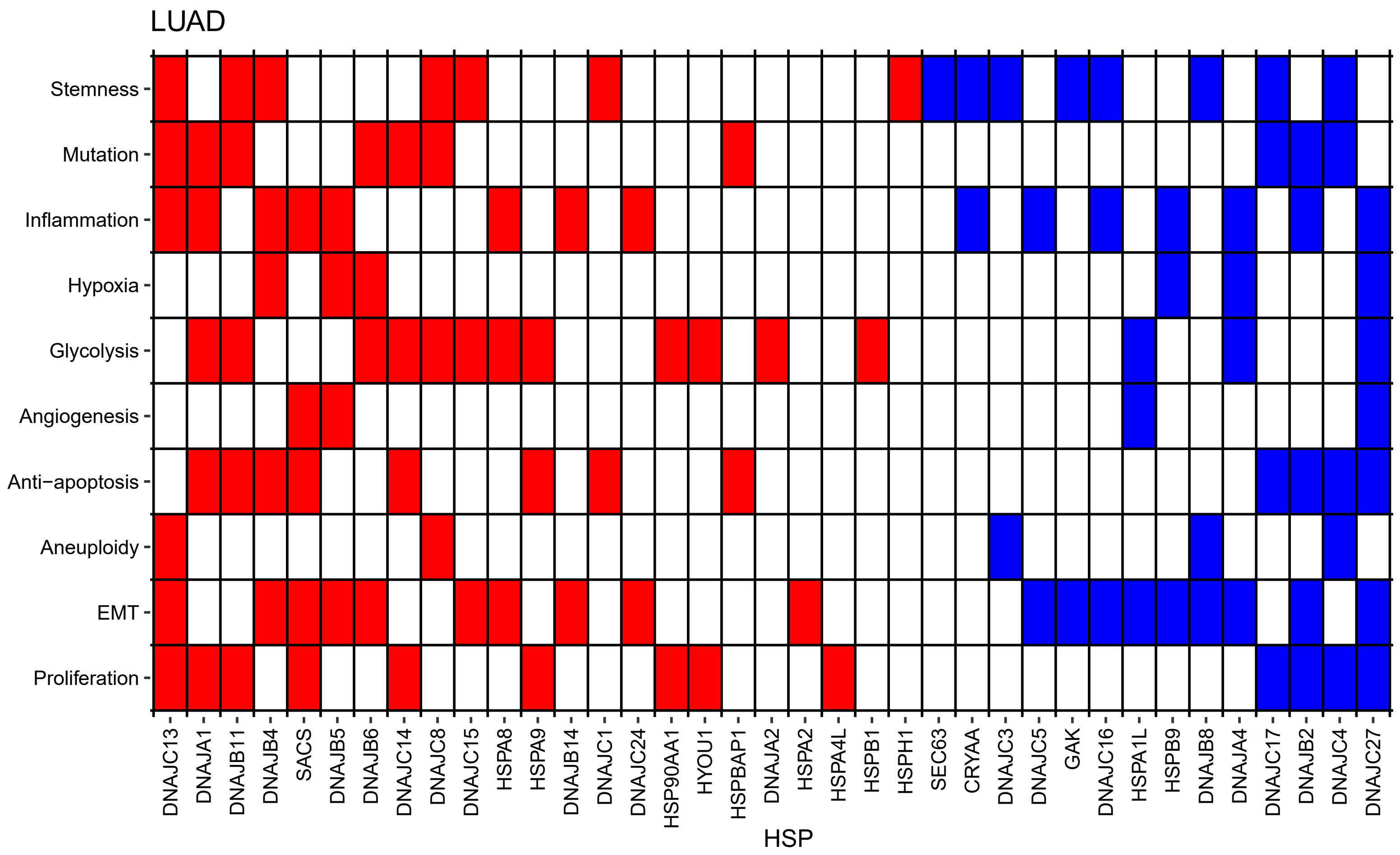

LUAD
Stemness
Mutation
Inflammation
Hypoxia
Glycolysis
Angiogenesis
Anti−apoptosis
Aneuploidy
EMT
Proliferation
DNAJC13
DNAJA1
DNAJB11
DNAJB4
SACS
DNAJB5
DNAJB6
DNAJC14
DNAJC8
DNAJC15
HSPA8
HSPA9
DNAJB14
DNAJC1
DNAJC24
HSP90AA1
HYOU1
HSPBAP1
DNAJA2
HSPA2
HSPA4L
HSPB1
HSPH1
SEC63
CRYAA
DNAJC3
DNAJC5
GAK
DNAJC16
HSPA1L
HSPB9
DNAJB8
DNAJA4
DNAJC17
DNAJB2
DNAJC4
DNAJC27
HSP

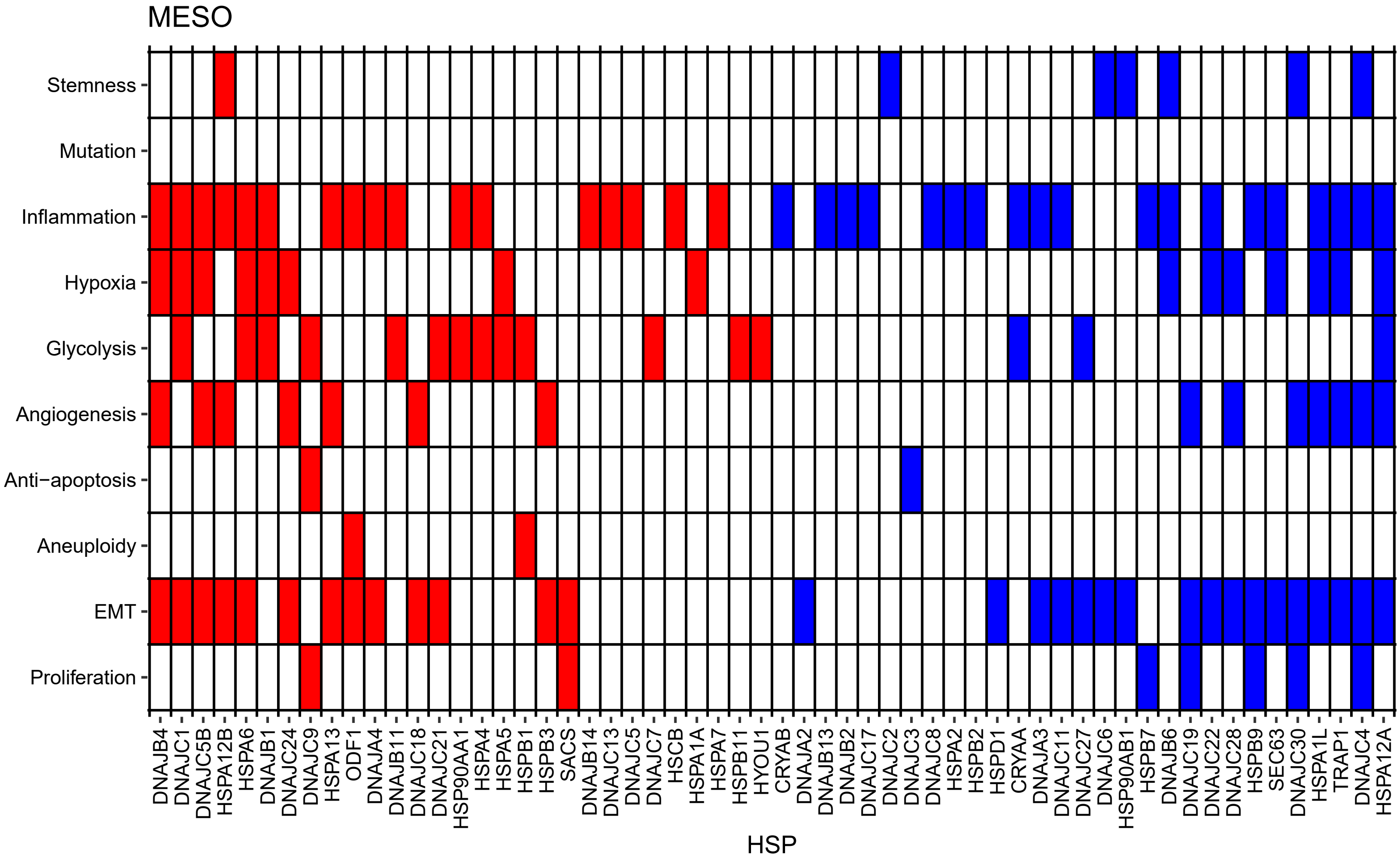
MESO
Stemness
Mutation
Inflammation
Hypoxia
Glycolysis
Angiogenesis
Anti−apoptosis
Aneuploidy
EMT
Proliferation
DNAJB4 DNAJC1 DNAJC5B HSPA12B HSPA6 DNAJB1 DNAJC24 DNAJC9 HSPA13 ODF1 DNAJA4 DNAJB11 DNAJC18 DNAJC21 HSP90AA1 HSPA4 HSPA5 HSPB1 HSPB3 SACS DNAJB14 DNAJC13 DNAJC5 DNAJC7 HSCB HSPA1A HSPA7 HSPB11 HYOU1 CRYAB DNAJA2 DNAJB13 DNAJB2 DNAJC17 DNAJC2 DNAJC3 DNAJC8 HSPA2 HSPB2 HSPD1 CRYAA DNAJA3 DNAJC11 DNAJC27 DNAJC6 HSP90AB1 HSPB7 DNAJB6 DNAJC19 DNAJC22 DNAJC28 HSPB9 SEC63 DNAJC30 HSPA1L TRAP1 DNAJC4 HSPA12A
HSP

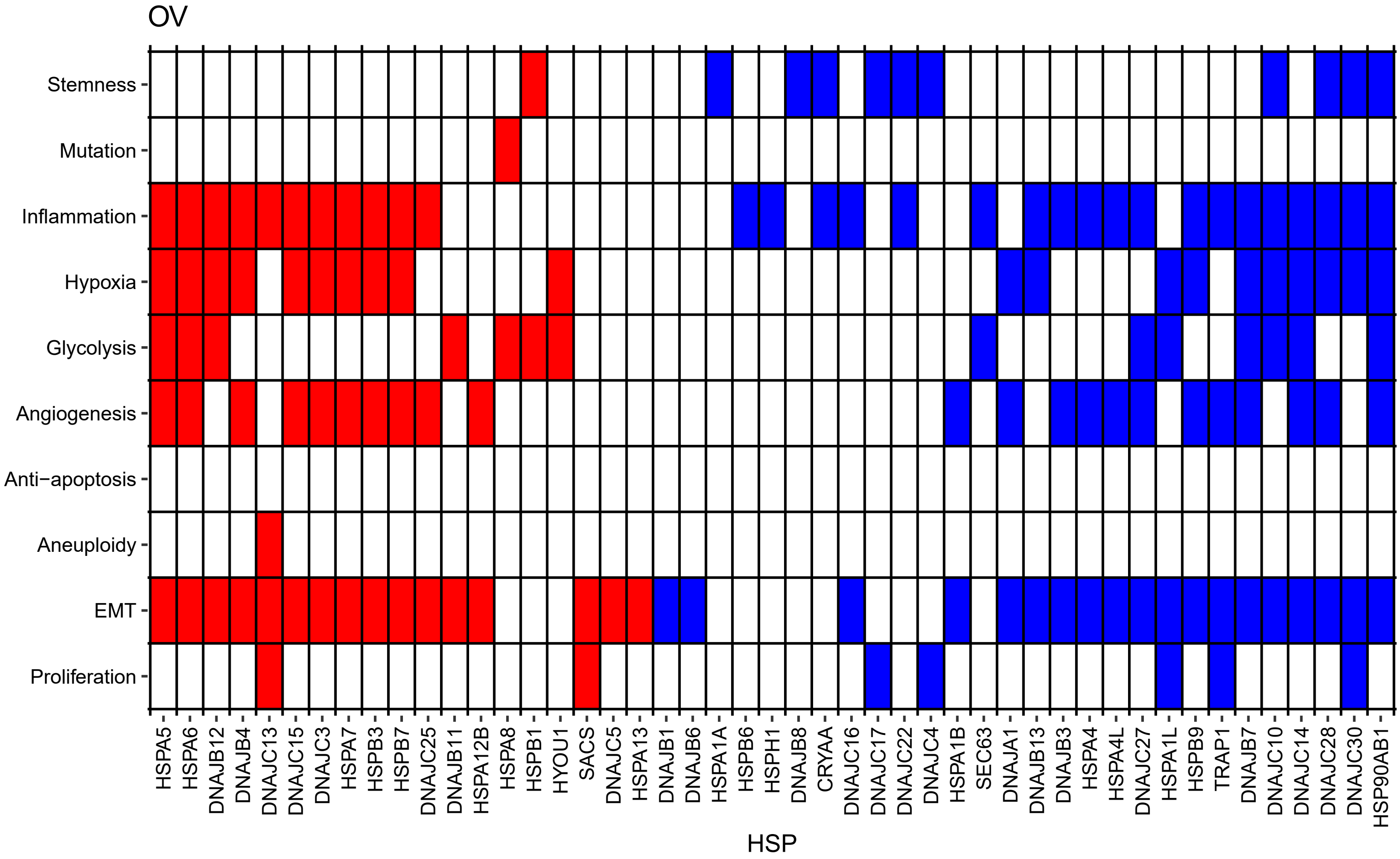
OV
Stemness
Mutation
Inflammation
Hypoxia
Glycolysis
Angiogenesis
Anti−apoptosis
Aneuploidy
EMT
Proliferation
HSPA5 HSPA6 DNAJB12 DNAJB4 DNAJC13 DNAJC15 DNAJC3 HSPA7 HSPB3 HSPB7 DNAJC25 DNAJB11 HSPA12B HSPA8 HSPB1 HYOU1 SACS DNAJC5 HSPA13 DNAJB1 DNAJB6 HSPA1A HSPB6 HSPH1 DNAJB8 CRYAA DNAJC16 DNAJC17 DNAJC22 DNAJC4 HSPA1B SEC63 DNAJA1 DNAJB13 DNAJB3 HSPA4 HSPA4L DNAJC27 HSPA1L HSPB9 TRAP1 DNAJB7 DNAJC10 DNAJC14 DNAJC28 DNAJC30 HSP90AB1
HSP

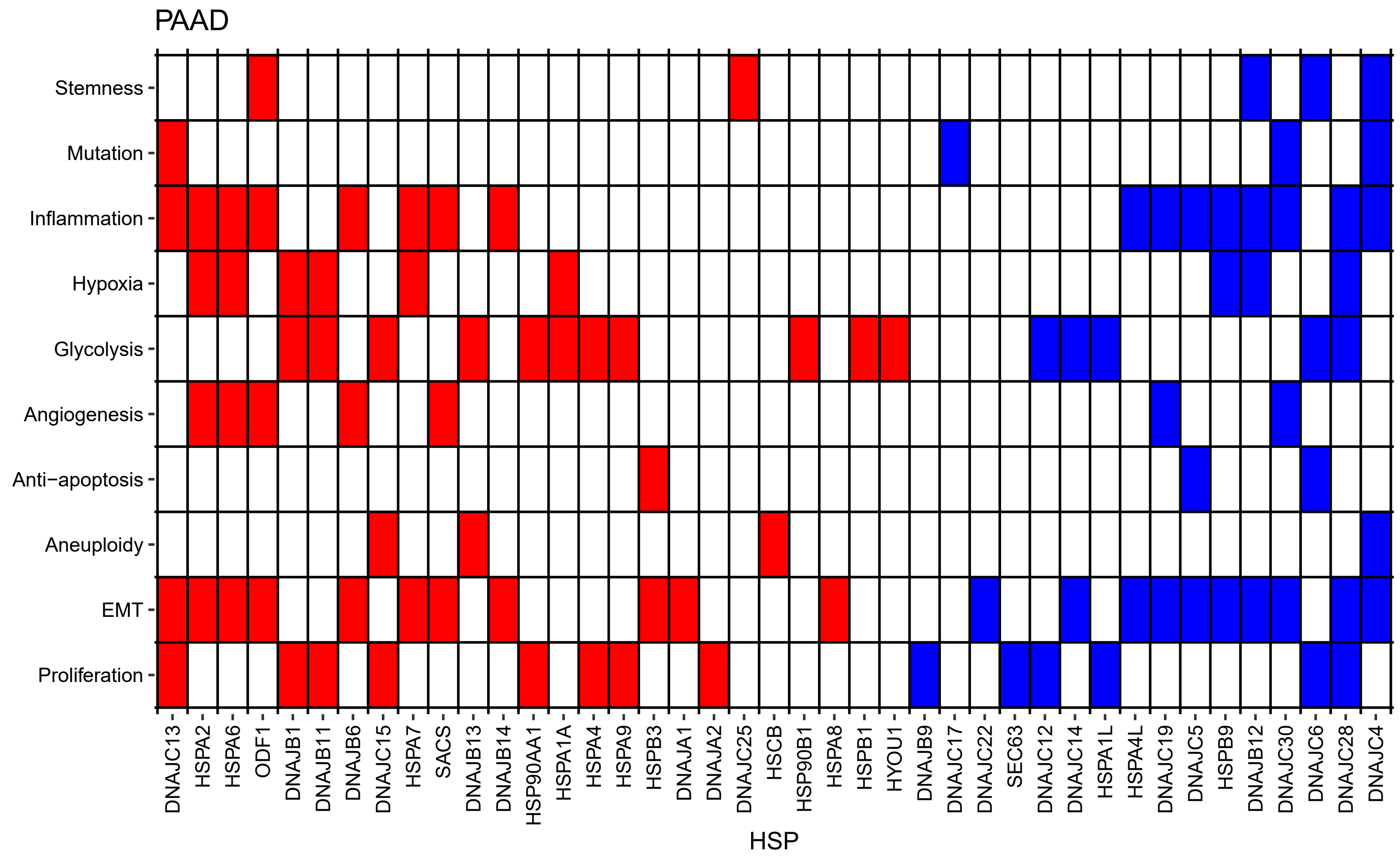
PAAD
Stemness
Mutation
Inflammation
Hypoxia
Glycolysis
Angiogenesis
Anti−apoptosis
Aneuploidy
EMT
Proliferation
DNAJC13
HSPA2
HSPA6
ODF1
DNAJB1
DNAJB11
DNAJB6
DNAJC15
HSPA7
SACS
DNAJB13
DNAJB14
HSP90AA1
HSPA1A
HSPA4
HSPA9
HSPB3
DNAJA1
DNAJA2
DNAJC25
HSCB
HSP90B1
HSPA8
HSPB1
HYOU1
DNAJB9
DNAJC17
DNAJC22
SEC63
DNAJC12
DNAJC14
HSPA1L
HSPA4L
DNAJC19
DNAJC5
HSPB9
DNAJB12
DNAJC30
DNAJC6
DNAJC28
DNAJC4
HSP

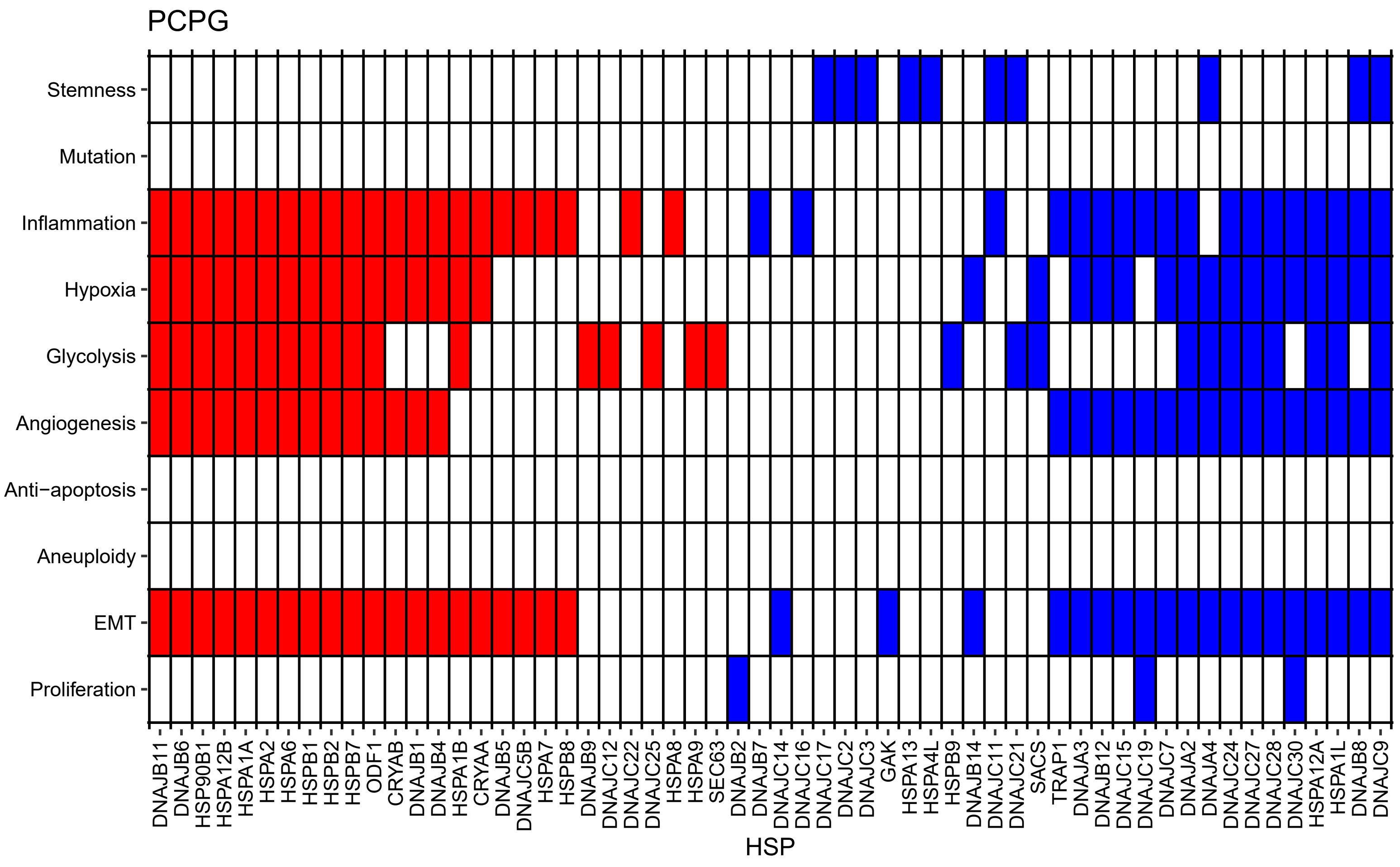
PCPG
Stemness
Mutation
Inflammation
Hypoxia
Glycolysis
Angiogenesis
Anti−apoptosis
Aneuploidy
EMT
Proliferation
DNAJB11
DNAJB6
HSP90B1
HSPA12B
HSPA1A
HSPA2
HSPA6
HSPB1
HSPB2
HSPB7
ODF1
CRYAB
DNAJB1
DNAJB4
HSPA1B
CRYAA
DNAJB5
DNAJC5B
HSPA7
HSPB8
DNAJB9
DNAJC12
DNAJC22
DNAJC25
HSPA8
HSPA9
SEC63
DNAJB2
DNAJB7
DNAJC14
DNAJC16
DNAJC17
DNAJC2
DNAJC3
GAK
HSPA13
HSPA4L
HSPB9
DNAJB14
DNAJC11
DNAJC21
SACS
TRAP1
DNAJA3
DNAJB12
DNAJC15
DNAJC19
DNAJC7
DNAJA2
DNAJA4
DNAJC24
DNAJC27
DNAJC28
DNAJC30
HSPA12A
HSPA1L
DNAJB8
DNAJC9
HSP

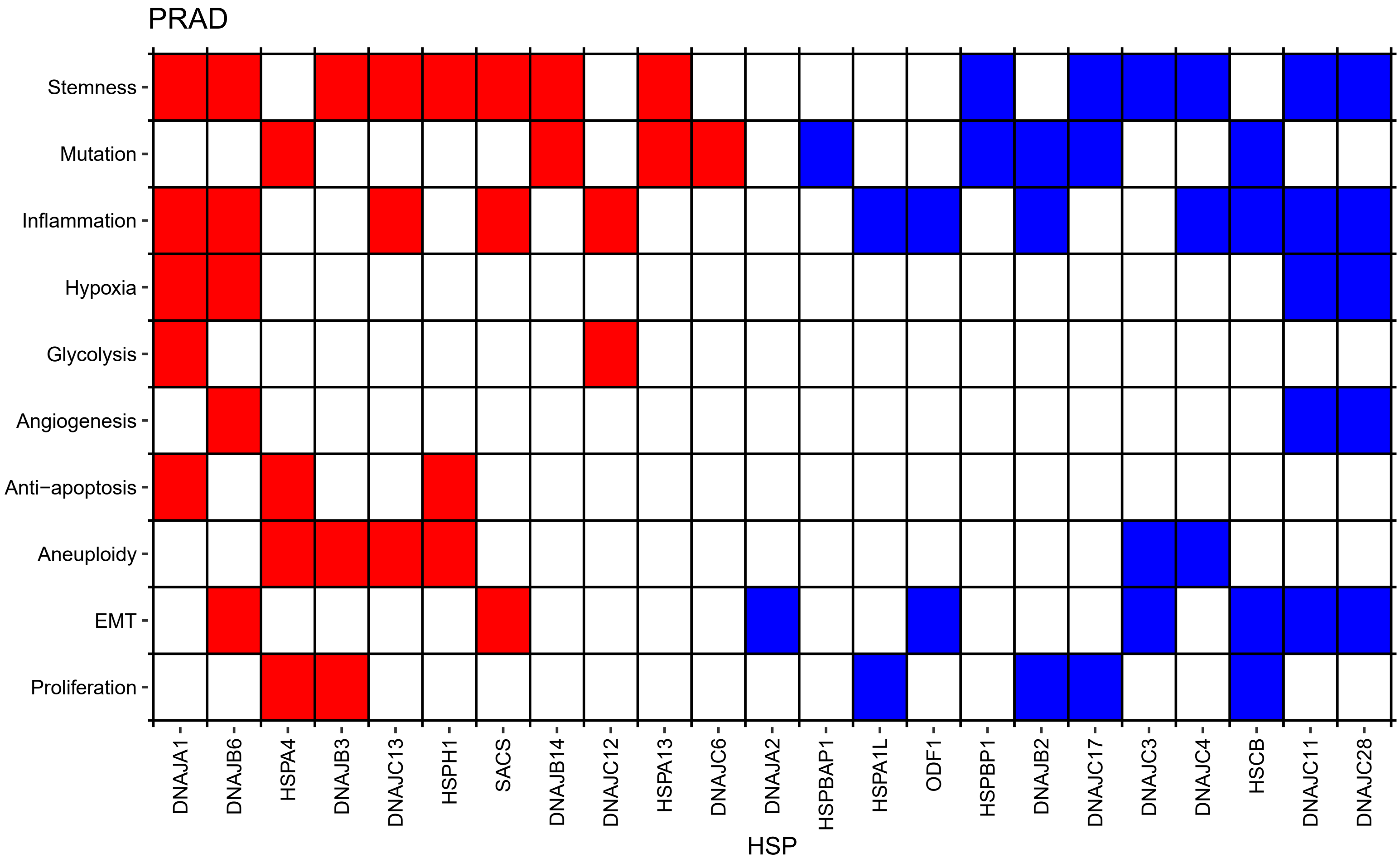
PRAD
Stemness
Mutation
Inflammation
Hypoxia
Glycolysis
Angiogenesis
Anti−apoptosis
Aneuploidy
EMT
Proliferation
DNAJA1
DNAJB6
HSPA4
DNAJB3
DNAJC13
HSPH1
SACS
DNAJB14
DNAJC12
HSPA13
DNAJC6
DNAJA2
HSPBAP1
HSPA1L
ODF1
HSPBP1
DNAJB2
DNAJC17
DNAJC3
DNAJC4
HSCB
DNAJC11
DNAJC28
HSP

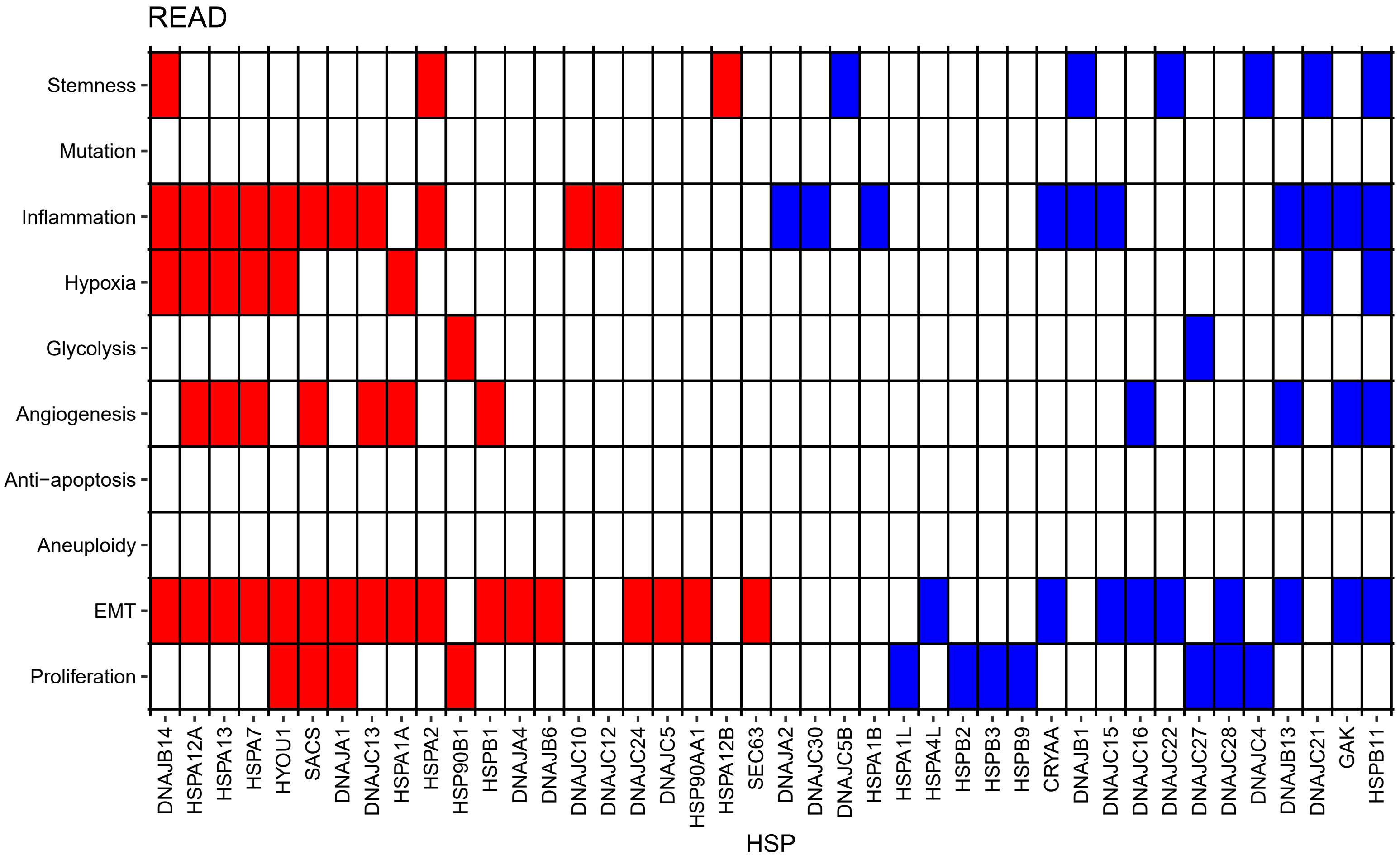
READ
Stemness
Mutation
Inflammation
Hypoxia
Glycolysis
Angiogenesis
Anti−apoptosis
Aneuploidy
EMT
Proliferation
DNAJB14
HSPA12A
HSPA13
HSPA7
HYOU1
SACS
DNAJA1
DNAJC13
HSPA1A
HSPA2
HSP90B1
HSPB1
DNAJA4
DNAJB6
DNAJC10
DNAJC12
DNAJC24
DNAJC5
HSP90AA1
HSPA12B
SEC63
DNAJA2
DNAJC30
DNAJC5B
HSPA1B
HSPA1L
HSPA4L
HSPB2
HSPB3
HSPB9
CRYAA
DNAJB1
DNAJC15
DNAJC16
DNAJC22
DNAJC27
DNAJC28
DNAJC4
DNAJB13
DNAJC21
GAK
HSPB11
HSP

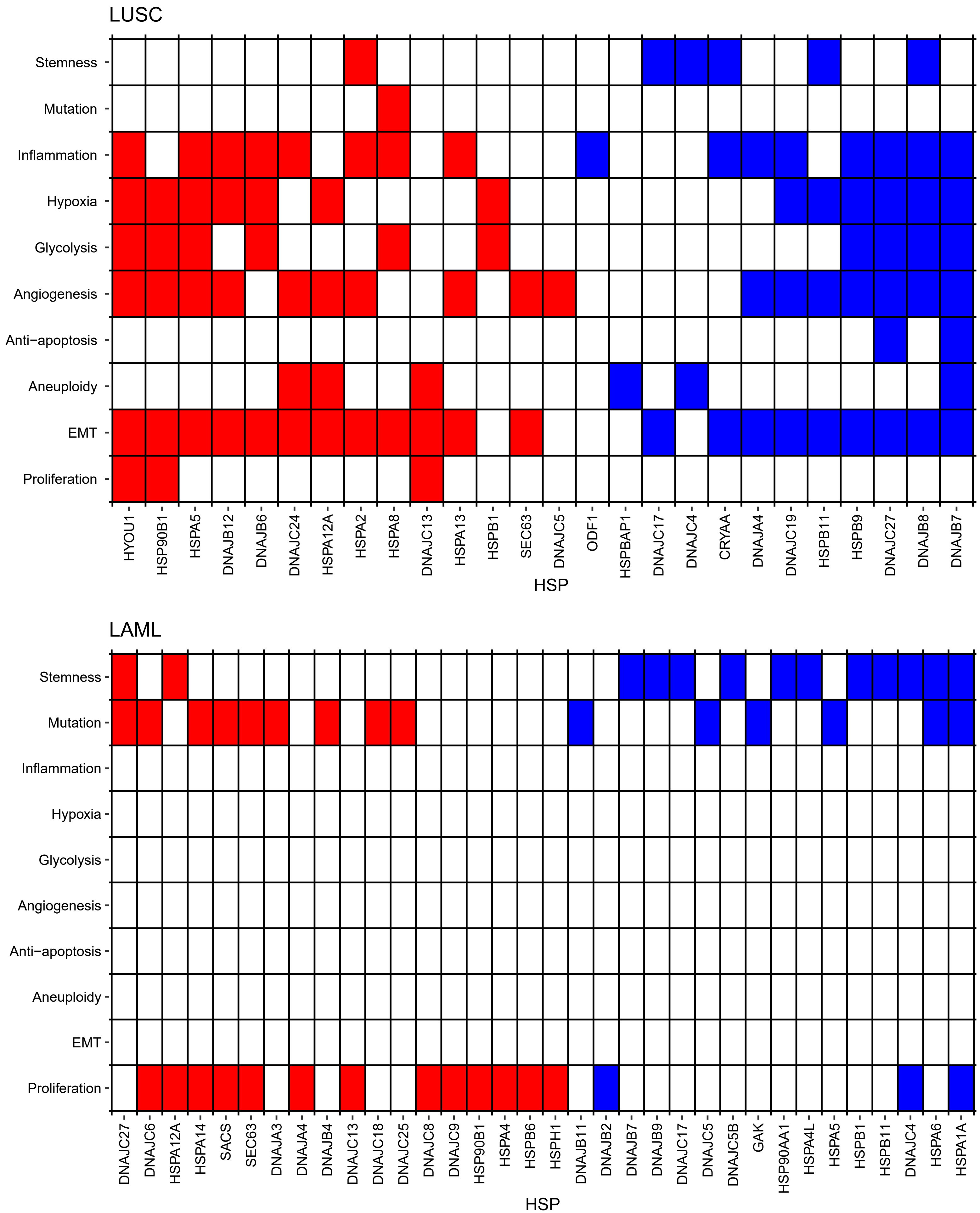
LUSC
Stemness
Mutation
Inflammation
Hypoxia
Glycolysis
Angiogenesis
Anti−apoptosis
Aneuploidy
EMT
Proliferation
HYOU1
HSP90B1
HSPA5
DNAJB12
DNAJB6
DNAJC24
HSPA12A
HSPA2
HSPA8
DNAJC13
HSPA13
HSPB1
SEC63
DNAJC5
ODF1
HSPBAP1
DNAJC17
DNAJC4
CRYAA
DNAJA4
DNAJC19
HSPB11
HSPB9
DNAJC27
DNAJB8
DNAJB7
HSP
LAML
Stemness
Mutation
Inflammation
Hypoxia
Glycolysis
Angiogenesis
Anti−apoptosis
Aneuploidy
EMT
Proliferation
DNAJC27
DNAJC6
HSPA12A
HSPA14
SACS
SEC63
DNAJA3
DNAJA4
DNAJB4
DNAJC13
DNAJC18
DNAJC25
DNAJC8
DNAJC9
HSP90B1
HSPA4
HSPB6
HSPH1
DNAJB11
DNAJB2
DNAJB7
DNAJB9
DNAJC17
DNAJC5
DNAJC5B
GAK
HSP90AA1
HSPA4L
HSPA5
HSPB1
HSPB11
DNAJC4
HSPA6
HSPA1A
HSP

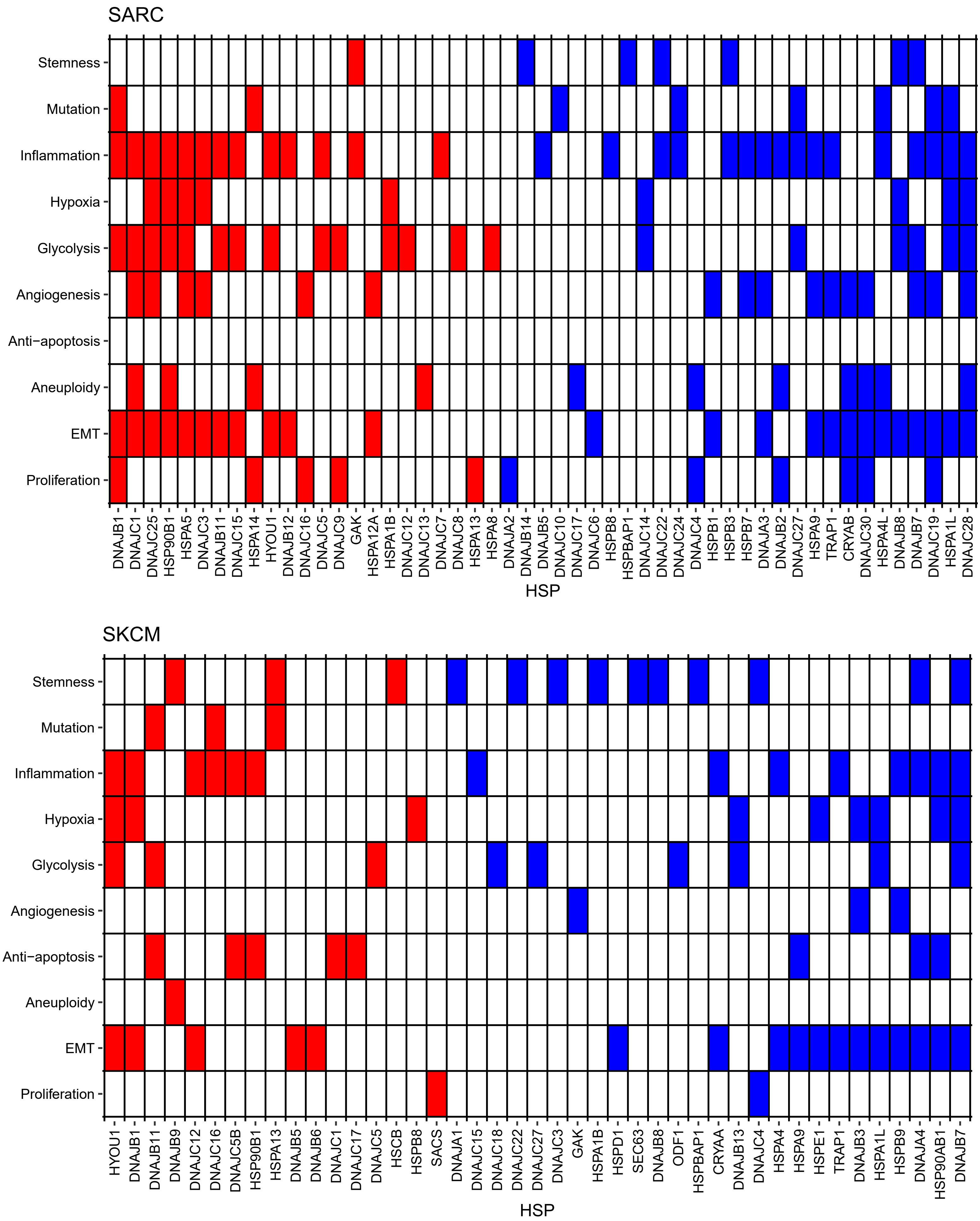
SARC
Stemness
Mutation
Inflammation
Hypoxia
Glycolysis
Angiogenesis
Anti−apoptosis
Aneuploidy
EMT
Proliferation
DNAJB1 DNAJC1 DNAJC25 HSP90B1 HSPA5 DNAJC3 DNAJB11 DNAJC15 HSPA14 HYOU1 DNAJB12 DNAJC16 DNAJC5 DNAJC9 GAK HSPA12A HSPA1B DNAJC12 DNAJC13 DNAJC7 DNAJC8 HSPA13 HSPA8 DNAJA2 DNAJB14 DNAJB5 DNAJC10 DNAJC17 DNAJC6 HSPB8 HSPBAP1 DNAJC14 DNAJC22 DNAJC24 DNAJC4 HSPB1 HSPB3 HSPB7 DNAJA3 DNAJB2 DNAJC27 HSPA9 TRAP1 CRYAB DNAJC30 HSPA4L DNAJB8 DNAJB7 DNAJC19 HSPA1L DNAJC28
HSP
SKCM
Stemness
Mutation
Inflammation
Hypoxia
Glycolysis
Angiogenesis
Anti−apoptosis
Aneuploidy
EMT
Proliferation
HYOU1 DNAJB1 DNAJB11 DNAJB9 DNAJC12 DNAJC16 DNAJC5B HSP90B1 HSPA13 DNAJB5 DNAJB6 DNAJC1 DNAJC17 DNAJC5 HSCB HSPB8 SACS DNAJA1 DNAJC15 DNAJC18 DNAJC22 DNAJC27 DNAJC3 GAK HSPA1B HSPD1 SEC63 DNAJB8 ODF1 HSPBAP1 CRYAA DNAJB13 DNAJC4 HSPA4 HSPA9 HSPE1 TRAP1 DNAJB3 HSPA1L HSPB9 DNAJA4 HSP90AB1 DNAJB7
HSP

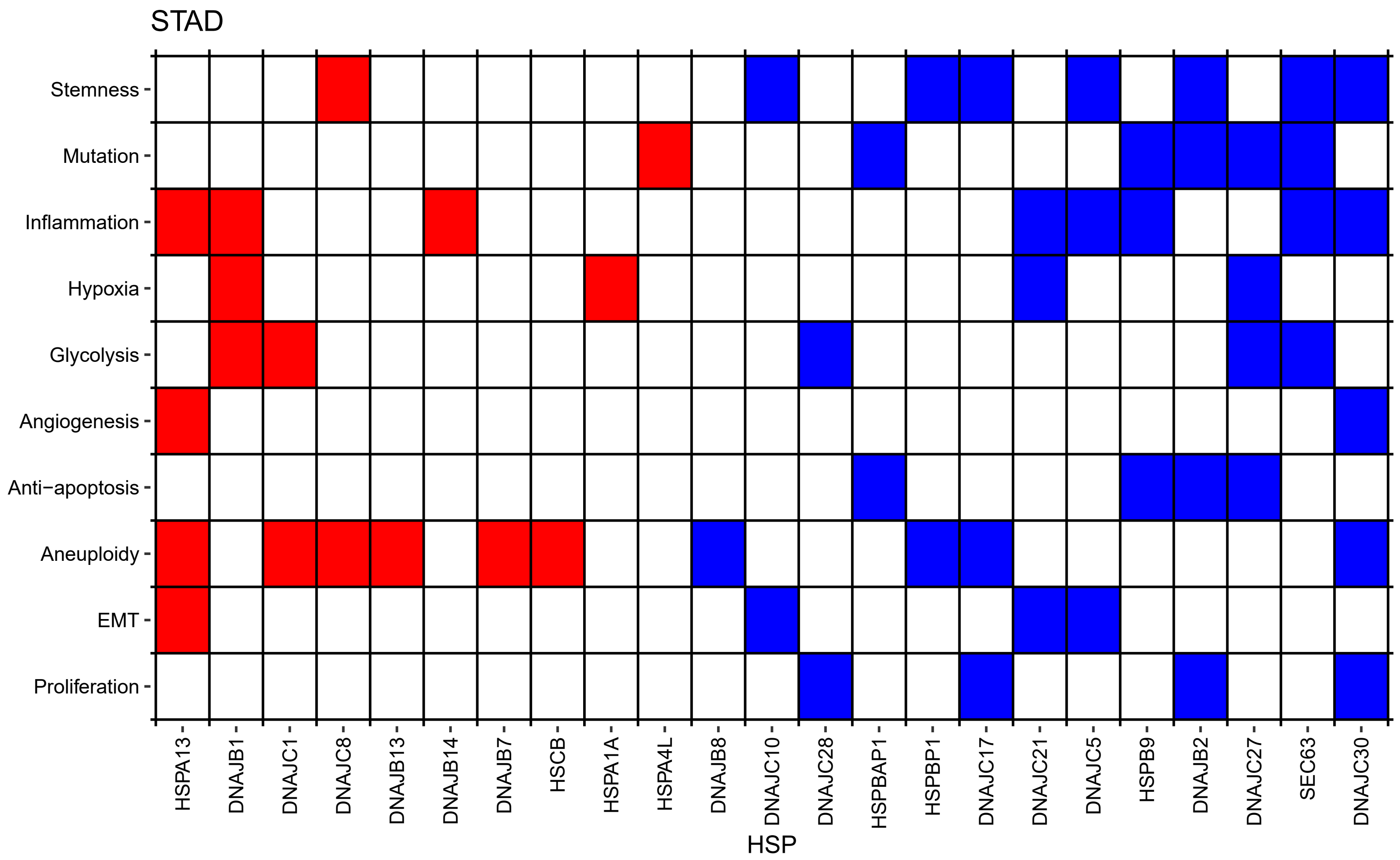
STAD
Stemness
Mutation
Inflammation
Hypoxia
Glycolysis
Angiogenesis
Anti−apoptosis
Aneuploidy
EMT
Proliferation
HSPA13
DNAJB1
DNAJC1
DNAJC8
DNAJB13
DNAJB14
DNAJB7
HSCB
HSPA1A
HSPA4L
DNAJB8
DNAJC10
DNAJC28
HSPBAP1
HSPBP1
DNAJC17
DNAJC21
DNAJC5
HSPB9
DNAJB2
DNAJC27
SEC63
DNAJC30
HSP

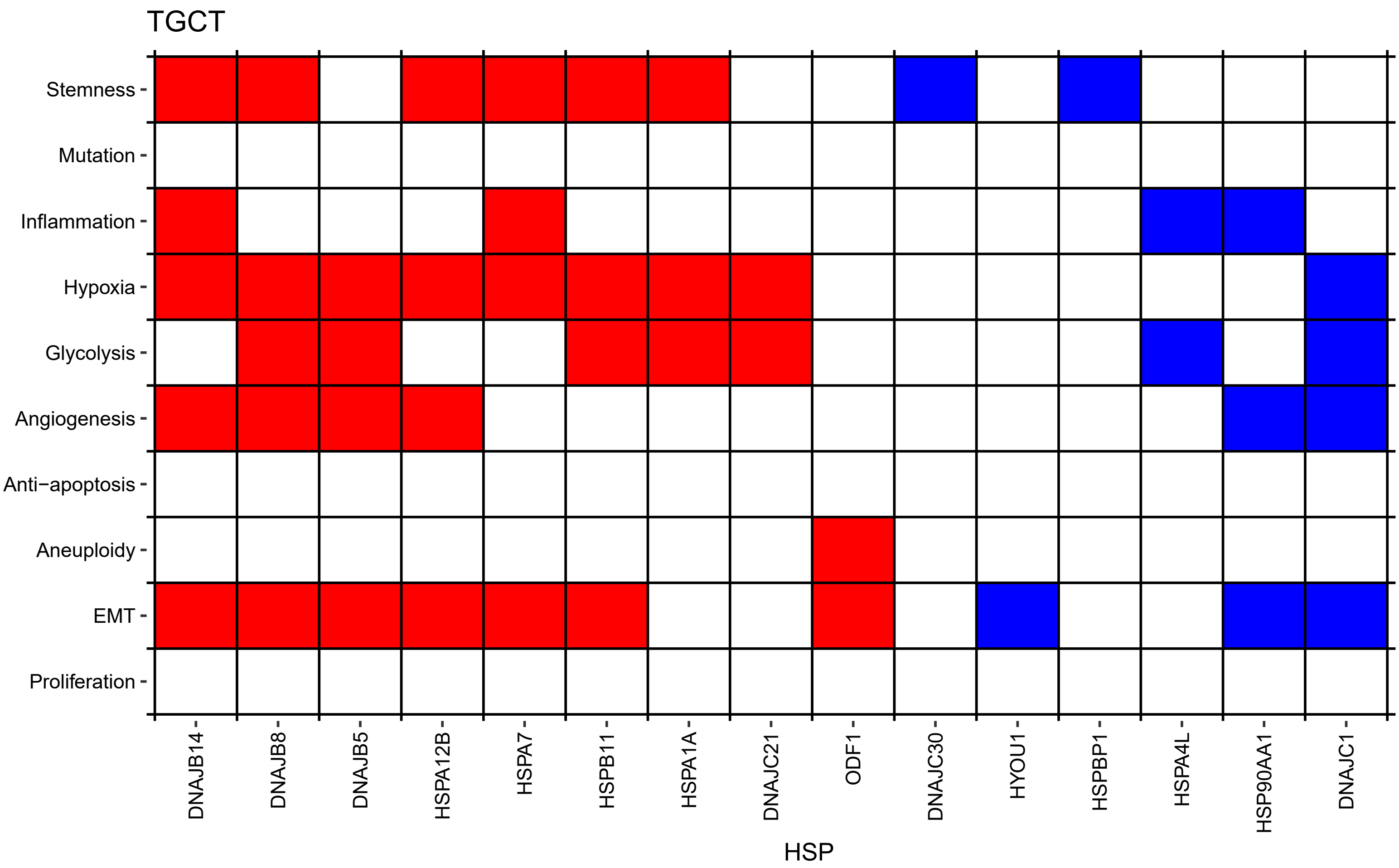
TGCT
Stemness
Mutation
Inflammation
Hypoxia
Glycolysis
Angiogenesis
Anti−apoptosis
Aneuploidy
EMT
Proliferation
DNAJB14
DNAJB8
DNAJB5
HSPA12B
HSPA7
HSPB11
HSPA1A
DNAJC21
ODF1
DNAJC30
HYOU1
HSPBP1
HSPA4L
HSP90AA1
DNAJC1
HSP

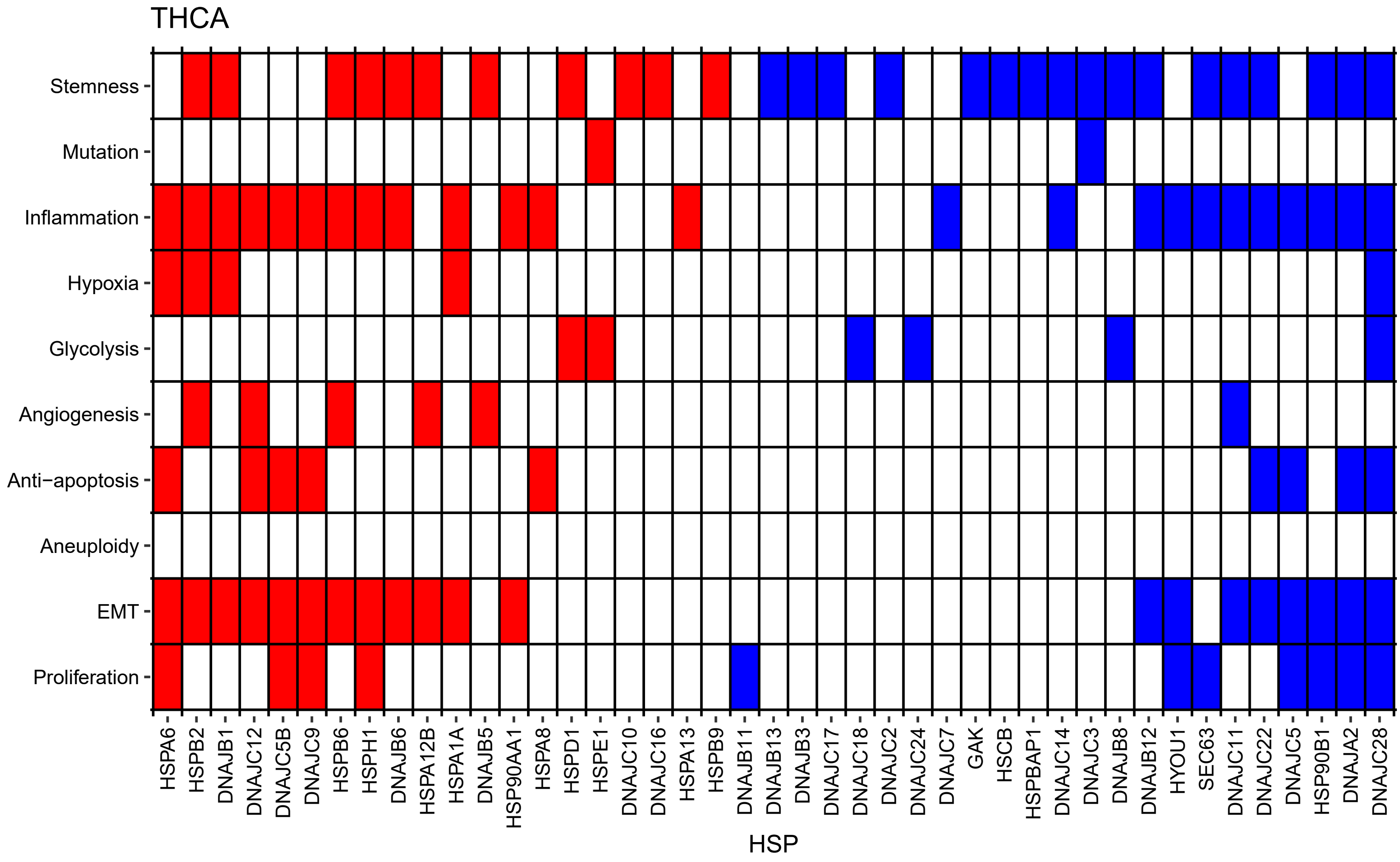
THCA
Stemness
Mutation
Inflammation
Hypoxia
Glycolysis
Angiogenesis
Anti−apoptosis
Aneuploidy
EMT
Proliferation
HSPA6
HSPB2
DNAJB1
DNAJC12
DNAJC5B
DNAJC9
HSPB6
HSPH1
DNAJB6
HSPA12B
HSPA1A
DNAJB5
HSP90AA1
HSPA8
HSPD1
HSPE1
DNAJC10
DNAJC16
HSPA13
HSPB9
DNAJB11
DNAJB13
DNAJB3
DNAJC17
DNAJC18
DNAJC2
DNAJC24
DNAJC7
GAK
HSCB
HSPBAP1
DNAJC14
DNAJC3
DNAJB8
DNAJB12
HYOU1
SEC63
DNAJC11
DNAJC22
DNAJC5
HSP90B1
DNAJA2
DNAJC28
HSP

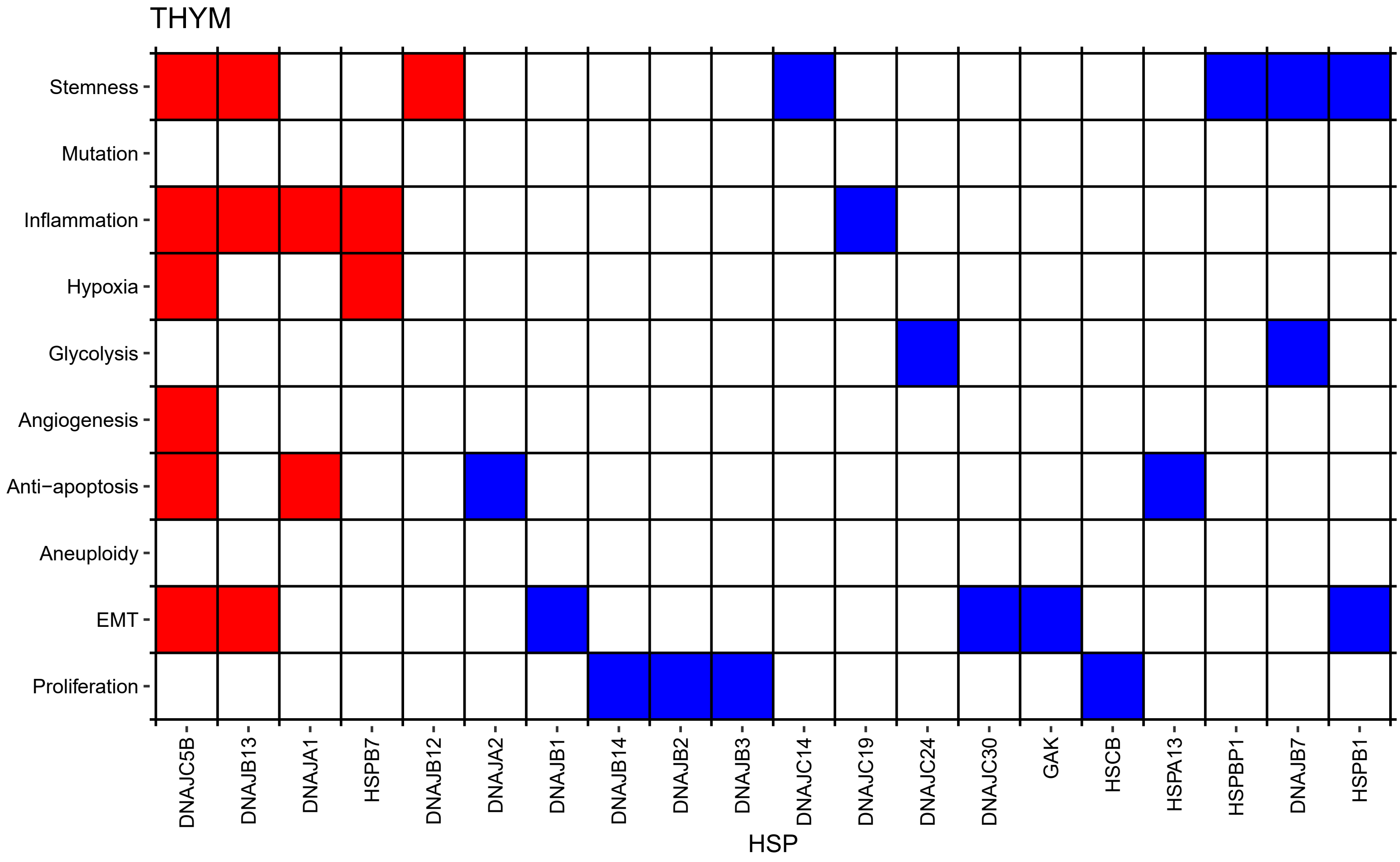
THYM
Stemness
Mutation
Inflammation
Hypoxia
Glycolysis
Angiogenesis
Anti−apoptosis
Aneuploidy
EMT
Proliferation
DNAJC5B
DNAJB13
DNAJA1
HSPB7
DNAJB12
DNAJA2
DNAJB1
DNAJB14
DNAJB2
DNAJB3
DNAJC14
DNAJC19
DNAJC24
DNAJC30
GAK
HSCB
HSPA13
HSPBP1
DNAJB7
HSPB1
HSP

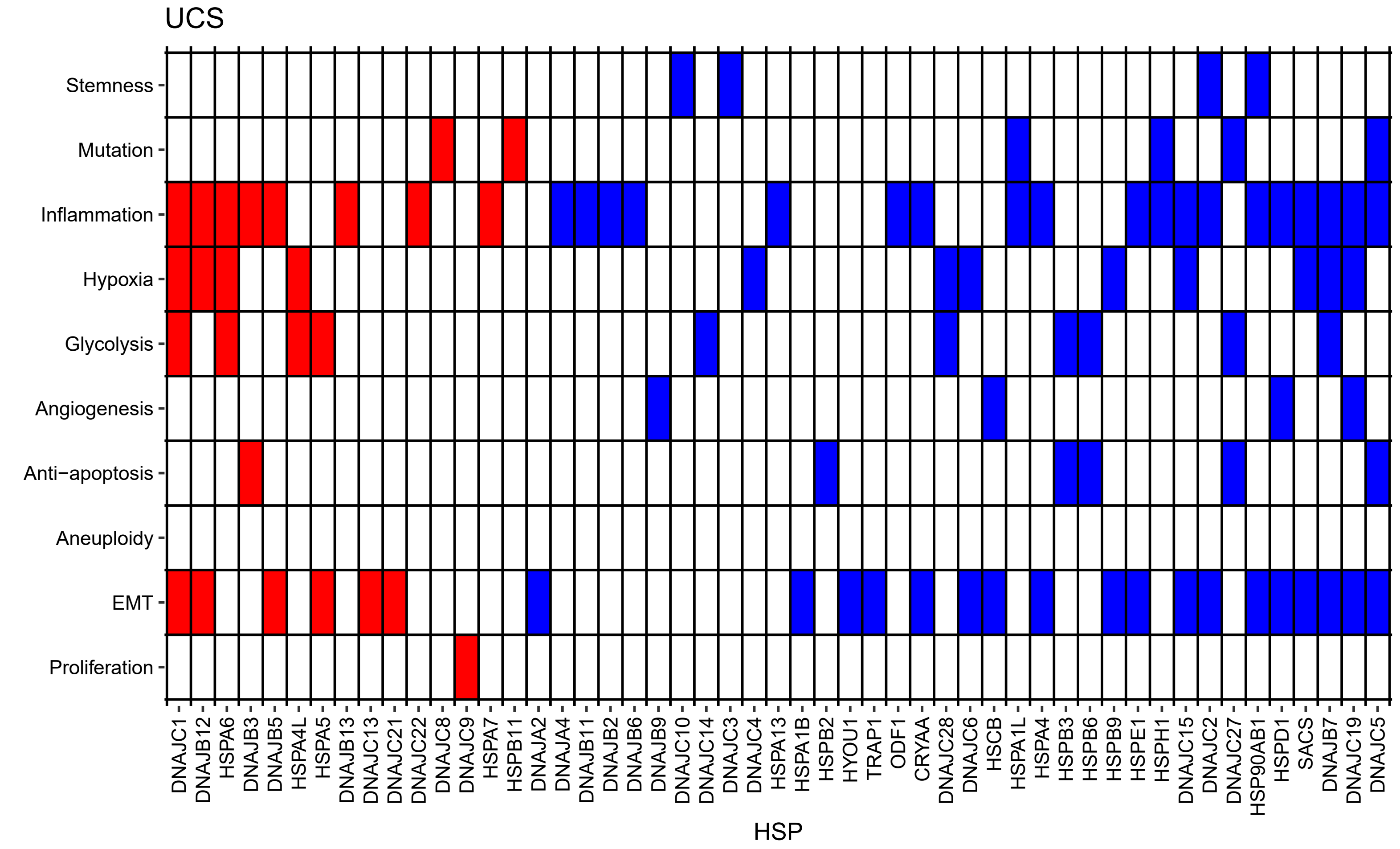
UCS
Stemness
Mutation
Inflammation
Hypoxia
Glycolysis
Angiogenesis
Anti−apoptosis
Aneuploidy
EMT
Proliferation
DNAJC1
DNAJB12
HSPA6
DNAJB3
DNAJB5
HSPA4L
HSPA5
DNAJB13
DNAJC13
DNAJC21
DNAJC22
DNAJC8
DNAJC9
HSPA7
HSPB11
DNAJA2
DNAJA4
DNAJB11
DNAJB2
DNAJB6
DNAJB9
DNAJC10
DNAJC14
DNAJC3
DNAJC4
HSPA13
HSPA1B
HSPB2
HYOU1
TRAP1
ODF1
CRYAA
DNAJC28
DNAJC6
HSCB
HSPA1L
HSPA4
HSPB3
HSPB6
HSPB9
HSPE1
HSPH1
DNAJC15
DNAJC2
DNAJC27
HSP90AB1
HSPD1
SACS
DNAJB7
DNAJC19
DNAJC5
HSP

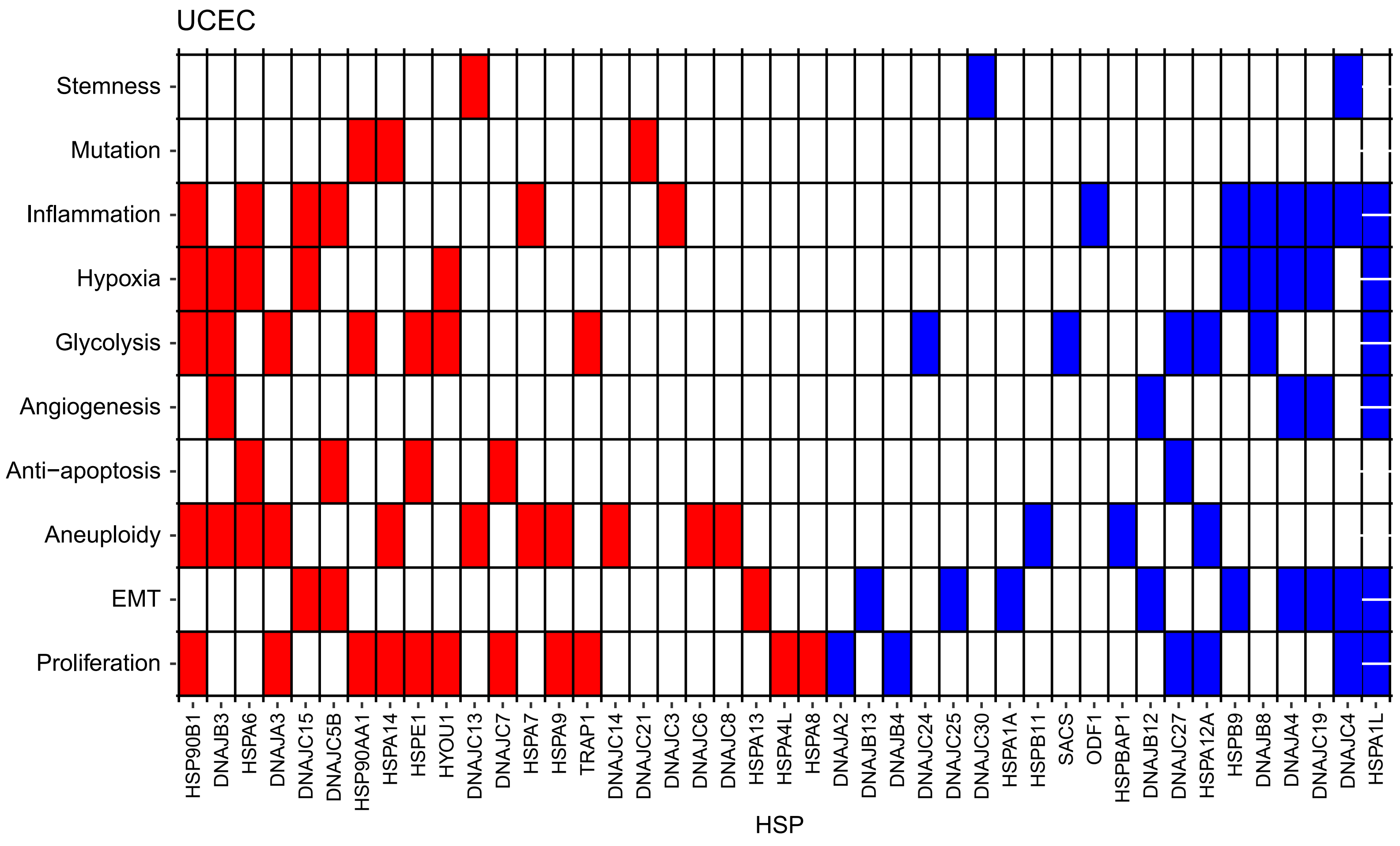
UCEC
Stemness
Mutation
Inflammation
Hypoxia
Glycolysis
Angiogenesis
Anti−apoptosis
Aneuploidy
EMT
Proliferation
HSP90B1
DNAJB3
HSPA6
DNAJA3
DNAJC15
DNAJC5B
HSP90AA1
HSPA14
HSPE1
HYOU1
DNAJC13
DNAJC7
HSPA7
HSPA9
TRAP1
DNAJC14
DNAJC21
DNAJC3
DNAJC6
DNAJC8
HSPA13
HSPA4L
HSPA8
DNAJA2
DNAJB13
DNAJB4
DNAJC24
DNAJC25
DNAJC30
HSPA1A
HSPB11
SACS
ODF1
HSPBAP1
DNAJB12
DNAJC27
HSPA12A
HSPB9
DNAJB8
DNAJA4
DNAJC19
DNAJC4
HSPA1L
HSP

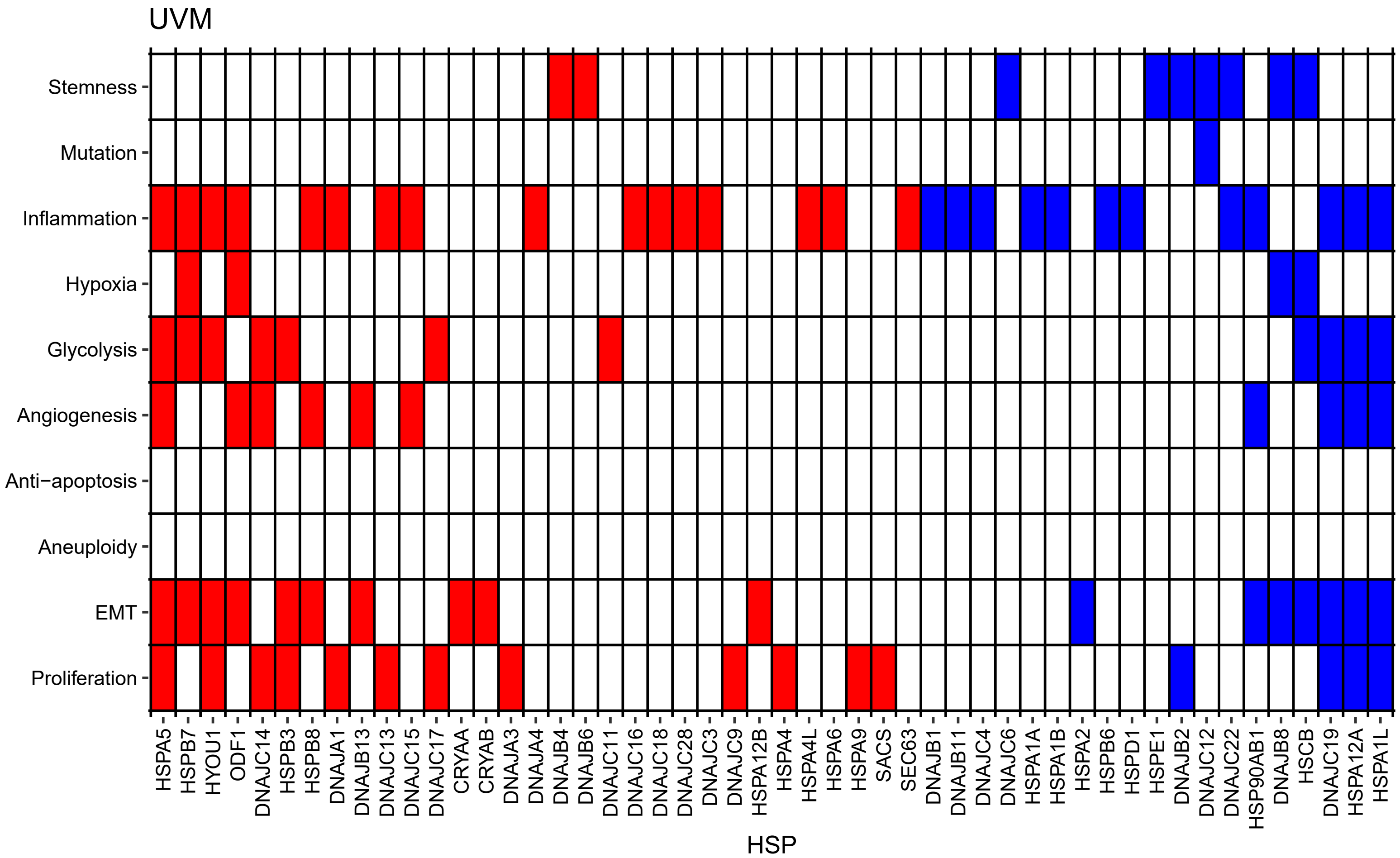

Figure S6. HSPs associated with cancer hallmark across cancer types. A) Number of HSPs associated with each cancer hallmark in at least one cancer type. **B)** Associations between HSPs and cancer hallmarks across cancer types. **C)** Prioritized HSPs which have consistent direction with multiple cancer hallmarks in each cancer types.

Figure S7

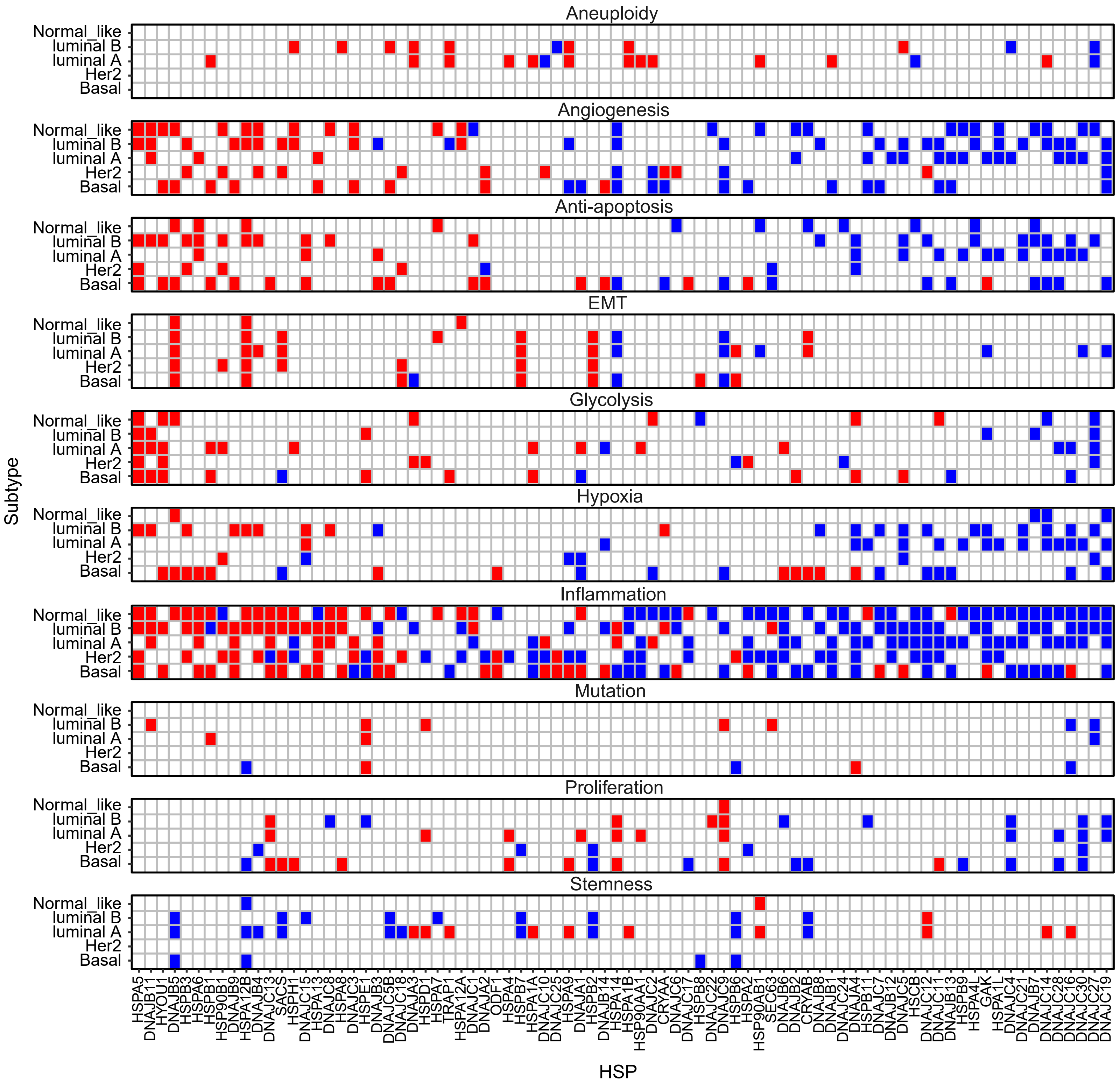

Figure S7. Associations between HSPs and hallmarks across breast cancer subtypes. Red cell and blue cell denote significantly positive and negative association, respectively.

Table S1. Samples across TCGA cancer types.

Cancer Type (Abbreviation)	Resource	Sample Size (Tumor)	Sample Size (Normal)
Adrenocortical carcinoma (ACC)	TCGA	79	0
Bladder Urothelial Carcinoma (BLCA)	TCGA	427	19
Breast invasive carcinoma (BRCA)	TCGA	1218	100
Cervical squamous cell carcinoma and endocervical adenocarcinoma (CESC)	TCGA	310	3
Cholangiocarcinoma (CHOL)	TCGA	45	9
Colon adenocarcinoma (COAD)	TCGA	329	41
Lymphoid Neoplasm Diffuse Large B-cell Lymphoma (DLBC)	TCGA	48	0
Esophageal carcinoma (ESCA)	TCGA	196	11
Glioblastoma multiforme (GBM)	TCGA	174	5
Head and Neck squamous cell carcinoma (HNSC)	TCGA	566	44
Kidney Chromophobe (KICH)	TCGA	91	25
Kidney renal clear cell carcinoma (KIRC)	TCGA	606	72
Kidney renal papillary cell carcinoma (KIRP)	TCGA	323	32
Acute Myeloid Leukemia (LAML)	TCGA	173	0
Brain Lower Grade Glioma (LGG)	TCGA	534	0
Liver hepatocellular carcinoma (LIHC)	TCGA	424	50
Lung adenocarcinoma (LUAD)	TCGA	576	58
Lung squamous cell carcinoma (LUSC)	TCGA	554	51
Mesothelioma (MESO)	TCGA	87	0
Ovarian serous cystadenocarcinoma (OV)	TCGA	309	0
Pancreatic adenocarcinoma (PAAD)	TCGA	183	4
Pheochromocytoma and Paraganglioma (PCPG)	TCGA	187	3
Prostate adenocarcinoma (PRAD)	TCGA	550	51
Rectum adenocarcinoma (READ)	TCGA	105	10
Sarcoma (SARC)	TCGA	265	2
Skin Cutaneous Melanoma (SKCM)	TCGA	474	0
Stomach adenocarcinoma (STAD)	TCGA	450	35
Testicular Germ Cell Tumors (TGCT)	TCGA	156	0
Thyroid carcinoma (THCA)	TCGA	572	57
Thymoma (THYM)	TCGA	122	2
Uterine Corpus Endometrial Carcinoma (UCEC)	TCGA	201	24
Uterine Carcinosarcoma (UCS)	TCGA	57	0
Uveal Melanoma (UVM)	TCGA	80	0

Table S2. Samples across GTEx tissues.

Tissue	Resource	Sample Size
Adrenal Gland	GTEx	204
Bladder	GTEx	11
Blood	GTEx	2561
Brain	GTEx	2076
Breast	GTEx	306
Cervix Uteri	GTEx	11
Colon	GTEx	539
Esophagus	GTEx	1111
Kidney	GTEx	50
Liver	GTEx	188
Lung	GTEx	607
Muscle	GTEx	718
Ovary	GTEx	138
Pancreas	GTEx	268
Prostate	GTEx	159
Skin	GTEx	1362
Stomach	GTEx	272
Testis	GTEx	284
Thyroid	GTEx	564
Uterus	GTEx	117

Table S3. HSP genes investigated in this study.

Symbol	Family	ENSEMBL ID
CRYAA	HSP20	ENSG00000160202
CRYAB	HSP20	ENSG00000109846
DNAJA1	HSP40(DNAJA)	ENSG00000086061
DNAJA2	HSP40(DNAJA)	ENSG00000069345
DNAJA3	HSP40(DNAJA)	ENSG00000103423
DNAJA4	HSP40(DNAJA)	ENSG00000140403
DNAJB1	HSP40(DNAJB)	ENSG00000132002
DNAJB11	HSP40(DNAJB)	ENSG00000090520
DNAJB12	HSP40(DNAJB)	ENSG00000148719
DNAJB13	HSP40(DNAJB)	ENSG00000187726
DNAJB14	HSP40(DNAJB)	ENSG00000164031
DNAJB2	HSP40(DNAJB)	ENSG00000135924
DNAJB3	HSP40(DNAJB)	ENSG00000227802
DNAJB4	HSP40(DNAJB)	ENSG00000162616
DNAJB5	HSP40(DNAJB)	ENSG00000137094
DNAJB6	HSP40(DNAJB)	ENSG00000105993
DNAJB7	HSP40(DNAJB)	ENSG00000172404
DNAJB8	HSP40(DNAJB)	ENSG00000179407
DNAJB9	HSP40(DNAJB)	ENSG00000128590
DNAJC1	HSP40(DNAJC)	ENSG00000136770
DNAJC10	HSP40(DNAJC)	ENSG00000077232
DNAJC11	HSP40(DNAJC)	ENSG00000007923
DNAJC12	HSP40(DNAJC)	ENSG00000108176
DNAJC13	HSP40(DNAJC)	ENSG00000138246
DNAJC14	HSP40(DNAJC)	ENSG00000135392
DNAJC15	HSP40(DNAJC)	ENSG00000120675
DNAJC16	HSP40(DNAJC)	ENSG00000116138
DNAJC17	HSP40(DNAJC)	ENSG00000104129
DNAJC18	HSP40(DNAJC)	ENSG00000170464
DNAJC19	HSP40(DNAJC)	ENSG00000205981
DNAJC2	HSP40(DNAJC)	ENSG00000105821
DNAJC21	HSP40(DNAJC)	ENSG00000168724
DNAJC22	HSP40(DNAJC)	ENSG00000178401
DNAJC24	HSP40(DNAJC)	ENSG00000170946
DNAJC25	HSP40(DNAJC)	ENSG00000059769
DNAJC27	HSP40(DNAJC)	ENSG00000115137
DNAJC28	HSP40(DNAJC)	ENSG00000177692
DNAJC3	HSP40(DNAJC)	ENSG00000102580
DNAJC30	HSP40(DNAJC)	ENSG00000176410
DNAJC4	HSP40(DNAJC)	ENSG00000110011
DNAJC5	HSP40(DNAJC)	ENSG00000101152
DNAJC5B	HSP40(DNAJC)	ENSG00000147570

Symbol	Family	ENSEMBL ID
DNAJC6	HSP40(DNAJC)	ENSG00000116675
DNAJC7	HSP40(DNAJC)	ENSG00000168259
DNAJC8	HSP40(DNAJC)	ENSG00000126698
DNAJC9	HSP40(DNAJC)	ENSG00000213551
GAK	HSP40(DNAJC)	ENSG00000178950
HSCB	HSP40(DNAJC)	ENSG00000100209
HSP90AA1	HSP90	ENSG00000080824
HSP90AB1	HSP90	ENSG00000096384
HSP90B1	HSP90	ENSG00000166598
HSPA12A	HSP70	ENSG00000165868
HSPA12B	HSP70	ENSG00000132622
HSPA13	HSP70	ENSG00000155304
HSPA14	HSP70	ENSG00000187522
HSPA1A	HSP70	ENSG00000204389
HSPA1B	HSP70	ENSG00000204388
HSPA1L	HSP70	ENSG00000204390
HSPA2	HSP70	ENSG00000126803
HSPA4	HSP70	ENSG00000170606
HSPA4L	HSP70	ENSG00000164070
HSPA5	HSP70	ENSG00000044574
HSPA6	HSP70	ENSG00000173110
HSPA7	HSP70	ENSG00000225217
HSPA8	HSP70	ENSG00000109971
HSPA9	HSP70	ENSG00000113013
HSPB1	HSP20	ENSG00000106211
HSPB11	HSP20	ENSG00000081870
HSPB2	HSP20	ENSG00000170276
HSPB3	HSP20	ENSG00000169271
HSPB6	HSP20	ENSG00000004776
HSPB7	HSP20	ENSG00000173641
HSPB8	HSP20	ENSG00000152137
HSPB9	HSP20	ENSG00000260325
HSPD1	HSP60	ENSG00000144381
HSPE1	HSP10	ENSG00000115541
HSPH1	HSP110	ENSG00000120694
HYOU1	HSP110	ENSG00000149428
ODF1	HSP20	ENSG00000155087
SACS	HSP40(DNAJC)	ENSG00000151835
SEC63	HSP40(DNAJC)	ENSG00000025796
TRAP1	HSP90	ENSG00000126602
